# Longitudinal Trends in Medicine Supply, Price and Utilisation in Primary Care Facilities in Rural Southwestern China Under National Essential Medicines Policy (2012-2017): Disparities Across Facilities and Medicines

**DOI:** 10.34172/ijhpm.8991

**Published:** 2025-11-18

**Authors:** Zhaohua Huo, Xuechen Xiong, Ge Bai, Jianchao Quan, Allen TC Lee, Linda CW Lam, Li Luo

**Affiliations:** ^1^Department of Psychiatry, Faculty of Medicine, The Chinese University of Hong Kong, Hong Kong SAR, China.; ^2^School of Public Health, The University of Hong Kong, Hong Kong SAR, China.; ^3^Department of Applied Social Sciences, The Hong Kong Polytechnic University, Hong Kong SAR, China.; ^4^School of Public Health, Fudan University, Shanghai, China.

**Keywords:** Drug Policy, Accessibility, Affordability, Evaluation, Inequity, Essential Medicines

## Abstract

**Background:**

The long-term impacts of the National Essential Medicines Policy (NEMP) in remote and rural areas of China remain underexplored. This study investigates the longitudinal trends in medicine supply, affordability, and utilisation in a rural county in Southwestern China from 2012 to 2017.

**Methods:**

A quasi-experimental design was employed to analyse 108 111 purchase records covering 650 medicines from seven township health centres (THCs) and 73 village clinics. Drug supply was assessed by the available number of medicine types, utilisation by sales volume, and affordability using a Drug Price Index (DPI) based on a fixed basket of 344 medicines. Interrupted time-series analysis was then conducted to evaluate trends during the first-stage NEMP and to identify changes following the implementation of second stage in November 2015.

**Results:**

Medicine number in rural primary care facilities increased steadily (+5.1 quarterly) during the first stage of NEMP, but exhibited abrupt and sustained drops (-2.1 quarterly) following the second-stage NEMP. DPIs exhibited gradual increases throughout both policy stages (+0.2% quarterly), while overall drug sales remained stable. Disaggregated by medicines, traditional Chinese medicines (TCMs) exhibited a faster increase in the available number than Western (chemical or biological) medicines and were associated with slower growth in DPI. By facility levels, village clinics experienced continuous declines in the number of Western medicines, accompanied by sharp increases in DPI. THCs also revealed a rising DPI in Western medicines, which consistently dominated overall drug sales.

**Conclusions:**

Primary care facilities in rural Southwestern China experienced relatively stable medicine supply and utilisation, alongside rising medicine prices, over the long-term implementation of NEMP. Significant disparities and structural changes across facility tiers and medicine categories underscore the urgent need to address the pressing challenges of the declining availability and increasing costs of Western medicines in rural village clinics.

## Background

**Key Messages**

**Implications for policy makers**

- The supply and sales of essential medicines in the rural primary care system remained relatively stable under the long-term National Essential Medicines Policy (NEMP), while medicine prices continued to steadily rise.
- By medicine category, traditional Chinese medicines (TCMs) demonstrated greater improvements in availability and experienced slower price increases compared to Western medicines.
- At the facility level, village clinics faced more significant challenges, including persistent declines in the variety of medicines and sharp price increases, particularly for Western medicines.
- An expanding inverted triangle pattern has formed, where higher-level healthcare facilities possess greater access to medicines, lower price increases and expanding utilisation.
- Policy adaptions should address the uneven impacts of NEMP across different categories of medicines and levels of healthcare providers within the rural primary care system.

**Implications for the public**

The National Essential Medicines Policy (NEMP) has been implemented in China for over 15 years, exerting significant influence on medicine utilisation and the broader healthcare system. While the policy has effectively curbed medicine prices and strengthened medicine supply, emerging evidence suggests unintended consequences, particularly in rural, remote, and underdeveloped areas. Our county-wide study in Southwestern China found that although the overall supply and utilisation of medicines in rural primary care remained relatively stable, notable disparities occurred across facility levels and medicine types. Specifically, village clinics experienced a continuous decline in the medicine supply, coupled with rising prices of Western pharmaceuticals. These findings underscore growing public concerns about the real-world and uneven impacts of NEMP on everyday medical care and highlight the need for policy refinements to ensure equitable access and affordability across all healthcare settings.

Primary care plays a vital role in population health by ensuring that patients receive accessible, high-quality, continuous, and efficient healthcare.^1^ Safe, effective and affordable medicines are essential to achieving the goals of primary care. However, medicine shortages, reflected in low availability, affordability and usage, remain a persistent challenge in many developing and resource-constrained regions.^2^ In rural and remote areas, these shortages are often exacerbated by underdeveloped transportation infrastructure, low purchasing power, insufficient health resources, and suboptimal prescription and adherence practices.^2,3^ As the global population continue to age, demand for quality and affordable medicines is expected to rise in the coming decades.^4^

 The World Health Organization (WHO) introduced the concept of essential medicines lists in 1975. Since then, these lists, together with essential medicines policies, have been widely adopted and have proven effective in improving the accessibility, safety, quality, and affordability of medicines.^5,6^ Since 1990s, China’s healthcare system has faced high medical costs and inefficiencies, primarily due to fragmented and weak regulatory frameworks.^7^ In response, China enacted comprehensive healthcare reforms in 2009, with the National Essential Medicines Policy (NEMP) as a central component.^8^ The NEMP established the National Essential Medicines Lists (NEML), centralized tendering, procurement and distribution systems, mandated the prescription of essential medicines, and implemented zero-markup pricing regulations.^8,9^ These measures have not only enhanced the availability, affordability and rational use of medicines in primary care, but have also helped control health expenditures, improve basic health services, and restructure the pharmaceutical market.^10-12^

 Despite such achievements, the NEMP in China has encountered several challenges. By 2018, pharmaceutical expenses still accounted for approximately one-third of total health spending in China, significantly higher than the average of 17% among Organisation for Economic Cooperation and Development countries.^13,14^ The top-down implementation of NEMP within a complex and adaptive system also triggered dynamic stakeholder responses, leading to unexpected and adverse policy outcomes.^15^ For instance, despite the establishment of NEML and centralized procurement systems, low-priced and paediatric medicines remain in short supply due to insufficient financial incentives for manufacturers and distributors.^16,17^ Furthermore, despite declining medicine prices, the availability of essential medicines has hardly improved even after a decade of policy implementation.^18^ Issues such as polypharmacy and over-prescription of antibiotics and injections persist, largely due to inadequate prescribing practices among healthcare providers.^12,19^ Zero-markup pricing regulation has imposed financial and operational strains on primary care facilities, undermining the continuity and quality of care.^20,21^

 More serious challenges stem from regional disparities in the impacts of NEMP. Compared to eastern, urban and more developed regions, the accessibility and affordability of medicines in western, remote and rural areas either stagnated or deteriorated following the implementation of NEMP.^18,22,23^ Medicine shortages were therefore most pronounced in these areas under NEMP, where supplying essential medicines often yields low or even negative profits for pharmaceutical providers.^21^ Limited information, insufficient training and the exclusive reliance on essential medicines contributed to suboptimal prescribing practices among village doctors, prompting patients to alter their healthcare-seeking behaviour and avoid primary care facilities.^21,24,25^

 Regarding the long-term sustainability of NEMP in rural regions, a previous study conducted in Southwestern China revealed uneven long-term trends in medicine availability and utilisation between primary and secondary care facilities.^26^ Notably, the availability and utilisation of essential medicines in primary care were less favourable than in secondary care, while drug prices revealed a consistent upward trend in both settings.^26^ Nevertheless, that study did not include village clinics, the most foundational level of healthcare delivery, thereby overlooking potential disparities in policy impacts between different tiers of primary care facilities – namely township health centres (THCs) and solo-practitioner-based village clinics.

 Our study examined the longitudinal trends of NEMP in terms of medicine supply, affordability and utilisation within the primary care system of a remote, rural and economically disadvantaged county in Southwestern China. The study period spanned from 2012 to 2017, covering year two to year seven following the policy’s implementation in 2010. Additionally, we explored variations in long-term trends across different facility levels and medicine categories.

## Research Questions and Hypotheses

 Question 1: What were the long-term trends of medicine supply, price and utilisation in the rural primary care system in Southwestern China under NEMP?

- Hypothesis 1.1: Primary care facilities exhibited increasing availability, decreasing prices and expanding utilisation in essential medicines over the long-term under NEMP.
- Hypothesis 1.2: Primary care facilities showed a tendency to use lower-priced drugs over the long run under NEMP.

Question 2: Were the long-term trends of medicine supply, price and utilisation consistent across different levels of primary care facilities and categories of medicines?

- Hypothesis 2.1: Long-term trends in medicine supply, utilisation and price under NEMP differed between THCs and village clinics.
- Hypothesis 2.2: Long-term trends in medicine supply, utilisation and price under NEMP varied between Western medicines and traditional Chinese medicines (TCMs).
- Hypothesis 2.3: Long-term trends in medicine supply, usage and price under NEMP varied by therapeutic classes of medicines.

Question 3: Did the expansion of the second-stage NEMP to secondary care (county hospitals) produce spillover effects on medicine supply, price and utilisation in primary care facilities?

- Hypothesis 3.1: The implementation of the second-stage NEMP in county hospitals altered the longitudinal trends in medicine supply, price and utilisation in primary care facilities.

## Methods

###  Study Design, Setting, and Policy

**Figure 1:**
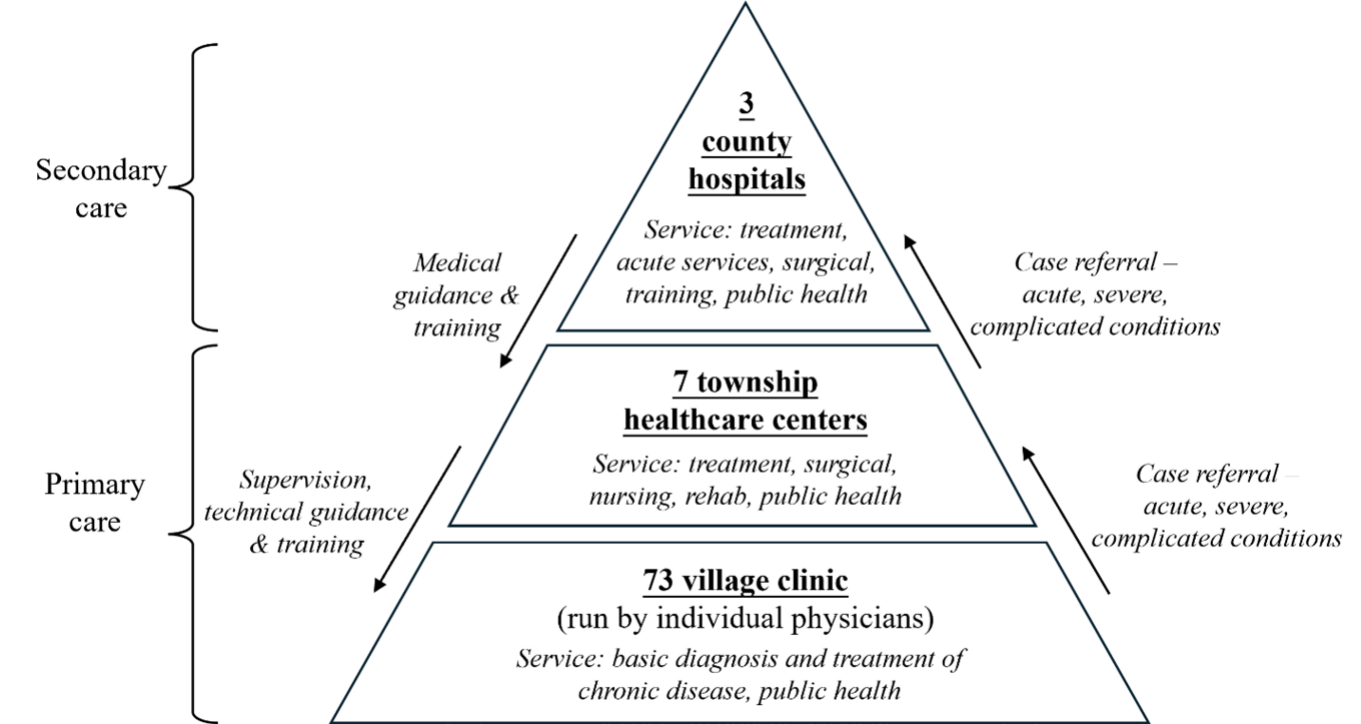
Three-Tiered Healthcare System in the Rural County.

 We employed a quasi-experimental design to investigate the long-term trends in drug supply, price and utilisation following the implementation of NEMP (2012-2017), with consideration of the second-stage policy impacts. The study was conducted in a rural, remote, and economically disadvantaged county in Yunnan province, Southwestern China, serving as a representative case for analysing NEMP as a complex social intervention at the micro-level.^26,27^ This county, characterised by 95% mountainous and plateau terrain and a 75% rural population, reported a per capita gross domestic products of US$ 4788 in 2020, equivalent to 70% of the provincial average and 53% of the national average.^27^ The local three-tiered healthcare system comprises 73 village clinics and 7 THCs at the primary care level, along with 3 county hospitals at the secondary care level (Figure 1).

 The NEMP was implemented in two stages in the studied county (Supplementary file 1, Table S1). The initial stage, from September 2010 to October 2015, targeted all primary care facilities and introduced centralized procurement, mandatory use of essential medicines (100%), and a zero-markup policy on retail medicine prices. The second stage, launched in November 2015, extended NEMP to secondary care facilities, incorporating centralized procurement, mandatory use of essential drugs (>50%), and zero-markup regulation. The study period was set from July 2012 to June 2017, capturing the stable implementation phase after the first-stage NEMP and the initial effects of the second-stage NEMP.

###  Data Collection

 Data were collected from all primary care facilities in the county, including seven THCs and 73 village clinics. Data from the three county hospitals were analysed in our prior work and were not included here.^26^ Drug procurement records in complete status (ie, successfully distributed to healthcare facilities) from July 2012 to June 2017 were extracted from the provincial electronic centralized procurement system. Records involving cancellations or failed distributions were excluded. Each procurement record described the date, generic name, dosage form, specification, quantity, wholesale price (ie, purchasing cost for healthcare facilities), and retail price (ie, selling price for patients) of a specific medicine. The overall monetary values recorded in the electronic system closely matched those reported in the financial reports of healthcare facilities (THCs: 98%; village clinics: 104%). After excluding invalid entries (eg, withdraw orders, incorrect information), 108 111 (95.3%) of 113 482 procurement records were included in analysis, with 23.6% from THCs and 76.4% from village clinics.

###  Drug Categorization

 Medicines were categorized based on their policy designation in the 2012 edition of the NEML: 430 as essential medicines and 45 as non-essential medicines (See Supplementary file 1, Table S2). Medicines were then further classified by ingredient type: Western (chemical or biological) medicines (n = 236), representing 61.6% of Western medicines listed in the NEML, and TCMs (n = 239), covering 86.6% of TCMs listed in the NEML. Thirteen therapeutical classification of Western medicines followed the WHO Anatomical Therapeutic Chemical system (See Supplementary file 1, Table S3).^28^ For standardization, a “unique” medicine was defined by its generic name, dosage form and specification at the minimum unit level (eg, Abacavir-Oral-0.3g).^26^ In total, 338 unique Western medicines (essential: 318; non-essential: 20) and 312 unique TCMs (essential: 286, non-essential: 26) were analysed.

###  Outcome Measures

 Drug supply was determined by the number of medicines available at healthcare facilities. Drug utilisation was assessed by sales amounts rather than defined daily dose (DDD) values, as DDDs were unavailable or invalid in 46.2% of Western medicines and all TCMs.^28^ Under the zero-markup policy, the retail price of medicines was equivalent to the procurement (wholesale) price. Regarding medicine affordability, Drug Price Index (DPI) was used to reflect cost variation of acquiring a fixed basket of medicines between the reporting and baseline period.^26,29^ Calculation of DPIs necessitates data for all observation points, and eventually 344 of 650 (52.9%) medicines with complete procurement records from 2012 to 2017 were included in price trend analysis.

 Three types of DPIs were computed: Laspeyres (DPI-L), Paasche (DPI-P) and Fisher (DPI-F).^11^ DPI-L (formula: *DPI*_L_ = ∑*P*_1_*Q*_0 _/ ∑*P*_0_*Q*_0_) measures the relative cost in purchasing the baseline quantities of goods at current versus baseline prices, while DPI-P (formula: *DPI*_p_ = ∑*P*_1_*Q*_1 _/ ∑*P*_0_*Q*_1_) measures the relative cost in purchasing the current quantities of goods. In these formulas, *P*_0_ and *P*_1_ represent the price of a product in the baseline and reporting period, respectively, while *Q*_0_ and *Q*_1_ denote the corresponding quantities. Both of DPI-L and DPI-P are biased due to their assumption of unchanged consumption, and DPI-F, calculated as the geometric average of the two (formula: $DPI_{F}=\sqrt{DPI_{L}\times DPI_{p}}$ ), accounts for changes in both price and quantity.^30^

 A DPI > 1 indicates increased costs, DPI < 1 indicates decreased costs, and DPI = 1 reflects stable costs. Comparisons across different DPI types can reveal shifts in medicine consumption patterns. Specifically, a result where DPI-F < DPI-L (or DPI-F > DPI-P) suggests increased use of lower-priced drugs, and vice versa. The DPIs themselves reflect the inflation of medicine prices, and were compared against local inflation indicators, including Consumer Price Index (CPI) and pharmaceutical Producer Price Index (PPI).

###  Statistical Analysis

 Pharmaceutical data were analysed on a quarterly basis, yielding 20 observation points between July 2012 and June 2017, also in alignment with the procurement cycle of healthcare facilities (every three months). Descriptive analyses were conducted to demonstrate the number of available medicines, sales volumes, and DPIs, stratified by facility type and medicine category.

 Interrupted time-series analysis (ITSA) was employed to assess the longitudinal trends of following first-stage NEMP and to evaluate the impacts of second-stage expansion.^31,32^ Accounting for a three-month policy lag, the intervention point was set at January 2016. Segmented linear regression models were specified as follows: *Y*_t_*= β*_0_ + *β*_1_T + *β*_2_*X*_1_t_ + *β*_3_*TX*_1_t_ + *ε*_t_, where *Y*_t_denotes the outcome at time *T*, *T* denotes the time since the start of observation, and *X*_1_t_ is a binary indicator for the implementation of second-stage NEMP. Coefficient *β*_0_ and *β*_1_ estimate the baseline level and trend under the first-stage NEMP, while *β*_2_ and *β*_3_ estimate the changes in level and trend following the second-stage NEMP. Drug sales were log-transformed to normalise distribution in ITSA. Autocorrelation was corrected using the Prais-Winsten method, and seasonality was adjusted by including quarterly indicators.^33,34^

 Two group ITSA was used to examine differences in trends by facility level (THCs vs. village clinics) and medicine type (Western vs. TCM), with an extended model of *Y*_t_* = β*_0_ + *β*_1_T + *β*_2_*X*_1_t_ + *β*_3_*TX*_1_t_ + *β*_4_*G + β*_5_*G. Tε*_t_*+ β*_6_*G. X*_1_t_*+ β*_7_*G. TX*_1_t_* + ε*_t_, where *G* is the group indicator, and *β*_4_ to *β*_7_ capture group differences. All statistical analyses were conducted using R version 4.3 (package: “*prais ”*), with a significance level set at *P* value <.05.

## Results

###  Descriptive Analysis

 Between 2012 and 2017, the primary care system experienced slight increases in the number of available medicines from 434 to 447 (+3.0%), accompanied by a 6.4% rise in DPI-F and a 6.3% increase in total medicine spending (from US$ 0.32 million to US$ 0.34) (Supplementary file 2, Table S4). Broken down by medicine types, Western medicines and TCMs showed contrasting trends in the available number of type (-9.4% vs. +17.6%) and price (+11.4% vs. +1.8%). By facility level, THCs experienced growth in both the number of medicines and total sales, while in contrast village clinics faced declines in both areas. Notably, medicine prices rose more rapidly in village clinics (+11.6%) than in THCs (+4.3%) (Supplementary file 2, Table S4).

###  Number of Medicines

**Figure 2:**
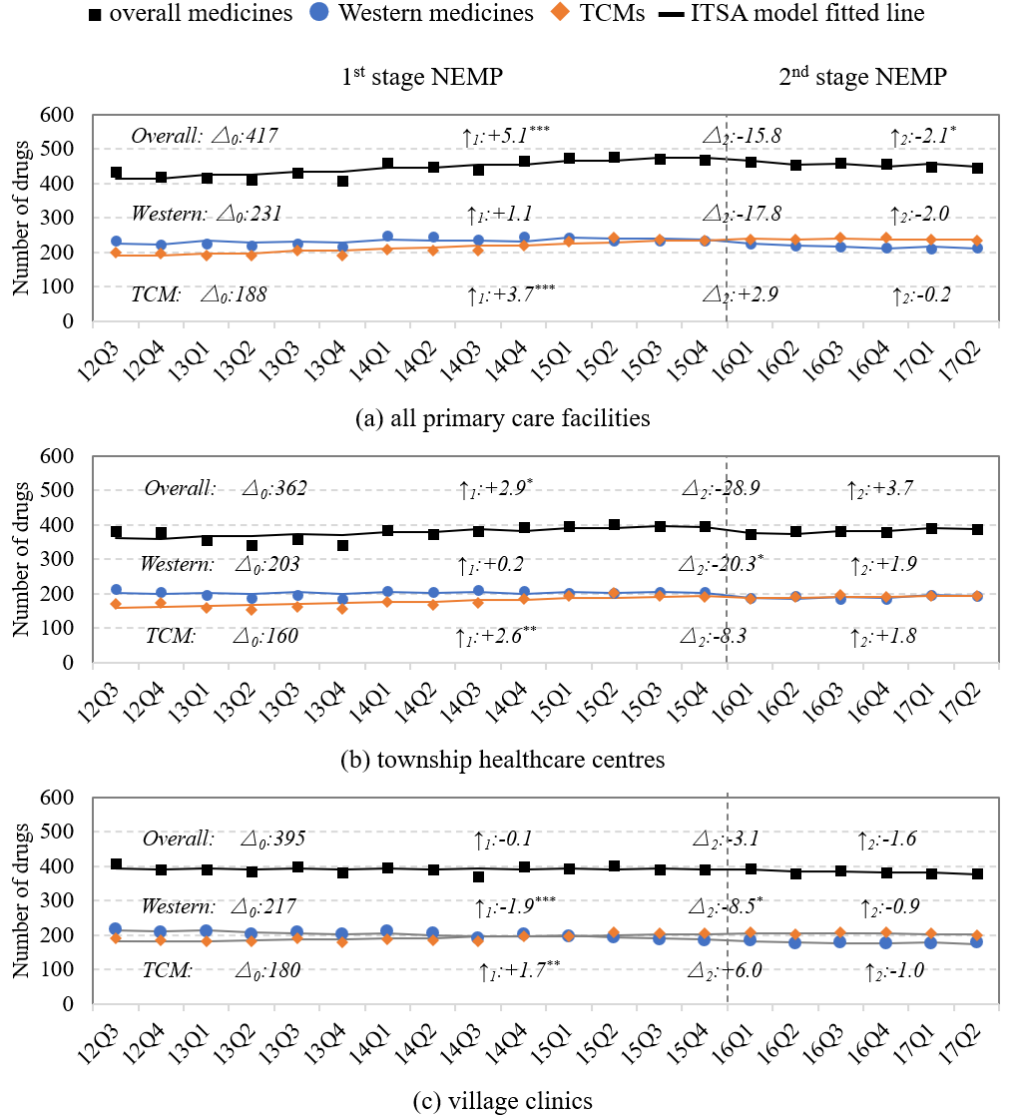
Number of Medicines by Facility Level and Medicine Type.Abbreviations: ITSA, interrupted time-series analysis; NEMP, National Essential Medicines Policy; TCM, traditional Chinese medicine. ■: Observed number of overall medicines; ● Observed number of Western medicines; ♦: Observed number of TCMs; ▬: Model fitted line for medicine number in ITSA; △_0_: INITIAL level (β_0_); ↑_1_: Sustained change per quarter during 1st-phase NEMP (β_1_ in ITSA); △_2_: Immediate change after 2nd-phase NEMP (β_2 _in ITSA); ↑_2_: Sustained change per quarter after 2nd-phase NEMP (β_1_+β_3 _in ITSA). * *P* < .05; ** *P* < .01; *** *P* < .001.

 The number of available medicines increased during the first-stage NEMP, but declined following the second-stage, with variations observed across medicine categories and facility levels (Table and Figure 2). By medicine type, available number of TCMs experienced faster growth than Western medicines during the first-stage NEMP (+3.7 vs. +1.1, *P* = .008), and also showed lesser declines after the second stage (Figure 2a; Supplementary file 2, Table S5). Meanwhile, the number of non-essential medicines (both Western medicines and TCMs) increased more rapidly than that of essential medicines during the first-stage policy (*P* = .008) (Supplementary file 2, Table S5, Figure S1).

**Table:** Summary of Findings Based on Interrupted Time-Series Analysis

**ITSA Estimators by Quarter** **(95% Confidence Interval, ** ***P*** ** Value)**	**First-Stage NEMP (July 2012 – December 2015)**		**Second-Stage NEMP (January 2016 – June 2017)**	
	**Initial Level (β1)**	**Initial Trend (β2)**	**Change in Level (β3)**	**Change in Trend (β4)**
**Types of medicines**	(number)	(number)	(number)	(number)
All primary care facilities	417 (400, 433), *P* < .001	5 (3, 7), *P* < .001	-16 (-43, 12), *P *= .278	-7 (-14, -1), *P *= .044
Essential medicines	418 (407, 430), *P* < .001	2 (0, 3), *P *= .015	-5 (-24, 14), *P *= .602	-4 (-8, 0), *P *= .100
Non-essential medicines	-1 (-10, 9), *P *= .889	3 (2, 4), *P* < .001	-10 (-23, 4), *P *= .181	-3 (-7, 0), *P *= .105
Western medicines	231 (219, 243), *P* < .001	1 (0, 2), *P *= .112	-18 (-37, 1), *P *= .093	-3 (-8, 1), *P *= .207
TCMs	188 (175, 200), *P* < .001	4 (2, 5), *P* < .001	3 (-17, 23), *P *= .782	-4 (-9, 1), *P *= .147
THCs	362 (339, 385), *P* < .001	3 (0, 6), *P *= .046	-29 (-64, 6), *P *= .131	1 (-8, 10), *P *= .867
Essential medicines	363 (345, 381), *P* < .001	0 (-2, 2), *P *= .683	-19 (-47, 9), *P *= .199	4 (-3, 11), *P *= .278
Non-essential medicines	-1 (-10, 9), *P *= .889	3 (2, 4), *P* < .001	-10 (-23, 4), *P *= .181	-3 (-7, 0), *P *= .105
Western medicines	203 (191, 214), *P* < .001	0 (-1, 2), *P *= .708	-20 (-39, -2), *P *= .048	2 (-3, 6), *P *= .477
TCMs	160 (145, 176), *P* < .001	3 (1, 4), *P *= .013	-8 (-30, 13), *P *= .466	-1 (-7, 5), *P *= .779
Village clinics	395 (385, 406), *P* < .001	0 (-1, 1), *P *= .903	-3 (-20, 13), *P *= .715	-2 (-5, 2), *P *= .431
Western medicines	217 (212, 222), *P* < .001	-2 (-2, -1), *P* < .001	-9 (-15, -2), *P *= .029	1 (-1, 3), *P *= .235
TCMs	180 (170, 190), *P* < .001	2 (1, 3), *P *= .011	6 (-10, 22), *P *= .469	-3 (-6, 1), *P *= .201
**Sales of medicines**	(US$ 1000)	(%)	(%)	(%)
All primary care facilities	348 (308, 394), *P* < .001	0.0 (-1.2, 1.2), *P *= 1.000	15.9 (-5.3, 41.8), *P *= .175	-1.7 (-6.2, 2.9), *P *= .477
Essential medicines	349 (309, 394), *P* < .001	-0.1 (-1.3, 1.1), *P *= .852	14.1 (-6.3, 39.0), *P *= .211	-1.7 (-6.0, 2.9), *P *= .478
Non-essential medicines	0 (0, 0), *P* < .001	52.7 (37.3, 69.9), *P* < .001	32.1 (-67.2, 432.4), *P *= .702	-31.6 (-52.2, -2.1), *P *= .058
Western medicines	186 (163, 213), *P* < .001	-0.2 (-1.4, 1.1), *P *= .803	16.0 (-6.2, 43.4), *P *= .195	-0.5 (-5.2, 4.5), *P *= .841
TCMs	162 (143, 182), *P* < .001	0.2 (-1.0, 1.5), *P *= .723	15.2 (-5.9, 41.1), *P *= .194	-3.1 (-7.5, 1.5), *P *= .208
THCs	165 (141, 194), *P* < .001	0.0 (-1.7, 1.8), *P *= .974	32.3 (0.9, 73.4), *P *= .064	-2.0 (-8.0, 4.4), *P *= .542
Essential medicines	166 (142, 193), *P* < .001	-0.2 (-1.8, 1.4), *P *= .802	29.0 (-0.4, 67.2), *P *= .076	-2.0 (-7.7, 4.1), *P *= .528
Non-essential medicines	0 (0, 0), *P* < .001	52.7 (37.3, 69.9), *P* < .001	32.1 (-67.2, 432.4), *P *= .702	-31.6 (-52.2, -2.1), *P *= .058
Western medicines	90 (75, 108), *P* < .001	0.4 (-1.6, 2.4), *P *= .713	35.6 (0.8, 82.4), *P *= .065	-0.3 (-7.3, 7.1), *P *= .926
TCMs	75 (63, 89), *P* < .001	-0.5 (-2.1, 1.2), *P *= .580	28.4 (-3.1, 70.1), *P *= .105	-4.3 (-10.3, 2.1), *P *= .208
Village clinics	183 (160, 208), *P* < .001	0.0 (-1.2, 1.2), *P *= .977	0.5 (-18.0, 23.0), *P *= .964	-1.1 (-5.6, 3.6), *P *= .650
Western medicines	96 (82, 113), *P* < .001	-0.7 (-2.0, 0.6), *P *= .321	-5.3 (-24.8, 19.3), *P *= .653	-0.3 (-5.4, 5.2), *P *= .926
TCMs	86 (76, 98), *P* < .001	0.8 (-0.5, 2.1), *P *= .234	5.7 (-14.4, 30.4), *P *= .617	-2.2 (-6.8, 2.7), *P *= .384
**Drug price **(Essential medicines)	(DPI-F)	(DPI-F)	(DPI-F)	(DPI-F)
All primary care facilities	99.6 (98.1, 101.1), *P* < .001	0.2 (0.1, 0.4), *P *= .016	1.5 (-1.0, 4.1), *P *= .260	0.0 (-0.6, 0.6), *P *= .968
Western medicines	100.4 (97.0, 103.7), *P* < .001	0.3 (0.0, 0.7), *P *= .096	3.0 (-2.5, 8.5), *P *= .299	-0.1 (-1.4, 1.3), *P *= .942
TCMs	98.8 (97.9, 99.7), *P* < .001	0.2 (0.1, 0.3), *P *= .002	0.0 (-1.5, 1.5), *P *= .988	0.0 (-0.3, 0.4), *P *= .854
THCs	100.4 (98.2, 102.5), *P* < .001	0.1 (-0.1, 0.3), *P *= .373	2.5 (-1.1, 6.1), *P *= .193	0.1 (-0.7, 0.9), *P *= .806
Western medicines	102.2 (97.6, 106.8), *P* < .001	-0.1 (-0.6, 0.3), *P *= .603	7.1 (-0.3, 14.5), *P *= .083	0.3 (-1.4, 2.0), *P *= .701
TCMs	98.8 (97.0, 100.6), *P* < .001	0.3 (0.1, 0.4), *P *= .010	-0.9 (-3.8, 2.0), *P *= .561	-0.3 (-0.9, 0.4), *P *= .472
Village clinics	99.7 (98.5, 100.9), *P* < .001	0.4 (0.3, 0.5), *P* < .001	0.6 (-1.5, 2.6), *P *= .582	0.2 (-0.3, 0.7), *P *= .441
Western medicines	100.4 (97.7, 103.1), *P* < .001	0.7 (0.4, 1.0), *P* < .001	0.6 (-4.0, 5.2), *P *= .805	0.0 (-1.1, 1.1), *P *= .992
TCMs	99.2 (98.3, 100.1), *P* < .001	0.2 (0.1, 0.3), *P *= .009	0.7 (-0.5, 2.0), *P *= .264	0.2 (-0.1, 0.6), *P *= .211

Abbreviations: DPI-F, Fisher Drug Price Index; ITSA, interrupted time-series analysis; NEMP, National Essential Medicines Policy; TCMs, traditional Chinese medicines; THCs, township healthcare centres.

 By facility level, village clinics exhibited significantly different trends in the medicine number compared to THCs during the first-stage policy. For all medicines, the change was -0.1 per quarter in village clinics versus +2.9 in THCs (*P* < .001); for Western medicines: -1.9 vs. +0.2 (*P* < .001); and for TCMs: +1.7 vs. +2.6 (*P* = .012) (Figure 2b-2c; Supplementary file 2, Table S5).

 By therapeutic classifications, medicine number growth during the first-stage NEMP was observed in medicines treating for (*i*) alimentary tract and metabolism, (*ii*) Musculo-skeletal system, (*iii*) nervous system, (*iv*) respiratory system, and (*v*) various TCMs (Supplementary file 3, Figure S2, Table S6). However, these were followed by either stable or declining trends after the second-stage. In contrast, village clinics experienced long-term declines in the number of Western medicines for (*i*) alimentary tract and metabolism, (*ii*) blood and blood-forming organs, (*iii*) dermatological, (*iv*) anti-infectives, (*v*) nervous system, and (*vi*) sensory organs (Supplementary file 3, Figure S2, Table S6).

###  Sales of Medicines

**Figure 3:**
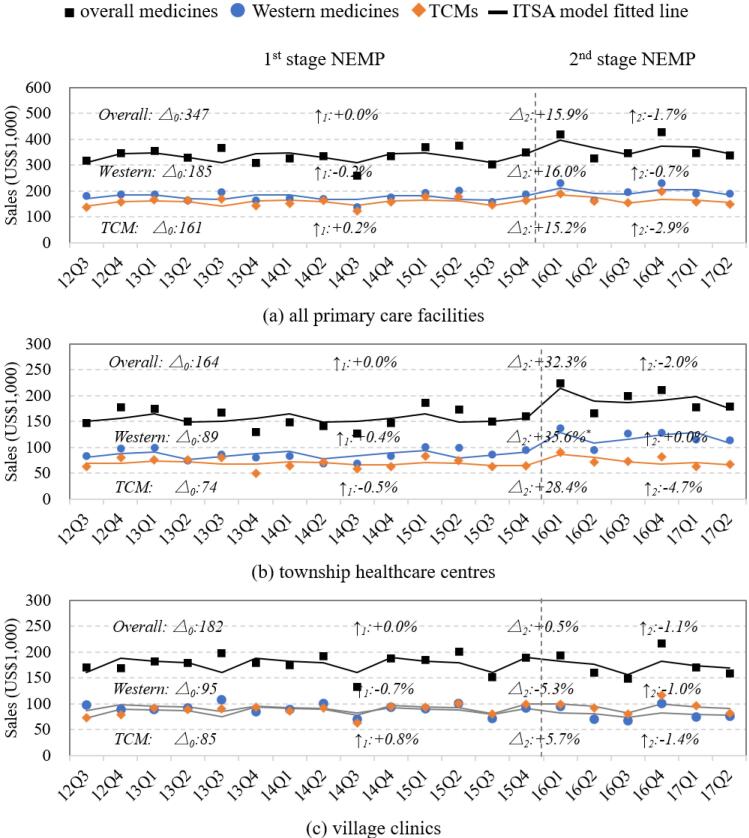
Sales of Medicines by Facility Level and Medicine Type.Abbreviations: ITSA, interrupted time-series analysis; NEMP, National Essential Medicines Policy; TCM, traditional Chinese medicine. ■: Observed sales volume of overall medicines; ●: Observed sales volume of Western medicines; ♦: Observed sales volume of TCMs; ▬: Model fitted line for sales volume in ITSA; △_0_: Initial level (β_0_ in ITSA); ↑_1_: Sustained change per quarter during 1st-phase NEMP (β_1_ in ITSA); △_2_: Immediate change after 2nd-phase NEMP (β_2_ in ITSA); ↑_2_: Sustained change per quarter after 2nd-phase NEMP (β_1_+β_3_ in ITSA). * *P* < .05; ** *P* < .01; *** *P* < .001.

 Overall medicine expenditures in primary care facilities remained relatively stable and showed no significant trends throughout the observation period (Figure 3a). A non-significant and abrupt increase (+15.9%, *P* = .175) was observed following the implementation of second-stage NEMP, primarily driven by THCs (Table). No significant differences in sales trends were found between THCs and village clinics (Figure 3b-3c; Supplementary file 2, Table S5). However, the growth in medicine sales during the first-stage NEMP in THCs was primarily driven by (non-essential) Western medicines, whereas in village clinics, the growth was mainly attributed to TCMs (Supplementary file 2, Table S5, Figure S1).

 By therapeutic classifications, THCs experienced structural shifts in medicine sales during the first-stage NEMP, including increases in (*i*) cardiovascular system, (*ii*) dermatological, (*iii*) Genito-urinary and sex hormones, (*iv*) nervous system, and (*v*) respiratory system medicines, accompanied by declines in antiparasitic and sensory organ medicines (Supplementary file 3, Figure S3, Table S7). Following the second-stage NEMP, THCs exhibited abrupt sales growth across most medicine categories, which were followed by declining trends. In village clinics, sales increases during first-stage NEMP were not sustained following the second-stage policy, particularly in medicines for (*i*) cardiovascular, (*ii*) dermatological, (*iii*) Genito-urinary and sex hormones, (*iv*) systemic hormones, (*v*) Musculo-skeletal diseases, and (*vi*) TCMs related to orthopaedics and ear, nose and throat (Supplementary file 3, Figure S3, Table S7).

###  Price of Medicines

**Figure 4:**
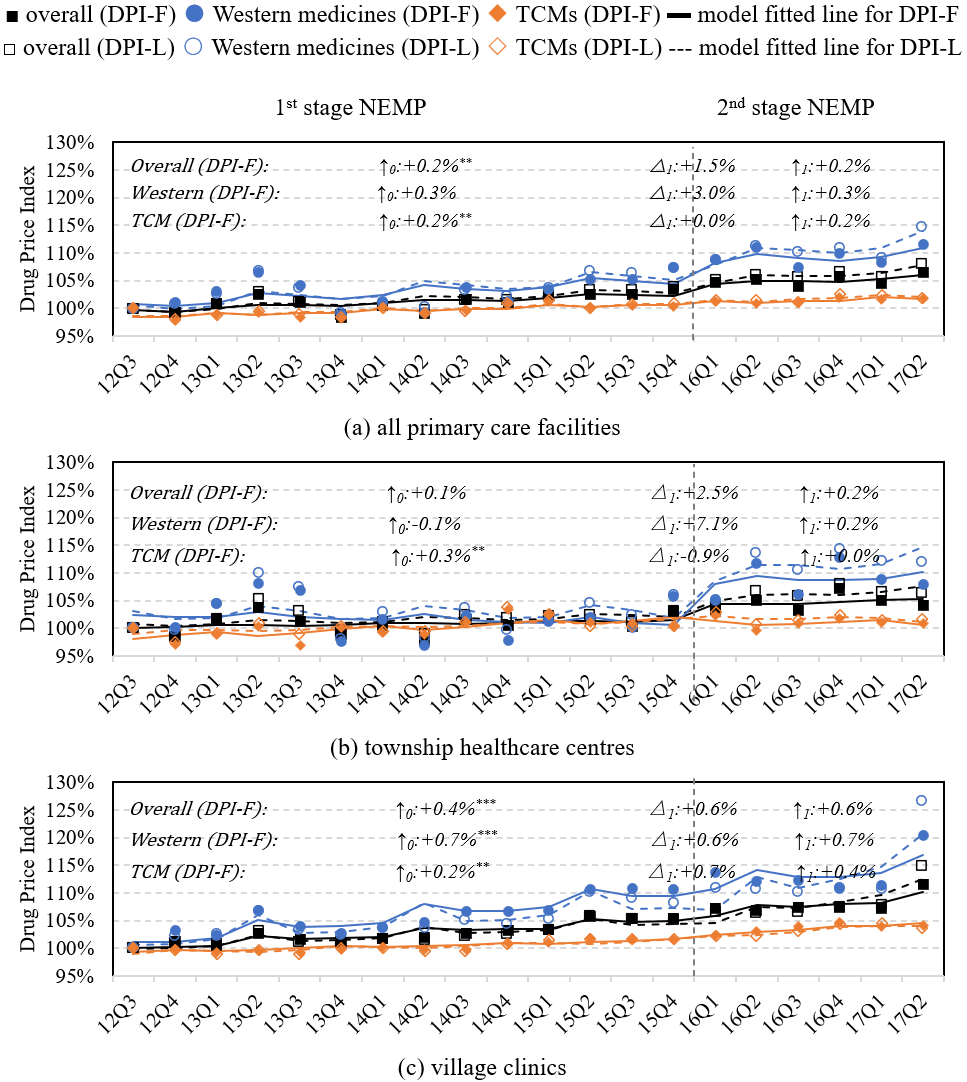
Retail Price of Medicines by Facility Level and Essential Medicine List.Abbreviations:DPI-F, Fisher Drug Price Index; DPI-L, Laspeyres Drug Price Index; NEMP, National Essential Medicines Policy; TCM, traditional Chinese medicine. ■: DPI-F of overall medicines; ●: DPI-F of Western medicines; ♦: DPI-F of TCMs; ▬: model fitted line for DPI-F in ITSA; □: DPI-L of overall medicines; ○: DPI-L of Western medicines; ◊: DPI-L of TCMs; ---: model fitted line for DPI-L in ITSA; △_0_: initial level (β_0 _in ITSA); ↑_1_: sustained change per quarter during 1st-phase NEMP (β_1_ in ITSA); △_2_: immediate change after 2^nd^-phase NEMP (β_2_ in ITSA); ↑_2_: sustained change per quarter after 2nd-phase NEMP (β_1_+β_3_ in ITSA). * *P* < .05; ** *P* < .01; *** *P* < .001.

 The DPI of all medicines in the primary care system showed a sustained upward trend during both stages of NPEM, increasing by 0.2% per quarter (*P* = .016) (Table and Figure 4a). Village clinics experienced a significantly higher rate of DPI compared to THCs (per quarter: +0.4% vs. +0.1%, *P* = .019), particularly in terms of Western medicines (per quarter: +0.7% vs. -0.1%, *P* = .003) (Figure 4b-4c; Supplementary file 2, Table S5). Within village clinics, Western medicines also exhibited significantly greater price increases than TCMs (per quarter: +0.7% vs. +0.2%, *P* = .002) (Figure 4c; Supplementary file 2, Table S5).

 By therapeutic classification, THCs experienced price increases during the first-stage NEMP in medicines for: (*i*) blood and blood-forming organs, (*ii*) anti-infectives, (*iii*) nervous system, (*vi*) respiratory system, and (*v*) TCMs (internal medicine). The only notable price decline was observed in TCMs for ear, nose and throat conditions. After the second-stage NEMP, significant price increases were recorded in medicines of alimentary tract and metabolism and TCMs of ear, nose and throat, while significant decreases were observed in medicines of nervous system (Supplementary file 4, Figure S4, Table S8).

 In village clinics, most Western medicines exhibited rising price trends during the first-stage policy, with the exception of Musculo-skeletal drugs, which saw a long-term price decline. Following the second-stage policy, cardiovascular medicines exhibited significant price increases. Furthermore, internal TCMs experienced moderate price increases throughout the observation course, while prices of other TCMs remained relatively stable (Supplementary file 4, Figure S4, Table S8).

 A comparison between DPI-F (solid line) and DPI-L (dotted line) revealed that THCs tended to prescribe lower-priced medicines (DPI-F < DPI-L) over the long-term of NEMP. However, this trend did not present in village clinics (DPI-F > DPI-L) (Figure 4). Comparing the local CPI and the pharmaceutical PPI, the increase in DPI for overall primary care facilities was slower than the local CPI, but faster than the pharmaceutical PPI (Supplementary file 2, Table S4). Notably, village clinics exhibited a DPI that surpassed the local CPI following the implementation of the second-stage policy.

## Discussion

###  Main Findings

####  Changing Number and Structure in Medicine Supply

 Our study evaluated the long-term trends of NEMP within the primary care system of a deprived rural county in Southwestern China, with a summary of our findings is presented in Table. During the first-stage NEMP, the number of available medicine types showed an increasing and stable trend in THCs and village clinics, respectively. This suggests that the primary care facilities were gradually adapting to the policy, with the number of essential medicines approaching a threshold suitable for routine clinical use (covering approximately 72% of NEML). However, this stability did not persist into the second-stage NEMP. Declining trends emerged, particularly in the availability of Western medicines and in village clinics. The reasons behind these declines are multifaceted.

 From the perspective of manufacturers and distributors, low profit margins and high transportation cost discouraged the delivery of small quantities of essential Western medicines to remote and mountainous primary healthcare facilities.^16,17^ For primary care physicians, motivations to stock and prescribe essential medicines were undermined by the loss of income from medicine sales due to zero markup regulation, the high cost of maintaining stock and insufficient training in prescribing essential drugs.^20,35,36^ Additionally, physicians faced an increased workload of public health responsibilities introduced by broader health reforms. Their incomes, largely reliant on government subsidies, were fixed by local population and geographic area rather than actual clinical service volume. Medical service prices in the primary care system have also remained unchanged for years. These factors collectively contributed to the reduced willingness among primary care physicians to provide medical care and prescribe essential medications.

 When our findings are integrated with evidence from secondary care facilities in the previous study,^26^ the structural shift in medicine supply across different levels of care under NEMP becomes evident. From the county perspective, the gap widened in medicine supply between primary and secondary care facilities (overlapping medicines declined from 32% in 2012 to 28% in 2016) (Supplementary file 5, Figure S5). County hospitals increasingly supplied diverse Western medicines (sole supply rose from 57% to 65%, with around 60% being non-essential), while primary care facilities shifted toward TCMs (sole supply increased from 23% to 36%). Within the primary care level, divergence also grew (overlapping medicines dropped from 86% in 2012 to 74% in 2016) (Supplementary file 5, Figure S6). THCs, in contrast to village clinics, demonstrated a broader provision of both Western medicines (sole supply rose from 8% to 16%, with ~50% being non-essential) and TCMs (sole supply increased from 4% to 16%). Meanwhile, both the supply and sales volume of originator (brand-name) medicines, typically associated with higher prices, were significantly expanding and exhibited a yearly increase in county hospitals, compared to primary care facilities (Supplementary file 5, Figure S7). The limited use of originator medicines in primary care aligns with policy expectations, which emphasise the prioritization of affordable, lower-cost essential medicines within primary healthcare facilities. These overall widening disparities underscore the disproportional long-term impacts of NEMP across different tiers of the healthcare system, with higher-level facilities exhibiting greater access to a wider variety of medicines, particularly Western and brand-name medicines.

####  Uneven Price Hikes in Western Medicines and Village Clinics

 While medicine prices generally trended upward over the long-term following NEMP implementation, significant imbalances were observed across facility types and medicine categories (Table). First, while both Western medicines and TCMs experienced price increases, the increase rate was notably higher for Western medicines, especially after the second-stage NEMP. Second, while both THCs and village clinics saw price rises, the growth rate was significantly higher in village clinics, particularly for Western medicines. Third, while overall price growth at primary care facilities fell between local customer and producer inflation rates, the price increases for Western medicine in village clinics surpassed local CPI after the second-stage policy. This suggests the partial containments of medication costs along the supply chain from manufacturers to healthcare providers and patients.

 These findings highlight the persistent challenge of controlling drug prices under NEMP, particularly in rural areas.^26,37^ One key factor was the price deregulation after 2015, which lifted government-imposed price caps on low-cost medicines to encourage production and alleviate shortages.^38^ The policy adjustment likely contributed to widespread price increases, not only observed in primary care facilities but also in secondary care facilities, as shown in our previous study.^26^ Another contributing factor is the supply shortage of lower-priced medicines and alternative use of higher-priced medicines in rural primary care. As manufacturers were disincentivised from producing and distributing low-profit drugs, healthcare providers were compelled to select alternatives or reintroduce versions of the same medicines.^26^ These alternatives were produced by different manufacturers (bidders), with varying dosages or packaging, but were also assigned with higher unit prices. This practical shift may explain the particularly steep price increases for Western medicines, which are most exposed to competitive bidding markets, and in village clinics, which are the most remote and incur the highest transportation costs and lowest profits for manufacturers.

####  Widening Gaps in Medicine Utilisation and Sales Across Facilities

 Despite notable changes in medicine availability and pricing, overall sales volumes remained relatively stable, indicating limited growth in actual medicine utilisation within the primary care system. In THCs, a sharp increase in drug sales was observed only after the implementation of the second-stage NEMP, primarily driven by increased use of Western and non-essential medicines. Village clinics maintained steady sales for both Western medicines and TCMs, despite significant price increases. Drawing on evidence from our previous study,^26^ secondary care facilities (ie, county hospitals) demonstrated a consistent upward trend in total medicine sales before and after the second-stage NEMP, with an average quarterly growth rate of +3.3%.

 The divergent trends in medicine utilisation and sales across different tiers of healthcare facilities reflect the evolving dynamics of patient health seeking behaviour and medical demands.^26,39^ Notably, despite a relatively stable local population of approximately 185 000 during the study period, a marked shift in patient flow was observed from village clinics to THCs, and from primary care to secondary care institutions. In village clinics, annual clinical visits declined sharply from over 250 000 in 2012 to 190 000 in 2016, marking a 24% decrease. In contrast, THCs experienced a steady rise in patient visits from approximately 113 000 in 2012 to 150 000 in 2016 with a 33% growth. County hospitals also saw a substantial increase, with annual visits rising by 32%, from 190 000 in 2012 to over 251 000 in 2016. Over the same period, the distribution of overall medical visits across the whole county shifted across village clinics (from 45% to 32%), THCs (from 20% to 25%), and county hospitals (from 34% to 44%). The trend further reinforced the “inverted triangle” pattern within the county-wide healthcare system, underscoring the diminishing role of grassroots-level village clinics in providing basic clinical services. In particular, following the implementation of the second-stage NEMP, the average retail prices of medicines in secondary care facilities decreased by 7% under the zero-markup pricing policy, further incentivising patients to seek care at county hospitals for a broader selection of medicines, lower costs, and higher-quality services.^26^

 Although not included in our analysis, private entities, such as private medicine outlets, pharmacies and hospitals, also play both complementary and competitive roles in relation to public services. Their wider selection of branded medicines, more flexible supply chains, and differentiated pricing strategies, can significantly influence overall market dynamics, shaping both demand and supply within the public healthcare sector.^40^ In rural, remote and under-resourced regions, private pharmacies can serve as critical alternatives for patients, especially when their concerns arise on the limited variety or perceived quality of medicines in public facilities.^15,40^ In such contexts, shifts in patient preference and informal referrals toward the private sectors may reduce their demand for services and essential medicines at the primary care level, potentially reinforcing the vicious cycle of undersupply and underutilisation in village clinics observed within our study area.

####  Therapeutic Class-Specific Changes in Medicine Market

 Notable changes were observed across specific therapeutic drug classifications. In terms of drug supply, sustained declines among primary care facilities were recorded in Western medicines related to (*i*) alimentary tract and metabolism, (*ii*) blood and blood-forming organs, (*iii*) dermatology, (*iv*) nervous system, and (*v*) sensory organs. In contrast, declining number of medicines in genitourinary system and sex hormone was observed in county hospitals.^26^

 Regarding prices, cardiovascular medicines in village clinics rose sharply following the implementation of the second-stage NEMP, mainly due to the introduction of new version of pharmaceuticals by various manufacturers. Price increases were also observed across both THCs and village clinics of medicines targeting: (*i*) blood and blood-forming organs, (*ii*) dermatological conditions, (*iii*) genitourinary system and sex hormones, (*iv*) nervous system, and (*v*) respiratory system. Additionally, village clinics experienced notable price hikes in anti-infective, antiparasitic, and sensory organ medicines. At the county hospital level, price increases were mainly observed in (*i*) alimentary tract and metabolism, (*ii*) dermatological, (*iii*) genitourinary and sex hormones, (*iv*) systemic hormonal, and (*v*) antiparasitic medicines, after removing the impacts of zero-markup regulation during the same period.^26^

 In terms of utilisation, THCs saw declining sales in antiparasitic and sensory organ medicines, while village clinics experienced reduced usage of medicines for blood and blood-forming organs. Sales in surgical TCM also declined in both levels of primary care facilities. In county hospitals, significant decreases in sales were observed in antiparasitic products and also surgical, gynaecological and orthopaedic TCMs.^26^ Such disproportionate changes in medicine utilisation across therapeutic categories could be driven by a complex interplay of factors, including evolving epidemiological trends, shifts in the disease spectrum, and the medical behaviours of healthcare providers and patients in response to changes in medicine availability and pricing.^12,19^

###  Strengths and Limitations

 This study is among the few to examine the long-term trends of NEMP on primary care in Southwestern China. The selection of a rural, remote, and deprived county as the study site, minimises the influence of external confounding factors, thereby offering a clearer view of the policy’s true impact.^26^ The findings also underscore the dynamic and uneven effects of the NEMP across different levels of primary care facilities (THCs vs. village clinics) and types of medicines (Western medicines vs. TCMs). Furthermore, the use of price indices addresses the limitations of traditional metrics such as DDD and median price ratios, enabling a more comprehensive inclusion of medicine types in the analysis.^18,23,41^

 This study has several limitations. First, because of underdeveloped electronic systems and the poor quality of handwritten records prior to and during the early phase of NEMP, procurement data from village clinics were unavailable before 2012. This restricted our ability to assess the short-term impact of NEMP around its initial implementation (2009–2012) at the most basic level of rural primary healthcare. Meanwhile, pharmaceutical records were available for only 6 observation points after the second-stage NEMP, which reduced the robustness of trend analysis. As the NEMP in China remains ongoing and continues to evolve, continuous observations are essential to monitor its sustained impact, particularly in deprived, remote and rural areas and during the more recent years (2018–2025).

 Second, we aggregated data from 7 THCs and 73 village clinics into two separate analytical units to facilitate analysis. This approach may have masked important variations in medicine supply, price and usage pattern across individual facilities. Investigation of these variations can have high policy relevance and implication in a rural, remote, and mountainous region, where populations, residential areas and socio-economic activities are widely dispersed. Third, the DPI calculation only considered essential medicines with complete records from 2012-2017, and therefore does not reflect the price trends of non-essential medicines over time.

 Finally, the use of monetary sales data rather than DDDs limits our ability to accurately assess the quantitative utilisation of medicine. While the DDD method has posed significant limitations in our context (only available for 23% of overall medicines), we advocate for development and application of more rigorous methodologies to measure medicine consumption in future research.

###  Implications

 This study, along with our previous research, contributes to providing a deeper understanding of the sustainable implementation and long-term impacts of NEMP in a rural, remote and economically underdeveloped county with limited health resources in China.^26^ Under the NEMP, a widening “inverted triangle” pattern has emerged across the three-tier healthcare system (county hospitals – THCs – village clinics), where higher-level facilities have benefited from greater access to medicines, smaller price increases and more substantial growth in pharmaceutical sales. In contrast, lower-tier facilities have faced significant challenges, including restricted medicine availability and utilisation, coupled with more pronounced price increases. These findings carry important policy implications for promoting equity and efficiency in medicine supply and utilisation under NEMP.

 The supply and usage prices of essential medicines in rural primary care system are influenced not only by top-down policy enforcement, but also by market dynamics within a complex adaptive system. Since reductions in the prices of individual medicines did not consistently translate into lower acquisition costs in clinical practice, pricing mechanisms under centralized tendering, procurement and distribution should also account for manufacturing and transportation costs, as well as ensuring reasonable profit margins for suppliers. Targeted support, such as logistical assistance and financial subsidies, should be further explored for medicine delivery in underserved, remote and rural areas.

 Price adjustments should also prioritise medicines experiencing declining supply (eg, medicines for metabolism, blood, dermatology, antiparasitic, nervous system, and sensory organs), as well as those with persistently rising prices (eg, Western medicines in village clinics). Optimising bidding and procurement policies, refining pricing mechanisms, establishing shortage warning systems, and enhancing administrative support can all help to improve the balance between manufacturer profits, patient affordability, and medicine quality.^18,23,42^

 From a broader perspective, the hierarchical healthcare system in rural China requires further refinement to address the growing disparities across different levels of facilities under NEMP. A core element of this system is the coordination of medicine categories and availability across different tiers of care.^43^ Secondary care facilities, compared with primary care facilities, typically stock and prescribe a broader range of Western, originator-brand, and non-essential medicines under fewer restrictions. These discrepancies in medicine availability can undermine continuity of care and erode patient trust in primary care, driving patients towards higher-level facilities for more comprehensive and higher-quality health services and medicines. The private sector, including individual pharmacies and private hospitals, also play a pivotal role in medicine supply, interacting with the availability, affordability and utilisation of medicines in public healthcare settings.^26^ These stakeholders should also be considered in the design and refinement of NEMP in the hierarchical healthcare system in rural areas.

 Equally important is the rational allocation of medical and public health responsibilities, alongside the enhancement of clinical service capacity in primary healthcare facilities, particularly in village clinics. One notable structural shift is the growing reliance on TCMs in these facilities, marked by a broader variety, higher volumes and more stable prices compared to Western medicines. Interestingly, this trend coincides with a decline in outpatient visits to village clinics. These concurrent trends may signal a long-term transformation in therapeutic approaches and operational capacity at the village level. While THCs function as intermediaries between primary and secondary care, the persistent challenges faced by village clinics demands greater attention in long-term policy-making. Strengthening their role as frontline health providers and gatekeepers, requires sustained investment in clinical training and the rational use of essential medicines. This must be supported by appropriate financial reimbursement mechanisms and incentive structures to encourage the delivery of quality clinical care and the prescription of essential medicines.^15,26^

 Lastly, further studies should focus on ongoing monitoring and evaluation of NEMP, especially in light of continuous policy refinements. In a complex adaptive system, top-down policy implementation must account for the disproportionate impacts across care levels, medicine types, and even individual facitilies.^26^ Investigating the relationships between changes in medicine variety, prices and usage, remains an essential yet underexplored area. Incorporating data from the private sector and examining the interplay between public and private actors, including procurement, pricing, stocking practices, and patient preferences, will provide more valuable insights into the evolving dynamics of medicine availability and affordability within China’s rural healthcare system.

## Conclusions

 The overall supply and utilisation of essential medicines in the Southwestern China’s rural primary care system remained relatively stable during the long-term implementation of NEMP, accompanied by modest but continuous price increases. Notable disparities and structural shifts emerged across different healthcare facility levels and medicine types. TCMs demonstrated more consistent improvements in availability and experienced fewer price increases compared to Western medicines. Village clinics in particular, faced more severe challenges, including a continuous decline in the variety of Western medicines available and marked price hikes. Future policy adaptions should seek to address the disproportionate long-term impacts of NEMP on different pharmaceuticals and on the most fundamental and resource-constrained levels of rural primary care.

## Ethical issues

 This study does not involve human participants or identifiable personal data and therefore does not require ethical approval, as it is based solely on secondary data sources.

## Conflicts of interest

 Authors declare that they have no conflicts of interest.

## Supplementary files


Supplementary file 1. Policy Implementation and Drug List.

Supplementary file 2. Description and Group Differences on Medicine Outcomes.

Supplementary file 3. Medicine Number and Sales by ATC System and TCM Classification.

Supplementary file 4. Medicine Price by ATC System and TCM Classification.

Supplementary file 5. Evidence Integration in Secondary Care Facilities.
