# Supplementary material for: Longitudinal Trends in Medicine Supply, Price and Utilisation in Primary Care Facilities in Rural Southwestern China Under National Essential Medicines Policy (2012-2017): Disparities Across Facilities and Medicines
Source: Int J Health Policy Manag. 2025 Nov 18;14:8991. doi: 10.34172/ijhpm.8991 (PMC12958208; doi:10.34172/ijhpm.8991)
Supplement: Supplementary file 1 — Policy Implementation and Drug List. [file ijhpm-14-8991-s001.pdf]

Article title: Longitudinal Trends in Medicine Supply, Price and Utilisation in Primary Care Facilities in Rural Southwestern China Under National Essential Medicines Policy (2012-2017): Disparities Across Facilities and Medicines

Journal name: International Journal of Health Policy and Management (IJHPM)

Authors' information: Zhaohua Huo[1¶], Xuechen Xiong[2,3¶], Ge Bai[4], Jianchao Quan[2], Allen TC Lee[1], Linda CW Lam[1], Li Luo[*4]

[1]Department of Psychiatry, Faculty of Medicine, The Chinese University of Hong Kong, Hong Kong SAR, China.

[2]School of Public Health, The University of Hong Kong, Hong Kong SAR, China.

[3]Department of Applied Social Sciences, The Hong Kong Polytechnic University, Hong Kong SAR, China.

[4]School of Public Health, Fudan University, Shanghai, China.

***Correspondence to:** Li Luo; Email: liluo@fudan.edu.cn

¶ Both authors contributed equally to this paper.

Citation: Huo Z, Xiong X, Bai G, et al. Longitudinal trends in medicine supply, price and utilisation in primary care facilities in rural southwestern China under National Essential Medicines Policy (2012-2017): disparities across facilities and medicines. Int J Health Policy Manag. 2025;14:8991. doi:10.34172/ijhpm.8991

Supplementary file 1. Policy Implementation and Drug List

Table S1: Implementation of National Essential Medicines Policy (NEMP) in the county level

Policy[a]	Pre-NEMP (before November 2010)	Stage 1 NEMP (November 2010 – October 2015)		Stage 2 NEMP (after November 2015)
	All healthcare providers[b]	Primary care providers[b]	County hospitals[b]	County hospitals[b]
Procurement and distribution	● Healthcare providers can procure medicines from different distributors at their discretion.	● **Centralized and province-wide tendering and selection** of pharmaceutical producers and distributors, based on price-volume-linked and bulk purchasing (since November 2010).<br>● Healthcare providers procure medicines through the provincial integrated platform at regulated prices (since November 2010).	● Healthcare providers can choose to procure medicines through the provincial platform or by direct contact with pharmaceutical producers and distributors (since November 2010).	● Healthcare providers are required to procure medicines through the provincial centralized platform as priority.
Clinical use	● Not limit	● Compulsory use of medicines from **National Essential Medicines List** and provincial supplementary list (since June 2010).<br>● **Township healthcare centre**s are allowed to use **unlisted drugs** within the limits of 10% (since May 2011) or 20% (since January 2015) of total drug use.	● Use of essential medicines should exceed 30% of total drug use (since January 2012).	● Use of essential medicines should exceed 50% of total drug use.
Pricing	● Healthcare providers sell out medicines at procurement costs plus 15% profit margin.	● **Zero-markup policy**: retail prices of medicines equal to procurement costs (since November 2010).	● Keep 15% of profit margin when selling out medicines.	● Zero-markup policy for all drugs (except for Chinese herbal medicine).
Financial compensation for zero-mark-up policy	/	● Government subsidies for healthcare providers, based on service population, area and performance (since January 2012)	/	● Price adjustment on medical services and government subsidies for facilities.

a. Policies under the National Essential Medicines Scheme were retrieved from official documents at national, provincial and county levels.
b. The three-tier healthcare system in the selected county includes 73 village clinics (first-tier, primary care), 7 township healthcare centres (second-tier, primary care) and 3 county hospitals (third-tier, secondary care).

Table S2: Full lists of medicines by policy classifications

Type	Essential drug list	Generic name for western medicine (Pinyin for Traditional Chinese Medicine)
Western medicine	Essential medicine	**A**cetagastrodin; Acetylsalicylic acid; Aciclovir; Adenosine; Aescinate; Albendazole; Alfacalcidol; Allopurinol; Amantadine; Ambroxol; Amikacin; Aminomethylbenzoic acid; Aminophenazone, combinations excl. psycholeptics; Aminophylline; Amlodipine; Amoxicillin; Amoxicillin and Clavulanate Potassium; Ampicillin; Anisodamine; Ascorbic acid (vit C); Atenolol; Atropine; Azathioprine; Azithromycin; **B**elladonna total alkaloids; Benazepril; Benzylpenicillin; Berberine; Bergenini Compositae; Betahistine; Bismuth Potassium Citrate; Bismuth, Magnesium and Sodium Bicarbonate; Butenafine; **C**alamine; Calcium Carbonate and Vitamin D3; Calcium chloride; Calcium folinate; Calcium gluconate; Captopril; Carbamazepine; Carbamide; Cefaclor; Cefalexin; Cefathiamidine; Cefazolin; Cefixime; Cefotaxime; Cefpiramide; Cefradine; Ceftazidime; Ceftriaxone; Cefuroxime; Chloramphenicol; Chlorphenamine; Chlorpromazine; Chlortetracycline; Chymotrypsin; Ciprofloxacin; Citicoline; Clindamycin; Clotrimazole; Codeine, combinations excl. psycholeptics; Coenzyme A; Colchicine; Colloidal Bismuth Pectin; Combinations of barbiturates; Compound Aluminium Hydroxide; Compound Amino Acid 18AA; Compound Liquorice; Compound Sodium Chloride; Cyanocobalamin; Cyproheptadine; **D**eslanoside; Dexamethasone; Dextran; Diclofenac; Difenidol; Digoxin; Diphenhydramine; Dobutamine; Domperidone; Dopamine; **E**nalapril; Epinephrine; Ergocalciferol; Erythromycin; Erythromycin, combinations; Etamsylate; **F**amotidine; Fat emulsions; Felodipine; Fluconazole; Flunarizine; Fluocinolone acetonide; Folic acid; Fosfomycin; Furazolidone; Furosemide; **G**entamicin; Glibenclamide; Gliclazide; Glipizide; Glucose; Glucurolactone; Glutathione; Glycerine Enema or Sorbitol Enema; Glyceryl trinitrate; **H**eparin; Hydrobenzole; Hydrochlorothiazide; Hydrocortisone; **I**buprofen; Ichtasol; Indapamide; Indometacin; Inosine; Insulin (human); Irbesartan; Isoniazid; Isoprenaline; **L**actasin; Levofloxacin; Lidocaine; Lincomycin; Live Combined Bacillus Subtilis and Enterococcus Faecium; Live Combined Bifidobacterrium,Lactobacillus and Enterococcus; Lobeline; Loratadine; Lovastatin; **M**agnesium sulfate; Mannitol; Medroxyprogesterone; Metamizole sodium; Metformin; Methylthioninium chloride; Metoclopramide; Metoprolol; Metronidazole; Miconazole; Mifepristone; Misoprostol; Multivitamins and other minerals, incl. combinations; **N**aloxone; Neostigmine; Nifedipine; Nikethamide; Nimodipine; Nitrendipine; Nitrofurantoin; Norepinephrine; Norfloxacin; Nystatin, combinations; **O**floxacin; Omeprazole; Ordinary salt combinations; Oryzanol; Oxytocin; **P**aracetamol, combinations excl. psycholeptics; Pentoxyverine; Phenolphthalein; Phenoxymethylpenicillin; Phenytoin; Phytomenadione; Pilocarpine; Piperacillin; Piracetam; Potassium chloride; Potassium Dehydroandrograpolide Succinate; Pralidoxime; Prednisone; Procaine; Progesterone; Promethazine; Propranolol; Pyridoxine (vit B6); **R**anitidine; Repaglinide; Reserpine, combinations; Ribavirin; Riboflavin (vit B2); Rice Bran Sterol; Rifampicin; Roxithromycin; **S**albutamol; Salicylic acid; Silver sulfadiazine; Simvastatin; Smectite; Sodium bicarbonate; Sodium chloride; Sodium Lactate Ringer's; Spironolactone; Sterile Water for Injection; Steroidal saponins; Sucralfate; Sulfamethoxazole; **T**erazosin; Terbutaline; Tetracycline; Theophylline; Thiamine (vit B1); Thrombin; Thyroid gland preparations; Timolol; Tinidazole; Tobramycin; Tranexamic acid; Tretinoin; Triamcinolone; Tripterysium Glycosides; Tropicamide, combinations; **U**rsodeoxycholic acid; **V**alproic acid; Valsartan; Verapamil; Vitamin U,Belladonna and Aluminium Capsules; **W**arfarin;
	Non-essential medicine	Beclometasone; Cerebroprotein; Cimetidine; Compound Ammonium Glycyrrhetate; Cromoglicic acid; Cydiodine Buccal; Cytochrome C; Dexamethasone, combinations; Fenbufen; Ferulate; Gastrodin; Haemocoagulase Atrox; Iron, vitamin B12 and folic acid; Kitasamycin; Lactic acid producing organisms; Mupirocin; Naphazoline, combinations; Oxytetracycline;

		Zinc gluconate
Chinese Traditional Medicine	Essential medicine	(Chinese Pinyin) **A**n Gong Niu Huang Wan; An Wei Yang Jiao Nang; **B**a Zhen Yi Mu Wan (Jiao Nang); Bai Ji Ke Li (Fen/Tang Jiang); Ban Lan Gen Ke Li; Ban Lan Qing Re Ke Li; Bao Fu Kang Shuan; Bao Ji Wan (Kou Fu Ye); Bao He Chong Ji; Bao He Wan; Bi Yan Kang Pian; Bing Peng San; Bu Zhong Yi Qi Wan (Ke Li); **C**an Fu Zhu She Ye; Can Ling Bai Shu San (Wan/Ke Li); Can Ling Jian Pi Wei Ke Li; Can Mai Zhu She Ye; Can Song Yang Xin Jiao Nang; Chai Hu Zhu She Ye; Chen Xiang Lu Bai Lu Pian; Chuan Xiong Cha Diao Ke Li; Chuan Xiong Cha Diao Pian; Ci Wu Jia Nao Ling Ye; **D**a Huo Luo Wan (Jiao Nang); Dan Can Zhu She Ye (Dong Gan /Fen Zhen); Dan Deng Tong Nao Jiao Nang (Ruan Jiao Nang); Dan Zhi Xiao Yao Wan; Deng Zhan Hua Su Pian; Deng Zhan Hua Su Zhu She Ye; Deng Zhan Xi Xin Ke Li; Du Zhong Jiang Ya Pian; **E**r Long Zuo Ci Wan; **F**ang Feng Tong Sheng Ke Li; Feng Han Gan Mao Ke Li; Feng Re Gan Mao Ke Li; Feng Shi Gu Tong Jiao Nang; Feng Shi Ye; Fu Fang Dan Can Pian (Ke Li/Jiao Nang/Di Wan); Fu Fang Feng Shi Ning Jiao Nang (Pian); Fu Fang Long Xue Jie Jiao Nang; Fu Fang Nan Ban Lan Gen Pian; Fu Fang Nan Xing Zhi Tong Gao; Fu Fang Sha Ji Zi You Shuan; Fu Fang Xue Shuan Tong Jiao Nang (Pian); Fu Ke Qian Jin Pian (Jiao Nang); Fu Ke Shi Wei Pian; Fu Yan Kang Pian; Fu Yan Xiao Jiao Nang; Fu Zi Li Zhong Pian / Wan; **G**an Mao Qing Re Ke Li (Jiao Nang); Gan Mao Shu Feng Pian (Ke Li/Wan); Gan Mao Xiao Yan Pian; Ge Jie Ding Chuan Jiao Nang / Wan; Geng Nian An Pian (Jiao Nang); Gong Xue Ning Jiao Nang; Gong Yan Ping Pian; Gou Pi Gao; Gu Tong Ling Ding; Guan Xin Ning Zhu She Ye; Gui Pi Wan (He Ji); Gui Zhi Fu Ling Wan (Jiao Nang); **H**an Chuan Zu Pa Ke Li; Heng Gu Gu Shang Yu He Ji; Hong Hua Huang Se Su; Hu Gan Pian (Ke Li/Jiao Nang); Hu Li San Jiao Nang; Hua Hong Pian (Ke Li/Jiao Nang); Hua Tan Li Yan Huang Shi Xiang Sheng Wan; Hua Tuo Zai Zao Wan; Huang Lian Shang Qing Wan (Ke Li/Jiao Nang/Pian); Huang Qi Zhu She Ye; Huang Teng Su Pian (Fen San Pian/Jiao Nang/Ruan Jiao Nang); Huo Dan Wan; Huo Xiang Zheng Qi Shui (Kou Fu Ye/Ruan Jiao Nang， Ke Li); Huo Xue Zhi Tong San (Jiao Nang); Huo Xue Zhi Tong San; **J**i De Sheng She Yao Pian; Ji Zhi Tang Jiang (Ke Li); Jian Er Xiao Shi Kou Fu Ye; Jian Pi Sheng Xue Ke Li; Jin Kui Shen Qi Wan (Pian); Jin Qian Cao Ke Li; Jing Fu Kang Ke Li; Jing Shu Ke Li; Jiu Wei Qiang Huo Ke Li; Ju Hong Wan (Ke Li/Jiao Nang/Pian); **K**ang Bing Du Kou Fu Ye (Ke Li); Kang Fu Xin Ye; Kang Gan Ling Pian; Kang Gong Yan Pian /Jiao Nang; Kou Yan Qing Ke Li; Kun Ming Shan Hai Tang Pian; **L**i Dan Zhi Tong Jiao Nang /Pian; Lian Hua Qing Wen Jiao Nang (Ke Li); Lian Qiao Bai Du Pian; Liu Wei Di Huang Wan (Ke Li/Jiao Nang); Long Bi Shu Jiao Nang; Long Qing Jiao Nang; Long Xue Jie Jiao Nang; **M**a Ren Run Chang Wan (Ruan Jiao Nang); Ma Ying Long Zhi Chuang Gao; Ming Mu Di Huang Wan; Mo Luo Ning Zhu She Ye; Mo Xue Kang Jiao Nang; **N**ao An Di Wan; Nao Xin Tong Wan (Jiao Nang/Pian); Niu Huang Jie Du Wan (Jiao Nang/Ruan Jiao Nang/Pian); Niu Huang Shang Qing Wan (Jiao Nang/Pian); **P**ai Shi Ke Li; Pen Yan Jing Ke Li (Jiao Nang); Pu Le An Jiao Nang (Pian); **Q**i Ju Di Huang Wan (Jiao Nang/Pian); Qi Ye Shen An Pian; Qi Zhi Wei Tong Ke Li (Pian); Qiang Li Pi Pa Lu; Qing Fei Hua Tan Wan; Qing Fei Yi Huo Pian; Qing Kai Ling Ke Li (Jiao Nang/Pian/Zhu She Ye); Qing Xuan Zhi Ke Ke Li; **R**en Can Zai Zao Wan; Ru Kuai Xiao Ke Li; Ru Pi Xiao Ke Li (Jiao Nang/Pian); Run Fei Gao; Run Zao Zhi Yang Jiao Nang; **S**an Jiu Wei Tai Ke Li (Jiao Nang); San Qi Jiao Nang; Se Chang Zhi Xie San; Shang Ke Jie Gu Pian; Shao Fu Zhu Yu Jiao Nang; She Dan Chen Pi Kou Fu Ye; She Dan Chuan Bei Ye; She Xiang Bao Xin Wan; Shen Qi Jiang Tang Pian / Jiao Nang / Ke Li; Sheng Mo Yin (Ke Li/Jiao Nang/Zhu She Ye); Sheng Xue Bao Ke Li; Shi Di Shui; Shu Feng Jie Du Jiao Nang; Shu Gan Ke Li; Shu Jin Huo Xue Wan (Pian， Jiao Nang); Shu Mi Tong Jiao Nang; Shu Xin Tong Mo Jiao Nang; Shuang Huang Lian He Ji (Kou Fu Ye/Ke Li/Jiao Nang/Pian); Shuang Huang Lian Zhu She Ye; Si Shen Wan (Pian); Song Ling Xue Mai Kang Jiao Nang; Su Xiao Jiu Xin Wan; Suo Quan Jiao Nang; Tian Ma Xing Nao Jiao Nang; **T**ian Qi Hua Ye Ke Li; Tian Wang Bu Xin Wan (Pian); Tian

		Zi Hong Nü Jin Jiao Nang; Tong Bian Ling Jiao Nang; Tong Luo Qu Tong Gao; Tong Qiao Bi Yan Pian; Tong Shu Jiao Nang; Tong Xin Luo Jiao Nang; Tong Xuan Li Fei Wan (Ke Li/Jiao Nang/Pian); **W**ang Bi Ke Li (Jiao Nang/Pian); Wei Su Ke Li; Wen Xin Ke Li; Wu Di Dan Jiao Nang; Wu Ji Bai Feng Wan (Jiao Nang/Pian); **X**ian Ling Gu Bao Jiao Nang (Pian); Xiang Guo Jian Xiao Pian; Xiang Ju Jiao Nang (Pian); Xiang Sha Ping Wei Wan (Ke Li); Xiao Chai Hu Ke Li; Xiao Er Hua Du San (Jiao Nang); Xiao Er Hua Tan Zhi Ke Ke Li; Xiao Er Ke Chuan Ling Ke Li; Xiao Er Re Su Qing Kou Fu Ye (Ke Li); Xiao Er Xiao Ji Zhi Ke Kou Fu Ye; Xiao Er Xie Su Ting Ke Li; Xiao Er Yan Bian Ke Li; Xiao Ke Wan; Xiao Yan Li Dan Pian (Ke Li/Jiao Nang); Xiao Yan Pian; Xiao Yao Wan (Ke Li); Xin Fu Fang Da Qing Ye Pian; Xin Ke Shu Jiao Nang (Pian); Xin Qin Ke Li; Xing Pi Yang Er Ke Li; Xiong Dan Shu Gan Li Dan Jiao Nang; Xiong Dan Yan Yao Shui; Xue Sai Tong Jiao Nang (Pian); Xue Sai Tong Zhu She Ye (Dong Gan); Xue Zhi Kang Pian (Jiao Nang); Xue Fu Zhu Yu Jiao Nang; Yang Xue Qing Nao Wan (Ke Li); **Y**ang Yin Qing Fei Ke Li; Yao Bi Tong Jiao Nang; Ye Xia Zhu Jiao Nang; Yi Mo Kang Pian; Yi Mu Cao Gao (Ke Li/Jiao Nang/Pian); Yi Qing Ke Li; Yi Xin Shu Ke Li (Jiao Nang/Pian); Yin Huang Kou Fu Ye (Ke Li/Jiao Nang/Pian); Yin Qiao Jie Du Wan (Ke Li/Jiao Nang/Ruan Jiao Nang/Pian); Yin Xing Ye Jiao Nang (Pian/Di Wan); Yin Zhi Huang Kou Fu Ye (Ke Li); Yu Ping Feng Ke Li; Yuan Hu Zhi Tong Pian (Ke Li/Jiao Nang/Di Wan); Yun Nan Bai Yao (Jiao Nang/Ke Li/Gao/Ding/Qi Wu Ji); Yun Nan Hong Yao Jiao Nang; Yun Tong Ding Jiao Nang; **Z**ao Ren An Shen Ke Li (Jiao Nang); Zhen Xiong Dan Wan; Zheng Tian Wan (Jiao Nang); Zheng Wei Jiao Nang / Pian; Zhi Bai Di Huang Wan; Zhui Feng Tou Gu Wan; Zu Shi Ma Zhu She Ye; Zu Ka Mu Ke Li<br>(Chinese name) 安宫牛黄丸; 安胃疡胶囊; 八珍益母丸（胶囊）; 白芨颗粒（粉、糖浆）; 板蓝根颗粒; 板蓝清热颗粒; 保妇康栓; 保济丸（口服液）; 保和冲剂; 保和丸; 鼻炎康片; 冰硼散; 补中益气丸（颗粒）; 参附注射液; 参苓白术散（丸、颗粒）; 参苓健脾胃颗粒; 参麦注射液; 参松养心胶囊; 柴胡注射液; 陈香露白露片; 川芎茶调颗粒; 川芎茶调片; 刺五加脑灵液; 大活络丸（胶囊）; 丹参注射液（冻干/粉针）; 丹灯通脑胶囊（软胶囊）; 丹栀逍遥丸; 灯盏花素片; 灯盏花素注射液; 灯盏细辛颗粒; 杜仲降压片; 耳聋左慈丸; 防风通圣颗粒; 风寒感冒颗粒; 风热感冒颗粒; 风湿骨痛胶囊; 风湿液; 复方丹参片（颗粒、胶囊、滴丸）; 复方风湿宁胶囊（片）; 复方龙血竭胶囊; 复方南板蓝根片; 复方南星止痛膏; 复方沙棘籽油栓; 复方血栓通胶囊（片）; 妇科千金片（胶囊）; 妇科十味片; 妇炎康片; 妇炎消胶囊; 附子理中片/丸; 感冒清热颗粒（胶囊）; 感冒疏风片（颗粒、丸）; 感冒消炎片; 蛤蚧定喘胶囊/丸; 更年安片（胶囊）; 宫血宁胶囊; 宫炎平片; 狗皮膏; 骨痛灵酊; 冠心宁注射液; 归脾丸（合剂）; 桂枝茯苓丸（胶囊）; 寒喘祖帕颗粒; 恒古骨伤愈合剂; 红花黄色素; 护肝片（颗粒、胶囊）; 虎力散胶囊; 花红片（颗粒、胶囊）; 化痰利咽黄氏响声丸; 华佗再造丸; 黄连上清丸（颗粒、胶囊、片）; 黄芪注射液; 黄藤素片（分散片、胶囊、软胶囊）; 藿胆丸; 藿香正气水（口服液、软胶囊，颗粒）; 活血止痛散（胶囊）; 活血止痛散; 季德胜蛇药片; 急支糖浆（颗粒）; 健儿消食口服液; 健脾生血颗粒; 金匮肾气丸（片）; 金钱草颗粒; 颈复康颗粒; 颈舒颗粒; 九味羌活颗粒; 橘红丸（颗粒、胶囊、片）; 抗病毒口服液（颗粒）; 康复新液; 抗感灵片; 抗宫炎片/胶囊; 口炎清颗粒; 昆明山海棠片; 利胆止痛胶囊/片; 莲花清瘟胶囊（颗粒）; 连翘败毒片; 六味地黄丸（颗粒、胶囊）; 癃闭舒胶囊; 癃清胶囊; 龙血竭胶囊; 麻仁润肠丸（软胶囊）; 马应龙痔疮膏; 明目地黄丸; 脉络宁注射液; 脉血康胶囊; 脑安滴丸; 脑心通丸（胶囊、片）; 牛黄解毒丸（胶囊、软胶囊、片）; 牛黄上清丸（胶囊、片）; 排石颗粒; 盆炎净颗粒（胶囊）; 普乐安胶囊（片）; 杞菊地黄丸（胶囊、片）; 七叶神安片; 气滞胃痛颗粒（片）; 强力枇杷露; 清肺化痰丸; 清肺抑火片; 清开灵颗粒（胶囊、片、注射液）; 清宣止咳颗粒; 人参再造丸; 乳块消颗粒; 乳癖消颗粒（胶囊、片）; 润肺膏; 润燥止痒胶囊; 三九胃泰颗粒（胶囊）; 三七胶囊; 涩肠止泻散; 伤科接骨片; 少腹逐瘀胶囊; 蛇胆陈皮口服液; 蛇胆川贝液; 麝香保心丸; 参芪降糖片/胶囊/颗粒; 生脉饮（颗粒、胶囊、注射液）; 生血宝颗粒; 十滴水; 疏风解毒胶囊; 舒肝颗粒; 舒筋活血丸（片，胶囊）; 舒泌通胶囊; 舒心通脉胶囊; 双黄连合剂（口服液、颗粒、胶囊、片）; 双黄莲注射液; 四神丸（片）; 松龄血脉康胶

		囊; 速效救心丸; 缩泉胶囊; 天麻醒脑胶囊; 田七花叶颗粒; 天王补心丸（片）; 天紫红女金胶囊; 通便灵胶囊; 通络祛痛膏; 通窍鼻炎片; 痛舒胶囊; 通心络胶囊; 通宣理肺丸（颗粒、胶囊、片）; 尪痹颗粒（胶囊、片）; 胃苏颗粒; 稳心颗粒; 无敌丹胶囊; 乌鸡白凤丸（胶囊、片）; 仙灵骨葆胶囊（片）; 香果健消片; 香菊胶囊（片）; 香砂平胃丸（颗粒）; 小柴胡颗粒; 小儿化毒散（胶囊）; 小儿化痰止咳颗粒; 小儿咳喘灵颗粒; 小儿热速清口服液（颗粒）; 小儿消积止咳口服液; 小儿泻速停颗粒; 小儿咽扁颗粒; 消渴丸; 消炎利胆片（颗粒、胶囊）; 消炎片; 逍遥丸（颗粒）; 新复方大青叶片; 心可舒胶囊（片）; 辛芩颗粒; 醒脾养儿颗粒; 熊胆舒肝利胆胶囊; 熊胆眼药水; 血塞通胶囊（片）; 血塞通注射液（冻干）; 血脂康片（胶囊）; 血府逐瘀胶囊; 养血清脑丸（颗粒）; 养阴清肺颗粒; 腰痹通胶囊; 叶下珠胶囊; 益脉康片; 益母草膏（颗粒、胶囊、片）; 一清颗粒; 益心舒颗粒（胶囊、片）; 银黄口服液（颗粒、胶囊、片）; 银翘解毒丸（颗粒、胶囊、软胶囊、片）; 银杏叶胶囊（片、滴丸）; 茵栀黄口服液（颗粒）; 玉屏风颗粒; 元胡止痛片（颗粒、胶囊、滴丸）; 云南白药(胶囊、颗粒、膏、酊、气雾剂); 云南红药胶囊; 晕痛定胶囊; 枣仁安神颗粒（胶囊）; 珍熊胆丸; 正天丸（胶囊）; 正胃胶囊/片; 知柏地黄丸; 追风透骨丸; 祖师麻注射液; 祖卡木颗粒
	Non-essential medicine	(Pinyin) An Shen Bu Nao Ye; Chang Yan Ning Jiao Nang (Pian); Chuan Bei Pi Pa Gao; Chuan Bei Qing Fei Tang Jiang; Feng Liao Chang Wei Kang Ke Li; Fu Fang A Jiao Jiang; Gan Mao Ling (Ke Li/Chong Ji/Jiao Nang/Pian); Jian Er Qing Jie Ye; Jian Wei Xiao Shi Pian; Jie Er Yin Xi Ye; Jin Gang Teng Jiao Nang; Luo Han Guo Zhi Ke Tang Jiang; Mei Bao Shi Run Shao Shang Gao; Nao Xin Shu Kou Fu Ye; Qing Hou Li Yan Ke Li; San Jin Pian; Si Mo Tang Kou Fu Ye; Tan Ke Jing San (Pian); Xi Gua Shuang Pian; Xi Xin Nao Zhu She Ye; Xiao Er Chang Wei Kang Ke Li; Xiao Er Fei Re Ke Chuan Kou Fu Ye; Xiao Er Qing Fei Hua Tan Ke Li; Xiao Zhong Zhi Tong Ding; Zhi Ke Wan; Zu Shi Ma Guan Jie Zhi Tong (Chinese name) 安神补脑液; 肠炎宁胶囊（片）; 川贝枇杷膏; 川贝清肺糖浆; 枫蓼肠胃康颗粒; 复方阿胶浆; 感冒灵（颗粒、冲剂、胶囊、片）; 健儿清解液; 健胃消食片; 洁尔阴洗液; 金刚藤胶囊; 罗汉果止咳糖浆; 美宝湿润烧伤膏; 脑心舒口服液; 清喉利咽颗粒; 三金片; 四磨汤口服液; 痰咳净散（片）; 西瓜霜片; 细辛脑注射液; 小儿肠胃康颗粒; 小儿肺热咳喘口服液; 小儿清肺化痰颗粒; 消肿止痛酊; 止咳丸; 祖师麻关节止痛

Table S3: Full lists of drugs by functional classification

ATC Classification	Generic name (western medicine)
Alimentary Tract and Metabolism	Acetylsalicylic acid; Alfacalcidol; Ascorbic acid (vit C); Atropine; Beclometasone; Belladonna total alkaloids; Berberine; Bismuth Potassium Citrate; Bismuth, Magnesium and Sodium Bicarbonate; Calcium Carbonate and Vitamin D3; Calcium chloride; Calcium gluconate; Chlortetracycline; Cimetidine; Clotrimazole; Colloidal Bismuth Pectin; Compound Aluminium Hydroxide; Compound Amino Acid 18AA; Compound Ammonium Glycyrrhetate; Compound Sodium Chloride; Cromoglicic acid; Cydiodine Buccal; Dexamethasone; Domperidone; Epinephrine; Ergocalciferol; Famotidine; Glibenclamide; Gliclazide; Glipizide; Glucurolactone; Glycerine Enema or Sorbitol Enema; Hydrocortisone; Insulin (human); Lactasin; Lactic acid producing organisms; Live Combined Bacillus Subtilis and Enterococcus Faecium; Live Combined Bifidobacterrium,Lactobacillus and Enterococcus; Magnesium sulfate; Mannitol; Metformin; Metoclopramide; Metronidazole; Miconazole; Misoprostol; Multivitamins and other minerals, incl. combinations; Omeprazole; Ordinary salt combinations; Phenolphthalein; Potassium chloride; Prednisone; Pyridoxine (vit B6); Ranitidine; Repaglinide; Riboflavin (vit B2); Rice Bran Sterol; Smectite; Sodium chloride; Sodium Lactate Ringer's; Sucralfate; Tetracycline; Thiamine (vit B1); Triamcinolone; Tripterysium Glycosides; Ursodeoxycholic acid; Vitamin U,Belladonna and Aluminium Capsules; Zinc gluconate;
Blood and Blood Forming Organs	Acetylsalicylic acid; Aescinate; Aminomethylbenzoic acid; Calcium chloride; Carbamide; Chymotrypsin; Coenzyme A; Compound Amino Acid 18AA; Compound Sodium Chloride; Cyanocobalamin; Dextran; Epinephrine; Etamsylate; Fat emulsions; Ferulate; Folic acid; Glucose; Haemocoagulase Atrox; Heparin; Iron, vitamin B12 and folic acid; Magnesium sulfate; Mannitol; Phytomenadione; Potassium chloride; Sodium bicarbonate; Sodium chloride; Sodium Lactate Ringer's; Thrombin; Tranexamic acid; Warfarin
Cardiovascular System	Adenosine; Amlodipine; Atenolol; Benazepril; Captopril; Deslanoside; Dexamethasone; Digoxin; Dobutamine; Dopamine; Enalapril; Epinephrine; Felodipine; Ferulate; Fluocinolone acetonide; Furosemide; Glyceryl trinitrate; Heparin; Hong Hua Huang Se Su ; Hydrochlorothiazide; Hydrocortisone; Ibuprofen; Indapamide; Indometacin; Irbesartan; Isoprenaline; Lidocaine; Lovastatin; Metoprolol; Nifedipine; Nimodipine; Nitrendipine; Norepinephrine; Procaine; Propranolol; Reserpine, combinations; Simvastatin; Spironolactone; Steroidal saponins; Timolol; Valsartan; Verapamil
Dermatological	Aciclovir; Amikacin; Beclometasone; Butenafine; Calcium gluconate; Carbamide; Chloramphenicol; Chlortetracycline; Clindamycin; Clotrimazole; Cromoglicic acid; Dexamethasone; Diclofenac; Diphenhydramine; Erythromycin; Erythromycin, combinations; Fluconazole; Gentamicin; Hydrocortisone; Ichtasol; Inosine; Lidocaine; Magnesium sulfate; Metronidazole; Miconazole; Mupirocin; Oxytetracycline; Salicylic acid; Silver sulfadiazine; Tetracycline; Tretinoin; Triamcinolone
Genito Urinary and Sex Hormones	Ascorbic acid (vit C); Calcium chloride; Chloramphenicol; Clindamycin; Clotrimazole; Furazolidone; Ibuprofen; Inosine; Metronidazole; Miconazole; Mifepristone; Misoprostol; Nystatin, combinations; Oxytetracycline; Progesterone; Terazosin
Systemic Hormonal	Dexamethasone; Hydrocortisone; Oxytocin; Prednisone; Thyroid gland preparations; Triamcinolone
Anti-infective For Systemic Use	Aciclovir; Amikacin; Amoxicillin; Amoxicillin and Clavulanate Potassium; Ampicillin; Benzylpenicillin; Cefaclor; Cefalexin; Cefathiamidine; Cefazolin; Cefixime; Cefotaxime; Cefpiramide; Cefradine; Ceftazidime; Ceftriaxone; Cefuroxime; Chloramphenicol; Chlortetracycline; Ciprofloxacin; Clindamycin; Compound Ammonium Glycyrrhetate; Erythromycin; Fluconazole; Fosfomycin; Gentamicin; Isoniazid; Kitasamycin; Levofloxacin; Lincomycin; Metronidazole; Miconazole; Nitrofurantoin; Norfloxacin; Ofloxacin; Oxytetracycline; Phenoxymethylpenicillin; Piperacillin; Ribavirin; Rifampicin; Roxithromycin; Sulfamethoxazole; Tetracycline; Tinidazole; Tobramycin; Tripterysium Glycosides
Antineoplastic and	Azathioprine; Medroxyprogesterone; Tretinoin

Immunomodulating	
Musculo-Skeletal System	Allopurinol; Amantadine; Colchicine; Diclofenac; Fenbufen; Glucurolactone; Ibuprofen; Indometacin; Tripterysium Glycosides
Nervous System	Acetagastrodin; Acetylsalicylic acid; Aescinate; Aminophenazone, combinations excl. psycholeptics; Anisodamine; Betahistine; Carbamazepine; Cerebroprotein; Chlorpromazine; Citicoline; Codeine, combinations excl. psycholeptics; Combinations of barbiturates; Difenidol; Flunarizine; Gastrodin; Lidocaine; Metamizole sodium; Neostigmine; Oryzanol; Paracetamol, combinations excl. psycholeptics; Phenytoin; Pilocarpine; Piracetam; Procaine; Valproic acid
Antiparasitic	Albendazole; Metronidazole; Tinidazole
Respiratory System	Ambroxol; Aminophylline; Amoxicillin and Clavulanate Potassium; Beclometasone; Bergenini Compositae; Chlorphenamine; Compound Liquorice; Cromoglicic acid; Cyproheptadine; Cytochrome C; Dexamethasone; Dexamethasone, combinations; Diphenhydramine; Epinephrine; Isoprenaline; Lidocaine; Lobeline; Loratadine; Mupirocin; Nikethamide; Pentoxyverine; Potassium Dehydroandrographolide Succinate; Promethazine; Salbutamol; Terbutaline; Theophylline; Triamcinolone
Sensory Organs	Aciclovir; Amikacin; Ampicillin; Ascorbic acid (vit C); Atropine; Azithromycin; Chloramphenicol; Chlortetracycline; Chymotrypsin; Ciprofloxacin; Cromoglicic acid; Dexamethasone; Diclofenac; Epinephrine; Erythromycin; Fluocinolone acetonide; Gentamicin; Heparin; Hydrobenzole; Hydrocortisone; Indometacin; Inosine; Levofloxacin; Lidocaine; Miconazole; Naphazoline, combinations; Neostigmine; Norfloxacin; Ofloxacin; Oxytetracycline; Pilocarpine; Procaine; Salicylic acid; Tetracycline; Timolol; Tobramycin; Triamcinolone; Tropicamide, combinations
TCM classification	TCM name
Internal medicine	(Chinese Pinyin) An Gong Niu Huang Wan; An Shen Bu Nao Ye; An Wei Yang Jiao Nang; Bai Ji Ke Li (Fen, Tang Jiang); Ban Lan Gen Ke Li; Ban Lan Qing Re Ke Li; Bao Ji Wan (Kou Fu Ye); Bao He Chong Ji; Bao He Wan; Bu Zhong Yi Qi Wan (Ke Li); Can Fu Zhu She Ye; Can Ling Bai Shu San (Wan, Ke Li); Can Ling Jian Pi Wei Ke Li; Can Mai Zhu She Ye; Can Song Yang Xin Jiao Nang; Chai Hu Zhu She Ye; Chang Yan Ning Jiao Nang (Pian); Chen Xiang Lu Bai Lu Pian; Chuan Bei Pi Pa Gao; Chuan Bei Qing Fei Tang Jiang; Chuan Xiong Cha Diao Ke Li; Chuan Xiong Cha Diao Pian; Ci Wu Jia Nao Ling Ye; Da Huo Luo Wan (Jiao Nang); Dan Can Zhu She Ye (Dong Gan /Fen Zhen); Dan Deng Tong Nao Jiao Nang (Ruan Jiao Nang); Dan Zhi Xiao Yao Wan; Deng Zhan Hua Su Pian; Deng Zhan Hua Su Zhu She Ye; Deng Zhan Xi Xin Ke Li; Du Zhong Jiang Ya Pian; Fang Feng Tong Sheng Ke Li; Feng Han Gan Mao Ke Li; Feng Liao Chang Wei Kang Ke Li; Feng Re Gan Mao Ke Li; Feng Shi Gu Tong Jiao Nang; Feng Shi Ye; Fu Fang A Jiao Jiang; Fu Fang Dan Can Pian (Ke Li, Jiao Nang, Di Wan); Fu Fang Feng Shi Ning Jiao Nang (Pian); Fu Fang Long Xue Jie Jiao Nang; Fu Fang Nan Ban Lan Gen Pian; Fu Zi Li Zhong Pian / Wan; Gan Mao Ling (Ke Li/Chong Ji/Jiao Nang/Pian); Gan Mao Qing Re Ke Li (Jiao Nang); Gan Mao Shu Feng Pian (Ke Li, Wan); Gan Mao Xiao Yan Pian; Ge Jie Ding Chuan Jiao Nang / Wan; Guan Xin Ning Zhu She Ye; Gui Pi Wan (He Ji); Han Chuan Zu Pa Ke Li; Hong Hua Huang Se Su; Hu Gan Pian (Ke Li, Jiao Nang); Hu Li San Jiao Nang; Hua Tan Li Yan Huang Shi Xiang Sheng Wan; Hua Tuo Zai Zao Wan; Huang Lian Shang Qing Wan (Ke Li, Jiao Nang, Pian); Huang Qi Zhu She Ye; Huang Teng Su Pian (Fen San Pian, Jiao Nang, Ruan Jiao Nang); Huo Xiang Zheng Qi Shui (Kou Fu Ye, Ruan Jiao Nang， Ke Li); Ji Zhi Tang Jiang (Ke Li); Jian Er Qing Jie Ye; Jian Er Xiao Shi Kou Fu Ye; Jian Pi Sheng Xue Ke Li; Jian Wei Xiao Shi Pian; Jin Kui Shen Qi Wan (Pian); Jiu Wei Qiang Huo Ke Li; Ju Hong Wan (Ke Li, Jiao Nang, Pian); Kang Bing Du Kou Fu Ye (Ke Li); Kang Gan Ling Pian; Kun Ming Shan Hai Tang Pian; Li Dan Zhi Tong Jiao Nang /Pian; Lian Hua Qing Wen Jiao Nang (Ke Li); Liu Wei Di Huang Wan (Ke Li, Jiao Nang); Long Bi Shu Jiao Nang; Long Qing Jiao Nang; Luo Han Guo Zhi Ke Tang Jiang; Ma Ren Run Chang Wan (Ruan Jiao Nang); Mo Luo Ning Zhu She Ye; Mo Xue Kang Jiao Nang; Nao An Di Wan; Nao Xin Shu Kou Fu Ye; Nao Xin Tong Wan (Jiao Nang, Pian); Niu Huang Jie Du Wan (Jiao Nang, Ruan Jiao Nang, Pian); Niu Huang Shang Qing Wan

	(Jiao Nang, Pian); Pu Le An Jiao Nang (Pian); Qi Ju Di Huang Wan (Jiao Nang, Pian); Qi Ye Shen An Pian; Qi Zhi Wei Tong Ke Li (Pian); Qiang Li Pi Pa Lu; Qing Fei Hua Tan Wan; Qing Fei Yi Huo Pian; Qing Hou Li Yan Ke Li; Qing Kai Ling Ke Li (Jiao Nang, Pian, Zhu She Ye); Qing Xuan Zhi Ke Ke Li; Ren Can Zai Zao Wan; Run Fei Gao; Run Zao Zhi Yang Jiao Nang; San Jin Pian; San Jiu Wei Tai Ke Li (Jiao Nang); Se Chang Zhi Xie San; She Dan Chen Pi Kou Fu Ye; She Dan Chuan Bei Ye; She Xiang Bao Xin Wan; Shen Qi Jiang Tang Pian / Jiao Nang / Ke Li; Sheng Mo Yin (Ke Li, Jiao Nang, Zhu She Ye); Sheng Xue Bao Ke Li; Shi Di Shui; Shu Feng Jie Du Jiao Nang; Shu Gan Ke Li; Shu Mi Tong Jiao Nang; Shu Xin Tong Mo Jiao Nang; Shuang Huang Lian He Ji (Kou Fu Ye, Ke Li, Jiao Nang, Pian); Shuang Huang Lian Zhu She Ye; Si Mo Tang Kou Fu Ye; Si Shen Wan (Pian); Song Ling Xue Mai Kang Jiao Nang; Su Xiao Jiu Xin Wan; Suo Quan Jiao Nang; Tan Ke Jing San (Pian); Tian Ma Xing Nao Jiao Nang; Tian Qi Hua Ye Ke Li; Tian Wang Bu Xin Wan (Pian); Tong Bian Ling Jiao Nang; Tong Xin Luo Jiao Nang; Tong Xuan Li Fei Wan (Ke Li, Jiao Nang, Pian); Wang Bi Ke Li (Jiao Nang, Pian); Wei Su Ke Li; Wen Xin Ke Li; Wu Ji Bai Feng Wan (Jiao Nang, Pian); Xi Gua Shuang Pian; Xi Xin Nao Zhu She Ye; Xiang Guo Jian Xiao Pian; Xiang Sha Ping Wei Wan (Ke Li); Xiao Chai Hu Ke Li; Xiao Er Chang Wei Kang Ke Li; Xiao Er Fei Re Ke Chuan Kou Fu Ye; Xiao Er Hua Du San (Jiao Nang); Xiao Er Hua Tan Zhi Ke Ke Li; Xiao Er Ke Chuan Ling Ke Li; Xiao Er Qing Fei Hua Tan Ke Li; Xiao Er Re Su Qing Kou Fu Ye (Ke Li); Xiao Er Xiao Ji Zhi Ke Kou Fu Ye; Xiao Er Xie Su Ting Ke Li; Xiao Er Yan Bian Ke Li; Xiao Ke Wan; Xiao Yan Pian; Xiao Yao Wan (Ke Li); Xin Fu Fang Da Qing Ye Pian; Xin Ke Shu Jiao Nang (Pian); Xing Pi Yang Er Ke Li; Xiong Dan Shu Gan Li Dan Jiao Nang; Xue Sai Tong Jiao Nang (Pian); Xue Sai Tong Zhu She Ye (Dong Gan); Xue Zhi Kang Pian (Jiao Nang); Xue Fu Zhu Yu Jiao Nang; Yang Xue Qing Nao Wan (Ke Li); Yang Yin Qing Fei Ke Li; Ye Xia Zhu Jiao Nang; Yi Mo Kang Pian; Yi Qing Ke Li; Yi Xin Shu Ke Li (Jiao Nang, Pian); Yin Huang Kou Fu Ye (Ke Li, Jiao Nang, Pian); Yin Qiao Jie Du Wan (Ke Li, Jiao Nang, Ruan Jiao Nang, Pian); Yin Xing Ye Jiao Nang (Pian, Di Wan); Yin Zhi Huang Kou Fu Ye (Ke Li); Yu Ping Feng Ke Li; Yuan Hu Zhi Tong Pian (Ke Li, Jiao Nang, Di Wan); Yun Tong Ding Jiao Nang; Zao Ren An Shen Ke Li (Jiao Nang); Zhen Xiong Dan Wan; Zheng Tian Wan (Jiao Nang); Zheng Wei Jiao Nang / Pian; Zhi Bai Di Huang Wan; Zhi Ke Wan; Zhui Feng Tou Gu Wan; Zu Shi Ma Guan Jie Zhi Tong; Zu Shi Ma Zhu She Ye; Zu Ka Mu Ke Li<br>(Chinese name) 安宫牛黄丸; 安神补脑液; 安胃疡胶囊; 白芨颗粒（粉、糖浆）; 板蓝根颗粒; 板蓝清热颗粒; 保济丸（口服液）; 保和冲剂; 保和丸; 补中益气丸（颗粒）; 参附注射液; 参苓白术散（丸、颗粒）; 参苓健脾胃颗粒; 参麦注射液; 参松养心胶囊; 柴胡注射液; 肠炎宁胶囊（片）; 陈香露白露片; 川贝枇杷膏; 川贝清肺糖浆; 川芎茶调颗粒; 川芎茶调片; 刺五加脑灵液; 大活络丸（胶囊）; 丹参注射液（冻干/粉针）; 丹灯通脑胶囊（软胶囊）; 丹栀逍遥丸; 灯盏花素片; 灯盏花素注射液; 灯盏细辛颗粒; 杜仲降压片; 防风通圣颗粒; 风寒感冒颗粒; 枫蓼肠胃康颗粒; 风热感冒颗粒; 风湿骨痛胶囊; 风湿液; 复方阿胶浆; 复方丹参片（颗粒、胶囊、滴丸）; 复方风湿宁胶囊（片）; 复方龙血竭胶囊; 复方南板蓝根片; 附子理中片/丸; 感冒灵（颗粒、冲剂、胶囊、片）; 感冒清热颗粒（胶囊）; 感冒疏风片（颗粒、丸）; 感冒消炎片; 蛤蚧定喘胶囊/丸; 冠心宁注射液; 归脾丸（合剂）; 寒喘祖帕颗粒; 红花黄色素; 护肝片（颗粒、胶囊）; 虎力散胶囊; 化痰利咽黄氏响声丸; 华佗再造丸; 黄连上清丸（颗粒、胶囊、片）; 黄芪注射液; 黄藤素片（分散片、胶囊、软胶囊）; 藿香正气水（口服液、软胶囊，颗粒）; 急支糖浆（颗粒）; 健儿清解液; 健儿消食口服液; 健脾生血颗粒; 健胃消食片; 金匮肾气丸（片）; 九味羌活颗粒; 橘红丸（颗粒、胶囊、片）; 抗病毒口服液（颗粒）; 抗感灵片; 昆明山海棠片; 利胆止痛胶囊/片; 莲花清瘟胶囊（颗粒）; 六味地黄丸（颗粒、胶囊）; 癃闭舒胶囊; 癃清胶囊; 罗汉果止咳糖浆; 麻仁润肠丸（软胶囊）; 脉络宁注射液; 脉血康胶囊; 脑安滴丸; 脑心舒口服液; 脑心通丸（胶囊、片）; 牛黄解毒丸（胶囊、软胶囊、片）; 牛黄上清丸（胶囊、片）; 普乐安胶囊（片）; 杞菊地黄丸（胶囊、片）; 七叶神安片; 气滞胃痛颗粒（片）; 强力枇杷露; 清肺化痰丸; 清肺抑火片; 清喉利咽颗粒; 清开灵颗粒（胶囊、片、注射液）; 清宣止咳颗粒; 人参再造丸; 润肺膏; 润燥止痒胶囊; 三金片; 三九胃泰颗粒（胶囊）; 涩肠止泻散; 蛇胆陈皮口服液; 蛇胆川贝液; 麝香保心丸; 参芪降糖片/胶囊/颗粒; 生脉饮（颗粒、胶囊、注射液）; 生血宝颗粒; 十滴水; 疏风解毒胶囊; 舒肝颗粒; 舒泌通胶囊; 舒心通脉胶囊; 双黄连合剂（口服液、颗粒、胶囊、片）; 双黄莲注射液; 四磨汤口服

	液; 四神丸（片）; 松龄血脉康胶囊; 速效救心丸; 缩泉胶囊; 痰咳净散（片）; 天麻醒脑胶囊; 田七花叶颗粒; 天王补心丸（片）; 通便灵胶囊; 通心络胶囊; 通宣理肺丸（颗粒、胶囊、片）; 尪痹颗粒（胶囊、片）; 胃苏颗粒; 稳心颗粒; 乌鸡白凤丸（胶囊、片）; 西瓜霜片; 细辛脑注射液; 香果健消片; 香砂平胃丸（颗粒）; 小柴胡颗粒; 小儿肠胃康颗粒; 小儿肺热咳喘口服液; 小儿化毒散（胶囊）; 小儿化痰止咳颗粒; 小儿咳喘灵颗粒; 小儿清肺化痰颗粒; 小儿热速清口服液（颗粒）; 小儿消积止咳口服液; 小儿泻速停颗粒; 小儿咽扁颗粒; 消渴丸; 消炎片; 逍遥丸（颗粒）; 新复方大青叶片; 心可舒胶囊（片）; 醒脾养儿颗粒; 熊胆舒肝利胆胶囊; 血塞通胶囊（片）; 血塞通注射液（冻干）; 血脂康片（胶囊）; 血府逐瘀胶囊; 养血清脑丸（颗粒）; 养阴清肺颗粒; 叶下珠胶囊; 益脉康片; 一清颗粒; 益心舒颗粒（胶囊、片）; 银黄口服液（颗粒、胶囊、片）; 银翘解毒丸（颗粒、胶囊、软胶囊、片）; 银杏叶胶囊（片、滴丸）; 茵栀黄口服液（颗粒）; 玉屏风颗粒; 元胡止痛片（颗粒、胶囊、滴丸）; 晕痛定胶囊; 枣仁安神颗粒（胶囊）; 珍熊胆丸; 正天丸（胶囊）; 正胃胶囊/片; 知柏地黄丸; 止咳丸; 追风透骨丸; 祖师麻关节止痛; 祖师麻注射液; 祖卡木颗粒
Surgical medicine	(Chinese Pinyin) Ji De Sheng She Yao Pian; Jin Qian Cao Ke Li; Kang Fu Xin Ye; Lian Qiao Bai Du Pian; Ma Ying Long Zhi Chuang Gao; Mei Bao Shi Run Shao Shang Gao; Pai Shi Ke Li; Xiao Yan Li Dan Pian (Ke Li, Jiao Nang); Xiao Zhong Zhi Tong Ding<br>(Chinese name) 季德胜蛇药片; 金钱草颗粒; 康复新液; 连翘败毒片; 马应龙痔疮膏; 美宝湿润烧伤膏; 排石颗粒; 消炎利胆片（颗粒、胶囊）; 消肿止痛酊
Gynecological medicine	(Chinese Pinyin) Ba Zhen Yi Mu Wan (Jiao Nang); Bao Fu Kang Shuan; Fu Fang Sha Ji Zi You Shuan ; Fu Ke Qian Jin Pian (Jiao Nang); Fu Ke Shi Wei Pian; Fu Yan Kang Pian ; Fu Yan Xiao Jiao Nang; Geng Nian An Pian (Jiao Nang); Gong Xue Ning Jiao Nang ; Gong Yan Ping Pian; Gui Zhi Fu Ling Wan (Jiao Nang); Hua Hong Pian (Ke Li, Jiao Nang); Jie Er Yin Xi Ye ; Jin Gang Teng Jiao Nang ; Kang Gong Yan Pian /Jiao Nang ; Pen Yan Jing Ke Li (Jiao Nang); Ru Kuai Xiao Ke Li; Ru Pi Xiao Ke Li (Jiao Nang, Pian); Shao Fu Zhu Yu Jiao Nang; Tian Zi Hong Nü Jin Jiao Nang; Yi Mu Cao Gao (Ke Li, Jiao Nang, Pian)<br>(Chinese name) 八珍益母丸（胶囊）; 保妇康栓 ; 复方沙棘籽油栓; 妇科千金片（胶囊）; 妇科十味片; 妇炎康片; 妇炎消胶囊; 更年安片（胶囊）; 宫血宁胶囊; 宫炎平片; 桂枝茯苓丸（胶囊）; 花红片（颗粒、胶囊）; 洁尔阴洗液; 金刚藤胶囊; 抗宫炎片/胶囊; 盆炎净颗粒（胶囊）; 乳块消颗粒; 乳癖消颗粒（胶囊、片）; 少腹逐瘀胶囊; 天紫红女金胶囊; 益母草膏（颗粒、胶囊、片）
Orthopedic medicine	(Chinese Pinyin) Fu Fang Nan Xing Zhi Tong Gao ; Gou Pi Gao ; Gu Tong Ling Ding; Heng Gu Gu Shang Yu He Ji ; Huo Xue Zhi Tong San (Jiao Nang); Huo Xue Zhi Tong San; Jing Fu Kang Ke Li ; Jing Shu Ke Li ; Long Xue Jie Jiao Nang ; San Qi Jiao Nang; Shang Ke Jie Gu Pian ; Shu Jin Huo Xue Wan (Pian， Jiao Nang); Tong Luo Qu Tong Gao ; Tong Shu Jiao Nang ; Wu Di Dan Jiao Nang ; Xian Ling Gu Bao Jiao Nang (Pian); Yao Bi Tong Jiao Nang ; Yun Nan Bai Yao (Jiao Nang, Ke Li, Gao, Ding, Qi Wu Ji); Yun Nan Hong Yao Jiao Nang<br>(Chinese name) 复方南星止痛膏; 狗皮膏; 骨痛灵酊; 恒古骨伤愈合剂; 活血止痛散（胶囊）; 活血止痛散; 颈复康颗粒; 颈舒颗粒; 龙血竭胶囊; 三七胶囊; 伤科接骨片; 舒筋活血丸（片，胶囊）; 通络祛痛膏; 痛舒胶囊; 无敌丹胶囊; 仙灵骨葆胶囊（片）; 腰痹通胶囊; 云南白药(胶囊、颗粒、膏、酊、气雾剂); 云南红药胶囊
Otolaryngology medicine	(Chinese Pinyin) Bi Yan Kang Pian ; Bing Peng San ; Er Long Zuo Ci Wan ; Huo Dan Wan; Kou Yan Qing Ke Li; Tong Qiao Bi Yan Pian ; Xiang Ju Jiao Nang (Pian); Xin Qin Ke Li<br>(Chinese name) 鼻炎康片; 冰硼散; 耳聋左慈丸; 藿胆丸; 口炎清颗粒; 通窍鼻炎片; 香菊胶囊（片）; 辛芩颗粒
Ophthalmic medicine	(Chinese Pinyin) Fu Fang Xue Shuan Tong Jiao Nang (Pian); Ming Mu Di Huang Wan ; Xiong Dan Yan Yao Shui<br>(Chinese name) 复方血栓通胶囊（片）; 明目地黄丸; 熊胆眼药水

Notes: ATC, Anatomical Therapeutic Chemical Classification System; TCM, Traditional Chinese Medicine.
