# Supplementary material for: Longitudinal Trends in Medicine Supply, Price and Utilisation in Primary Care Facilities in Rural Southwestern China Under National Essential Medicines Policy (2012-2017): Disparities Across Facilities and Medicines
Source: Int J Health Policy Manag. 2025 Nov 18;14:8991. doi: 10.34172/ijhpm.8991 (PMC12958208; doi:10.34172/ijhpm.8991)
Supplement: Supplementary file 2 — Description and Group Differences on Medicine Outcomes. [file ijhpm-14-8991-s002.pdf]

Article title: Longitudinal Trends in Medicine Supply, Price and Utilisation in Primary Care Facilities in Rural Southwestern China Under National Essential Medicines Policy (2012-2017): Disparities Across Facilities and Medicines

Journal name: International Journal of Health Policy and Management (IJHPM)

Authors' information: Zhaohua Huo[1¶], Xuechen Xiong[2,3¶], Ge Bai[4], Jianchao Quan[2], Allen TC Lee[1], Linda CW Lam[1], Li Luo[*4]

[1]Department of Psychiatry, Faculty of Medicine, The Chinese University of Hong Kong, Hong Kong SAR, China.

[2]School of Public Health, The University of Hong Kong, Hong Kong SAR, China.

[3]Department of Applied Social Sciences, The Hong Kong Polytechnic University, Hong Kong SAR, China.

[4]School of Public Health, Fudan University, Shanghai, China.

***Correspondence to:** Li Luo; Email: liluo@fudan.edu.cn

¶ Both authors contributed equally to this paper.

Citation: Huo Z, Xiong X, Bai G, et al. Longitudinal trends in medicine supply, price and utilisation in primary care facilities in rural southwestern China under National Essential Medicines Policy (2012-2017): disparities across facilities and medicines. Int J Health Policy Manag. 2025;14:8991. doi:10.34172/ijhpm.8991

Supplementary file 2. Description and Group Differences on Medicine Outcomes

Table S4: Descriptive analysis on medicine numbers, sales and prices

	First-stage NEMP														Second-stage NEMP					
	2012		2013				2014				2015				2016				2017	
	Jul-Sep	Oct-Dec	Jan-Mar	Apr-Jun	Jul-Sep	Oct-Dec	Jan-Mar	Apr-Jun	Jul-Sep	Oct-Dec	Jan-Mar	Apr-Jun	Jul-Sep	Oct-Dec	Jan-Mar	Apr-Jun	Jul-Sep	Oct-Dec	Jan-Mar	Apr-Jun
**Primary care facilities**																				
Number of medicines	**434**	**421**	**417**	**411**	**432**	**408**	**459**	**450**	**440**	**466**	**474**	**477**	**471**	**470**	**462**	**455**	**461**	**457**	**450**	**447**
- NEML	434	421	417	411	432	408	432	423	413	439	440	443	437	436	431	424	430	426	419	416
- Non-NEML	0	0	0	0	0	0	27	27	27	27	34	34	34	34	31	31	31	31	31	31
- Western medicine	235	223	226	219	227	216	250	245	236	245	243	235	234	235	225	219	218	214	212	213
- TCM	199	198	191	192	205	192	209	205	204	221	231	242	237	235	237	236	243	243	238	234
Sales (US$1,000)	**318**	**346**	**355**	**329**	**366**	**308**	**324**	**333**	**261**	**334**	**369**	**375**	**302**	**350**	**418**	**325**	**348**	**427**	**348**	**338**
- NEML	318	346	355	329	366	308	324	333	261	334	364	371	298	345	407	317	337	416	338	327
- Non-NEML	0	0	0	0	0	0	1	1	0	1	5	4	4	4	12	9	10	11	11	11
- Western medicine	180	186	187	166	196	166	173	170	138	177	192	200	158	185	230	163	194	230	189	189
- TCM	138	160	168	163	170	143	152	163	124	157	177	175	145	164	188	162	154	197	159	149
Retail price (DPI-L)[a]	**100.0**	**99.3**	**100.7**	**103.0**	**101.3**	**98.9**	**100.5**	**99.8**	**101.4**	**101.5**	**102.5**	**103.3**	**103.4**	**104.0**	**105.0**	**106.2**	**105.5**	**106.6**	**105.6**	**108.0**
- Western medicine	100.0	100.7	102.7	106.7	103.7	99.3	101.2	100.2	103.4	102.1	103.6	106.8	106.2	107.3	108.8	111.3	110.1	110.9	109.1	114.6
- TCM	100.0	98.0	98.8	99.5	99.1	98.5	100.0	99.3	99.5	101.0	101.4	100.1	100.8	100.8	101.4	101.4	101.3	102.5	102.3	101.9
Retail price (DPI-F)[a]	**100.0**	**99.4**	**100.7**	**102.5**	**101.0**	**98.5**	**100.6**	**99.2**	**101.5**	**101.1**	**102.2**	**102.5**	**102.6**	**103.5**	**104.7**	**105.2**	**104.1**	**105.6**	**104.6**	**106.4**
- Western medicine	100.0	101.0	103.1	106.4	104.3	98.8	101.1	99.4	103.7	101.4	103.5	105.2	105.2	107.3	108.8	111.1	107.4	109.8	108.3	111.4
- TCM	100.0	98.0	98.8	99.4	98.3	98.2	100.1	99.0	99.6	100.8	101.2	100.2	100.5	100.3	101.3	100.9	101.1	102.1	101.5	101.8
Compared with: CPI	100.0	100.0	102.8	102.8	102.8	102.8	104.7	104.7	104.7	104.7	106.0	106.0	106.0	106.0	108.0	108.0	108.0	108.0	109.4	109.4
Compared with: PPI	100.0	100.0	100.6	100.6	100.6	100.6	101.3	101.3	101.3	101.3	101.9	101.9	101.9	101.9	102.2	102.2	102.2	102.2	102.7	102.7
**Township healthcare center**																				
Number of medicines	**383**	**380**	**356**	**342**	**360**	**342**	**384**	**373**	**382**	**393**	**397**	**403**	**398**	**396**	**373**	**383**	**382**	**378**	**392**	**388**
- NEML	383	380	356	342	360	342	357	346	355	366	363	369	364	362	342	352	351	347	361	357
- Non-NEML	0	0	0	0	0	0	27	27	27	27	34	34	34	34	31	31	31	31	31	31
- Western medicine	213	206	198	188	198	185	209	205	210	208	202	201	204	205	188	193	186	186	197	195
- TCM	170	174	158	154	162	157	175	168	172	185	195	202	194	191	185	190	196	192	195	193
Sales (US$1,000)	**147**	**177**	**174**	**150**	**167**	**130**	**149**	**140**	**128**	**147**	**185**	**174**	**150**	**160**	**225**	**166**	**200**	**210**	**177**	**180**
- NEML	147	177	174	150	167	130	149	140	127	146	180	169	146	156	214	157	189	199	167	169
- Non-NEML	0	0	0	0	0	0	1	1	0	1	5	4	4	4	12	9	10	11	11	11
- Western medicine	83	98	98	74	87	80	84	69	68	83	101	99	86	95	136	94	127	128	114	114
- TCM	64	80	76	75	81	50	65	72	60	63	84	74	64	66	90	72	73	82	63	66
Retail price (DPI-L)[a]	**100.0**	**98.6**	**101.6**	**105.3**	**103.1**	**99.3**	**101.1**	**98.9**	**102.3**	**102.0**	**102.1**	**102.4**	**101.3**	**103.1**	**103.9**	**106.9**	**105.8**	**108.0**	**106.5**	**106.4**
- Western medicine	100.0	99.7	104.3	109.9	107.5	97.9	103.0	98.2	103.7	99.8	101.4	104.6	102.4	106.0	105.0	113.7	110.5	114.4	112.1	111.8
- TCM	100.0	97.6	99.2	101.1	99.0	100.5	99.4	99.6	101.1	103.9	102.7	100.4	100.3	100.5	102.9	100.8	101.5	102.3	101.5	101.6
Retail price (DPI-F)[a]	**100.0**	**98.6**	**101.5**	**103.8**	**101.4**	**98.8**	**100.5**	**98.0**	**101.6**	**100.7**	**102.1**	**101.5**	**100.4**	**103.0**	**103.6**	**104.9**	**103.4**	**107.2**	**104.9**	**104.3**
- Western medicine	100.0	100.2	104.5	108.2	107.0	97.5	101.6	96.9	102.2	98.0	101.4	102.1	100.0	105.8	104.9	111.7	106.1	112.8	109.0	107.9
- TCM	100.0	97.1	98.8	100.4	97.0	100.0	99.5	98.9	101.0	103.5	102.6	101.0	100.7	100.4	102.3	99.6	100.8	101.9	101.0	100.8

Compared with: CPI	100.0	100.0	102.8	102.8	102.8	102.8	104.7	104.7	104.7	104.7	106.0	106.0	106.0	106.0	108.0	108.0	108.0	108.0	109.4	109.4
Compared with: PPI	100.0	100.0	100.6	100.6	100.6	100.6	101.3	101.3	101.3	101.3	101.9	101.9	101.9	101.9	102.2	102.2	102.2	102.2	102.7	102.7
**Village clinics**																				
Number of medicines	**409**	**392**	**392**	**385**	**399**	**381**	**397**	**391**	**371**	**399**	**394**	**402**	**392**	**392**	**393**	**378**	**387**	**383**	**380**	**379**
- Western medicine	217	208	211	202	209	201	210	205	190	203	197	193	187	186	185	176	180	175	176	179
- TCM	192	184	181	183	190	180	187	186	181	196	197	209	205	206	208	202	207	208	204	200
Sales (US$1,000)	**170**	**168**	**181**	**179**	**198**	**179**	**175**	**193**	**134**	**187**	**184**	**202**	**152**	**189**	**193**	**160**	**148**	**217**	**171**	**158**
- Western medicine	97	89	89	92	109	86	88	101	70	93	90	101	71	91	95	69	67	101	75	75
- TCM	74	80	92	88	89	93	87	92	64	94	93	101	81	99	99	91	81	116	96	83
Retail price (DPI-L)[c]	**100.0**	**101.0**	**100.8**	**103.2**	**101.2**	**101.2**	**101.8**	**101.5**	**102.3**	**102.6**	**103.3**	**105.9**	**105.3**	**105.0**	**106.5**	**106.3**	**106.7**	**107.6**	**107.7**	**115.0**
- Western medicine	100.0	102.2	102.6	106.9	103.5	102.5	103.7	103.6	105.1	104.4	105.3	110.3	109.0	108.4	110.9	110.6	110.3	110.9	110.7	126.7
- TCM	100.0	99.8	99.0	99.6	99.0	100.0	99.9	99.4	99.5	100.8	101.3	101.6	101.8	101.7	102.1	102.1	103.2	104.4	104.7	103.6
Retail price (DPI-F)[c]	**100.0**	**101.2**	**100.7**	**102.9**	**101.6**	**101.2**	**101.8**	**102.2**	**102.9**	**103.3**	**103.5**	**105.7**	**105.5**	**105.4**	**107.2**	**106.8**	**107.5**	**107.5**	**107.1**	**111.6**
- Western medicine	100.0	103.2	102.3	107.0	104.1	102.8	103.7	104.8	106.7	106.6	106.9	110.7	110.9	110.7	113.9	112.2	112.2	110.9	111.4	120.3
- TCM	100.0	99.7	99.3	99.5	99.3	99.9	100.2	99.9	99.8	100.7	101.1	101.8	101.6	101.6	102.2	103.0	104.1	104.7	104.1	104.1
Compared with: CPI	100.0	100.0	102.8	102.8	102.8	102.8	104.7	104.7	104.7	104.7	106.0	106.0	106.0	106.0	108.0	108.0	108.0	108.0	109.4	109.4
Compared with: PPI	100.0	100.0	100.6	100.6	100.6	100.6	101.3	101.3	101.3	101.3	101.9	101.9	101.9	101.9	102.2	102.2	102.2	102.2	102.7	102.7

a. Drug price index was calculated based on 344 unique medicines (essential medicines only) with records in each period from July 2012 – June 2017 (baseline: 100).

b. Drug price index was calculated based on 258 unique medicines (essential medicines only) with records in each period from July 2012 – June 2017 (baseline: 100).

c. Drug price index was calculated based on 314 unique medicines (essential medicines only) with records in each period from July 2012 – June 2017 (baseline: 100).

Note: CPI, Customer Price Index in local region; DPI-F, Fisher Price Index; DPI-L, Laspeyres Price Index; NEMP, National Essential Medicines Policy; NEML, National Essential Medicines List; PPI, Producer Price Index in pharmaceutical industry.

Table S5: Group differences in Interrupted Time Series Analysis

ITSA estimators by quarter	1st-stage NEMP (July 2012 – December 2015)		2nd-stage NEMP (January 2016 – June 2017)	
	**Difference in Initial Level (Δβ1, P-value)**	**Difference in Initial Trend (Δβ2, P-value)**	**Difference in Level Change (Δβ3, P-value)**	**Difference in Trend Change (Δβ4, P-value)**
**Availability of medicines**	(number)	(number)	(number)	(number)
All primary care facilities				
- Non-essential vs Essential	-421 (P<0.001)	2 (P=0.008)	-6 (P=0.576)	1 (P=0.776)
- TCMs vs Western	-45 (P<0.001)	3 (P=0.008)	21 (P=0.164)	-1 (P=0.789)
- Clinics vs THCs	39 (P<0.001)	-4 (P<0.001)	26 (P=0.096)	-1 (P=0.698)
-- Western: Clinics vs THCs	16 (P=0.006)	-2 (P<0.001)	12 (P=0.200)	0 (P=0.912)
-- TCMs: Clinics vs THCs	23 (P<0.001)	-1 (P=0.012)	14 (P=0.102)	-1 (P=0.635)
THCs				
- Non-essential vs Essential	-362 (P<0.001)	4 (P<0.001)	6 (P=0.611)	-6 (P=0.021)
- TCMs vs Western	-44 (P<0.001)	3 (P=0.001)	11 (P=0.344)	-2 (P=0.340)
-- Essential: TCMs vs Western	-43 (P<0.001)	3 (P<0.001)	4 (P=0.678)	-3 (P=0.215)
-- Non-essential: TCMs vs Western	-1 (P=0.661)	0 (P=0.568)	7 (P=0.037)	0 (P=0.781)
Village clinics				
- TCMs vs Western	-39 (P<0.001)	4 (P<0.001)	13 (P=0.178)	-4 (P=0.102)
**Sales of medicines**	(%)	(%)	(%)	(%)
All primary care facilities				
- Non-essential vs Essential	-100.0 (P<0.001)	55.5 (P<0.001)	15.2 (P=0.825)	-32.0 (P=0.014)
- TCMs vs Western	-13.2 (P=0.057)	0.4 (P=0.603)	0.0 (P=0.997)	-2.6 (P=0.324)
- Clinics vs THCs	10.9 (P=0.325)	-0.1 (P=0.926)	-22.0 (P=0.161)	0.5 (P=0.906)
-- Western: Clinics vs THCs	7.5 (P=0.555)	-1.3 (P=0.292)	-26.6 (P=0.141)	-0.4 (P=0.932)
-- TCMs: Clinics vs THCs	15.4 (P=0.208)	1.3 (P=0.274)	-17.8 (P=0.304)	2.2 (P=0.619)
THCs				
- Non-essential vs Essential	-100.0 (P<0.001)	55.7 (P<0.001)	2.7 (P=0.965)	-31.9 (P=0.010)
- TCMs vs Western	-16.7 (P=0.056)	-1.0 (P=0.284)	-2.8 (P=0.849)	-4.2 (P=0.227)
-- Essential: TCMs vs Western	-17.3 (P=0.047)	-0.7 (P=0.433)	0.6 (P=0.970)	-4.4 (P=0.201)
-- Non-essential: TCMs vs Western	44.2 (P=0.136)	-14.8 (P<0.001)	-23.2 (P=0.487)	18.1 (P=0.068)
Village clinics				
- TCMs vs Western	-10.2 (P=0.237)	1.6 (P=0.082)	9.1 (P=0.555)	-1.4 (P=0.683)
**Retail price** (Essential medicines)	(DPI-F)	(DPI-F)	(DPI-F)	(DPI-F)
All primary care facilities				
- Western vs TCMs	-1.7 (P=0.294)	-0.1 (P=0.416)	-3.9 (P=0.147)	0.2 (P=0.756)
- Clinics vs THCs	-0.7 (P=0.557)	0.3 (P=0.019)	-1.7 (P=0.376)	0.0 (P=0.923)
-- Western: Clinics vs THCs	-1.7 (P=0.480)	0.8 (P=0.003)	-5.6 (P=0.172)	-0.5 (P=0.581)
-- TCMs: Clinics vs THCs	0.1 (P=0.955)	-0.1 (P=0.557)	1.5 (P=0.434)	0.5 (P=0.267)
THCs				
- Western vs TCMs	-3.2 (P=0.244)	0.3 (P=0.257)	-6.7 (P=0.155)	-0.7 (P=0.516)
Village clinics				
- Western vs TCMs	-1.5 (P=0.308)	-0.5 (P=0.002)	0.4 (P=0.879)	0.2 (P=0.686)

Note: DPI-F, Fisher Price Index; ITSA, interrupted time series analysis; NEMP, National Essential Medicines Policy; TCM, traditional Chinese Medicine; THC, township healthcare centres.

Figure S1. Non-essential medicines in township healthcare centres

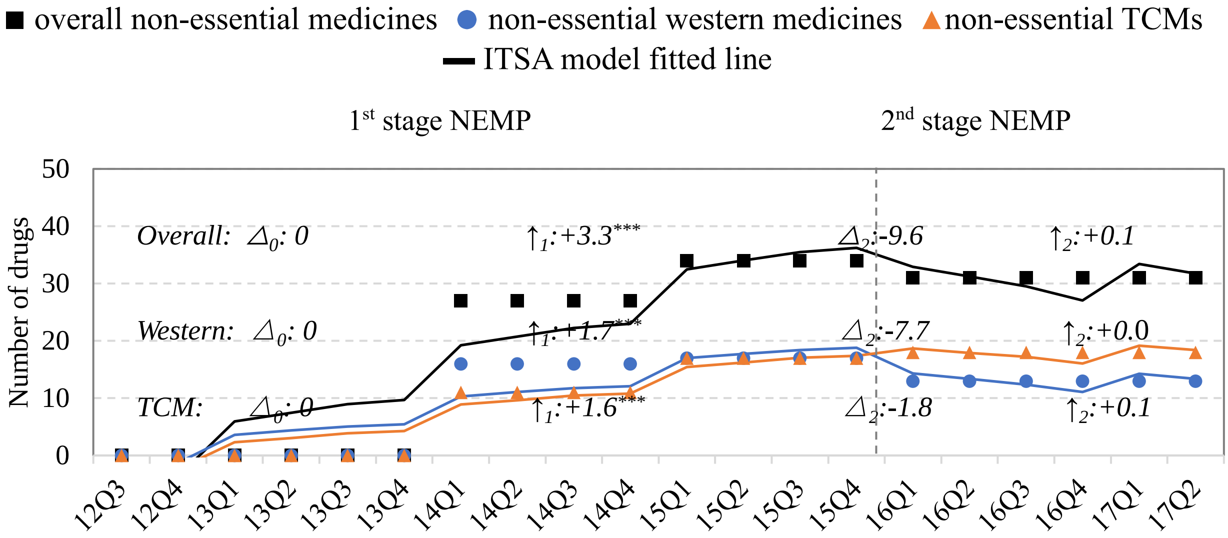

(a) number of non-essential medicines

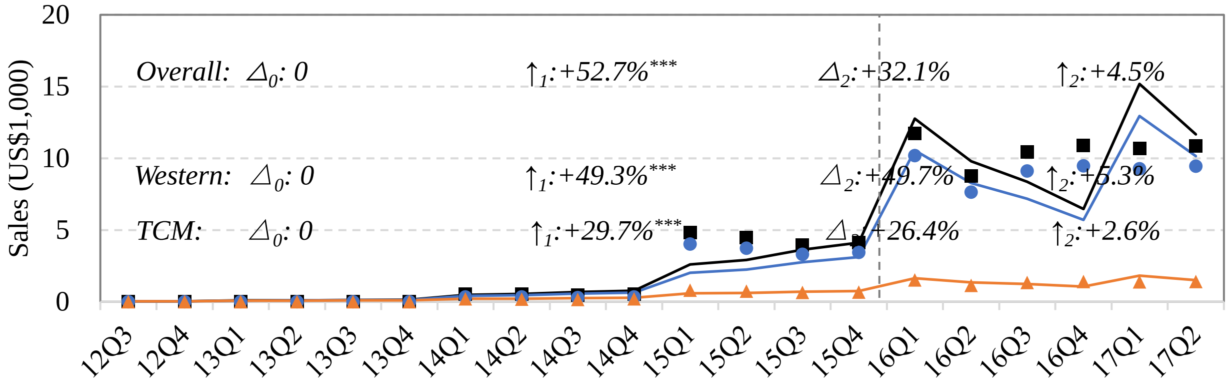

(b) sales of non-essential medicines

$\triangle_0$: initial level (β_0); $\uparrow_1$: sustained change per observation period during first-phase NEMP (β_1); $\triangle_2$: immediate change after second-phase NEMP (β_2); $\uparrow_2$: sustained change per observation period after second-phase NEMP (β_1+β_3); ITSA, interrupted time-series analysis; NEMP, National Essential Medicines Policy; TCM, Traditional Chinese Medicine; *, P<0.05; **, P<0.01; ***, P<0.001
