# Supplementary material for: Longitudinal Trends in Medicine Supply, Price and Utilisation in Primary Care Facilities in Rural Southwestern China Under National Essential Medicines Policy (2012-2017): Disparities Across Facilities and Medicines
Source: Int J Health Policy Manag. 2025 Nov 18;14:8991. doi: 10.34172/ijhpm.8991 (PMC12958208; doi:10.34172/ijhpm.8991)
Supplement: Supplementary file 3 — Medicine Number and Sales by ATC System and TCM Classification. [file ijhpm-14-8991-s003.pdf]

Article title: Longitudinal Trends in Medicine Supply, Price and Utilisation in Primary Care Facilities in Rural Southwestern China Under National Essential Medicines Policy (2012-2017): Disparities Across Facilities and Medicines

Journal name: International Journal of Health Policy and Management (IJHPM)

Authors' information: Zhaohua Huo[1¶], Xuechen Xiong[2,3¶], Ge Bai[4], Jianchao Quan[2], Allen TC Lee[1], Linda CW Lam[1], Li Luo[*4]

[1]Department of Psychiatry, Faculty of Medicine, The Chinese University of Hong Kong, Hong Kong SAR, China.

[2]School of Public Health, The University of Hong Kong, Hong Kong SAR, China.

[3]Department of Applied Social Sciences, The Hong Kong Polytechnic University, Hong Kong SAR, China.

[4]School of Public Health, Fudan University, Shanghai, China.

***Correspondence to:** Li Luo; Email: liluo@fudan.edu.cn

¶ Both authors contributed equally to this paper.

Citation: Huo Z, Xiong X, Bai G, et al. Longitudinal trends in medicine supply, price and utilisation in primary care facilities in rural southwestern China under National Essential Medicines Policy (2012-2017): disparities across facilities and medicines. Int J Health Policy Manag. 2025;14:8991. doi:10.34172/ijhpm.8991

Supplementary file 3. Medicine Number and Sales by ATC System and TCM Classification

Figure S2. Number of medicines by ATC system and TCM classification

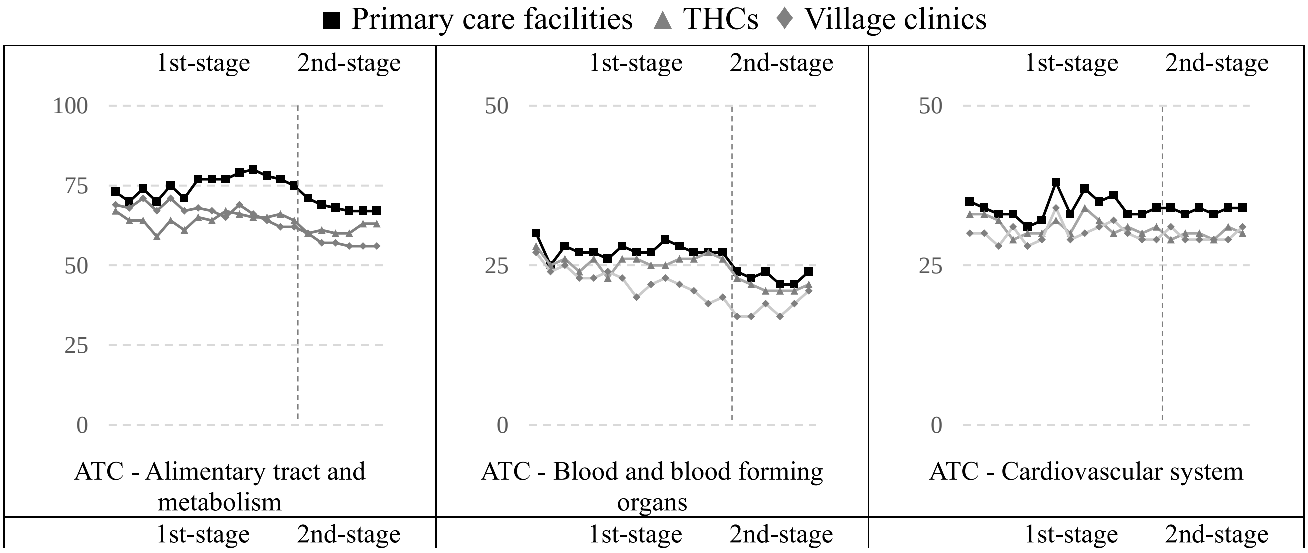

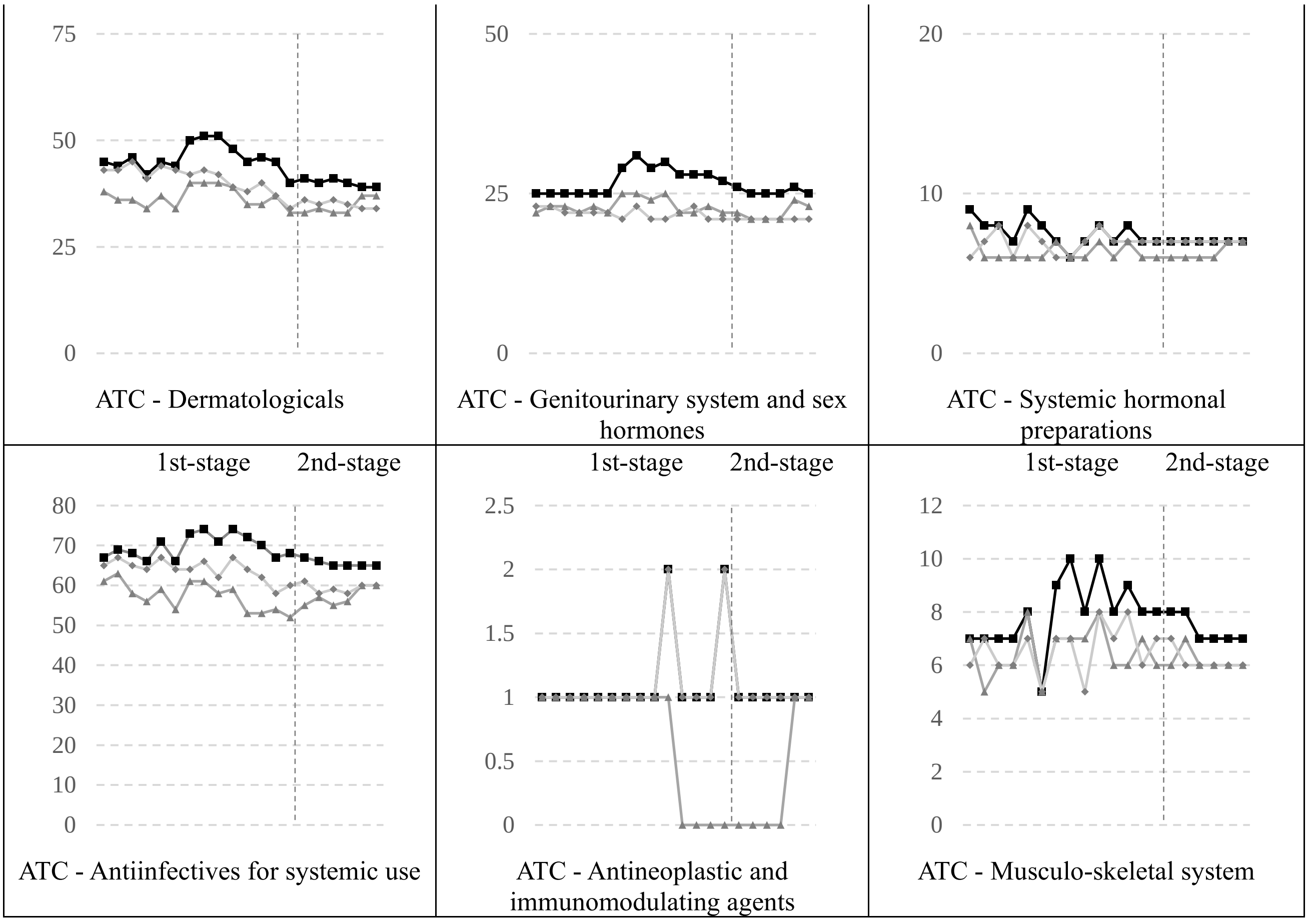
75
50
25
0
ATC - Dermatologicals
50
25
0
ATC - Genitourinary system and sex hormones
20
10
0
ATC - Systemic hormonal preparations
1st-stage
2nd-stage
80
70
60
50
40
30
20
10
0
ATC - Antiinfectives for systemic use
1st-stage
2nd-stage
2.5
2
1.5
1
0.5
0
ATC - Antineoplastic and immunomodulating agents
1st-stage
2nd-stage
12
10
8
6
4
2
0
ATC - Musculo-skeletal system

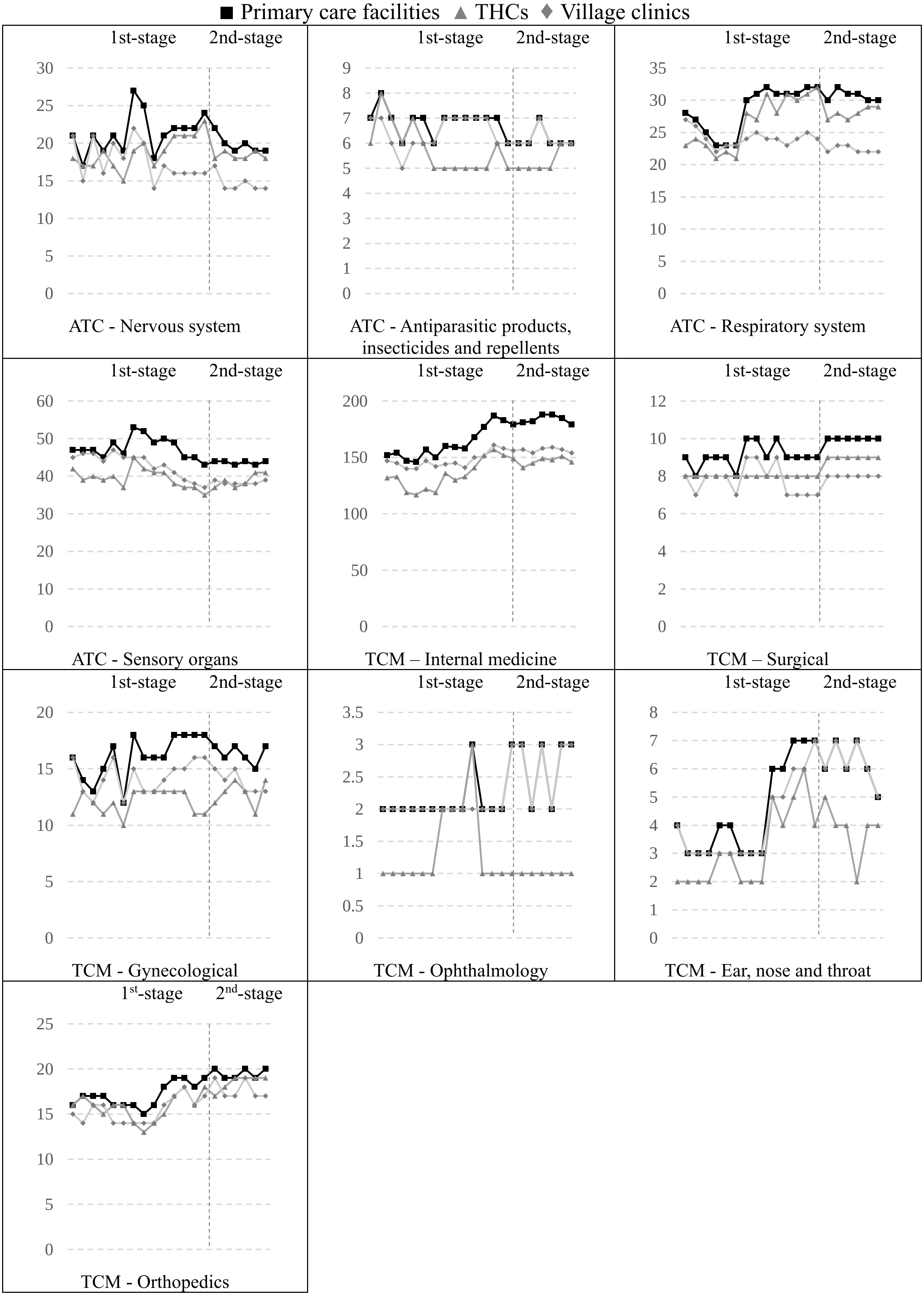
■ Primary care facilities ▲ THCs ♦ Village clinics
1st-stage
2nd-stage
ATC - Nervous system
ATC - Antiparasitic products, insecticides and repellents
ATC - Respiratory system
ATC - Sensory organs
TCM – Internal medicine
TCM – Surgical
TCM - Gynecological
TCM - Ophthalmology
TCM - Ear, nose and throat
TCM - Orthopedics

Table S6. Interrupted time-series analysis on medicine number

Facility	Drug category	Baseline β_0	Time after 1st-stage NEMP β_1 (*p-value*)	2nd-stage NEMP β_2 (*p-value*)	Time after 2nd-stage NEMP β_3 (*p-value*)
All primary care facilities	Overall	417	5.1 (<.001)	-15.8 (0.278)	-7.2 (0.044)
	- Essential drug	418	1.6 (0.015)	-5.2 (0.602)	-3.9 (0.100)
	- Non-essential drug	-1	3.3 (<.001)	-9.6 (0.181)	-3.2 (0.105)
	Western medicine	231	1.1 (0.112)	-17.8 (0.093)	-3.1 (0.207)
	- Essential drug	231	-0.6 (0.094)	-9.3 (0.099)	-1.6 (0.197)
	- Non-essential drug	0	1.7 (<.001)	-7.7 (0.096)	-1.7 (0.182)
	TCM	188	3.7 (<.001)	2.9 (0.782)	-3.9 (0.147)
	- Essential drug	189	2.0 (0.009)	4.6 (0.616)	-2.4 (0.323)
	- Non-essential drug	-1	1.6 (<.001)	-1.8 (0.523)	-1.5 (0.057)
	By ATC-classification:				
	- Alimentary tract and metabolism	73	0.6 (0.002)	-8.6 (0.002)	-1.2 (0.036)
	- Blood and blood forming organs	28	0.0 (0.754)	-3.8 (0.002)	-0.2 (0.454)
	- Cardiovascular system	35	0.1 (0.555)	-1.7 (0.408)	0.1 (0.797)
	- Dermatological	46	-0.1 (0.734)	-0.8 (0.810)	-0.6 (0.574)
	- Genito urinary system and sex hormones	26	0.2 (0.172)	-2.1 (0.268)	-0.5 (0.327)
	- Systemic hormonal preparations	8	-0.1 (0.041)	0.1 (0.839)	0.1 (0.513)
	- Anti-infective for systemic use	70	0.2 (0.416)	-4.0 (0.211)	-0.6 (0.456)
	- Antineoplastic and immunomodulating agents	1	0.0 (0.021)	-0.2 (0.331)	-0.1 (0.284)
	- Musculo-skeletal system	7	0.2 (0.023)	-1.0 (0.348)	-0.4 (0.132)
	- Nervous system	21	0.3 (0.069)	-2.1 (0.364)	-0.7 (0.199)
	- Antiparasitic, insecticides and repellents	7	0.0 (0.328)	-0.3 (0.558)	0.0 (0.919)
	- Respiratory system	25	0.5 (0.054)	-2.2 (0.428)	-0.5 (0.507)
	- Sensory organs	49	-0.2 (0.560)	-2.1 (0.516)	0.0 (0.967)
	By TCM-classification:				
	- Internal medicine	144	2.8 (<.001)	2.8 (0.722)	-3.0 (0.161)
	- Surgical	9	0.0 (0.196)	0.5 (0.358)	0.0 (0.792)
	- Gynecological	14	0.3 (<.001)	-1.5 (0.144)	-0.5 (0.059)
	- Orthopedics	16	0.2 (0.125)	1.1 (0.319)	-0.1 (0.778)
	- Ear, nose and throat	2	0.3 (0.008)	0.3 (0.794)	-0.5 (0.132)
	- Ophthalmology	2	0.0 (0.060)	0.1 (0.747)	0.0 (0.934)
THCs	Overall	362	2.9 (0.046)	-28.9 (0.131)	0.8 (0.867)
	- Essential drug	363	-0.4 (0.683)	-19.0 (0.199)	4.0 (0.278)
	- Non-essential drug	-1	3.3 (<.001)	-9.6 (0.181)	-3.2 (0.105)
	Western medicine	203	0.2 (0.708)	-20.3 (0.048)	1.7 (0.477)
	- Essential drug	202	-1.5 (0.004)	-11.1 (0.117)	3.2 (0.062)
	- Non-essential drug	0	1.7 (<.001)	-7.7 (0.096)	-1.7 (0.182)
	TCM	160	2.6 (0.013)	-8.3 (0.466)	-0.9 (0.779)
	- Essential drug	161	1.0 (0.217)	-6.4 (0.505)	0.6 (0.807)
	- Non-essential drug	-1	1.6 (<.001)	-1.8 (0.523)	-1.5 (0.057)
	By ATC-classification:				
	- Alimentary tract and metabolism	64	0.1 (0.412)	-6.5 (0.011)	0.6 (0.266)
	- Blood and blood forming organs	26	0.0 (0.627)	-3.7 (0.009)	-0.2 (0.486)
	- Cardiovascular system	32	-0.1 (0.428)	-1.7 (0.296)	0.4 (0.349)
	- Dermatological	38	-0.1 (0.652)	-3.9 (0.173)	0.9 (0.244)
	- Genito urinary system and sex hormones	24	0.0 (0.866)	-2.6 (0.101)	0.4 (0.291)
	- Systemic hormonal preparations	7	0.0 (0.663)	-0.8 (0.196)	0.3 (0.074)
	- Anti-infective for systemic use	61	-0.6 (0.008)	0.2 (0.955)	1.6 (0.043)
	- Antineoplastic and immunomodulating agents	1	-0.1 (0.011)	-0.5 (0.262)	0.3 (0.012)
	- Musculo-skeletal system	6	0.0 (0.309)	-0.4 (0.615)	-0.1 (0.527)
	- Nervous system	17	0.4 (0.002)	-3.4 (0.048)	-0.4 (0.301)
	- Antiparasitic products, insecticides and repellents	7	-0.2 (0.012)	-0.2 (0.804)	0.4 (0.061)
	- Respiratory system	22	0.9 (<.001)	-6.4 (0.015)	-0.3 (0.646)

	- Sensory organs	43	-0.3 (0.120)	-1.9 (0.475)	1.1 (0.098)
	By TCM-classification:				
	- Internal medicine	123	2.2 (0.016)	-10.2 (0.292)	-0.8 (0.771)
	- Surgical	n.a.	n.a.	n.a.	n.a.
	- Gynecological	12	0.0 (0.712)	0.3 (0.837)	0.0 (0.926)
	- Orthopedics	15	0.1 (0.498)	-0.4 (0.821)	0.4 (0.354)
	- Ear, nose and throat	1	0.3 (0.002)	-0.4 (0.755)	-0.4 (0.111)
	- Ophthalmology	1	0.0 (0.805)	-0.1 (0.857)	-0.1 (0.744)
Village clinics	Overall (Essential drug)	395	-0.1 (0.903)	-3.1 (0.715)	-1.6 (0.431)
	Western medicine (Essential drug)	217	-1.9 (<.001)	-8.5 (0.029)	1.0 (0.235)
	TCM (Essential drug)	180	1.7 (0.011)	6.0 (0.469)	-2.6 (0.201)
	By ATC-classification:				
	- Alimentary tract and metabolism	71	-0.5 (<.001)	-4.6 (0.010)	-0.1 (0.829)
	- Blood and blood forming organs	26	-0.4 (<.001)	-3.9 (0.006)	1.1 (0.001)
	- Cardiovascular system	31	0.1 (0.465)	-1.2 (0.364)	0.0 (0.905)
	- Dermatological	45	-0.6 (<.001)	-0.8 (0.691)	0.2 (0.655)
	- Genito urinary system and sex hormones	22	-0.1 (0.024)	-0.2 (0.759)	0.1 (0.665)
	- Systemic hormonal preparations	7	0.0 (0.575)	-0.1 (0.913)	0.0 (0.961)
	- Anti-infective for systemic use	67	-0.4 (0.003)	-2.1 (0.295)	0.5 (0.331)
	- Antineoplastic and immunomodulating agents	1	0.0 (0.021)	-0.2 (0.331)	-0.1 (0.284)
	- Musculo-skeletal system	6	0.1 (0.045)	-0.5 (0.397)	-0.2 (0.128)
	- Nervous system	21	-0.3 (0.036)	-0.5 (0.786)	-0.1 (0.877)
	- Antiparasitic products, insecticides and repellents	6	0.0 (0.970)	0.0 (0.967)	0.0 (0.816)
	- Respiratory system	25	-0.1 (0.360)	-1.2 (0.388)	0.1 (0.850)
	- Sensory organs	48	-0.7 (<.001)	0.2 (0.909)	0.6 (0.203)
	By TCM-classification:				
	- Internal medicine	140	1.1 (0.016)	3.0 (0.588)	-1.4 (0.308)
	- Surgical	8	0.0 (0.436)	0.5 (0.591)	0.0 (0.852)
	- Gynecological	13	0.1 (0.085)	0.1 (0.948)	-0.5 (0.094)
	- Orthopedics	15	0.2 (0.172)	2.1 (0.176)	-0.4 (0.263)
	- Ear, nose and throat	3	0.3 (0.025)	0.2 (0.859)	-0.4 (0.241)
	- Ophthalmology	2	0.0 (0.183)	0.2 (0.511)	0.0 (0.765)

Notes: Segmented linear regression model was built with two interruption points: $Y_t = \beta_0 + \beta_1 T + \beta_2 X_{1\_t} + \beta_3 T X_{1\_t} + \varepsilon_t$, Coefficient β0 estimates the baseline level of outcome; β1 estimates the time trend of outcome during the first-stage; β2 estimates the immediate changes in level after the second-stage policy; and β3 estimates the sustained change in trend after the second-stage policy; ATC, Anatomical Therapeutic Chemical; TCM, Traditional Chinese Medicine; NEMP, National Essential Medicines Policy.

Figure S3. Sales of medicines by ATC system and TCM classification (US$1,000)

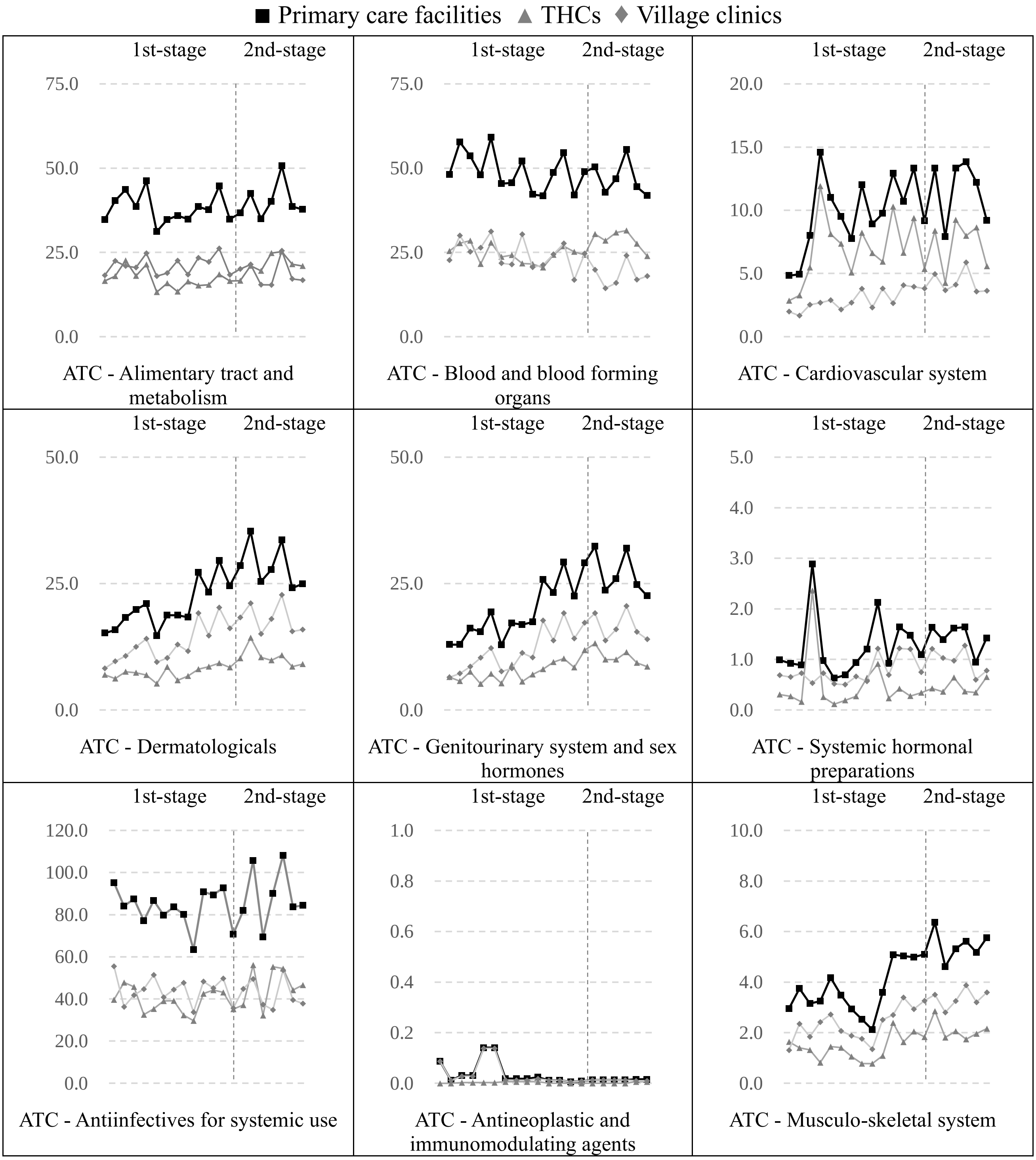

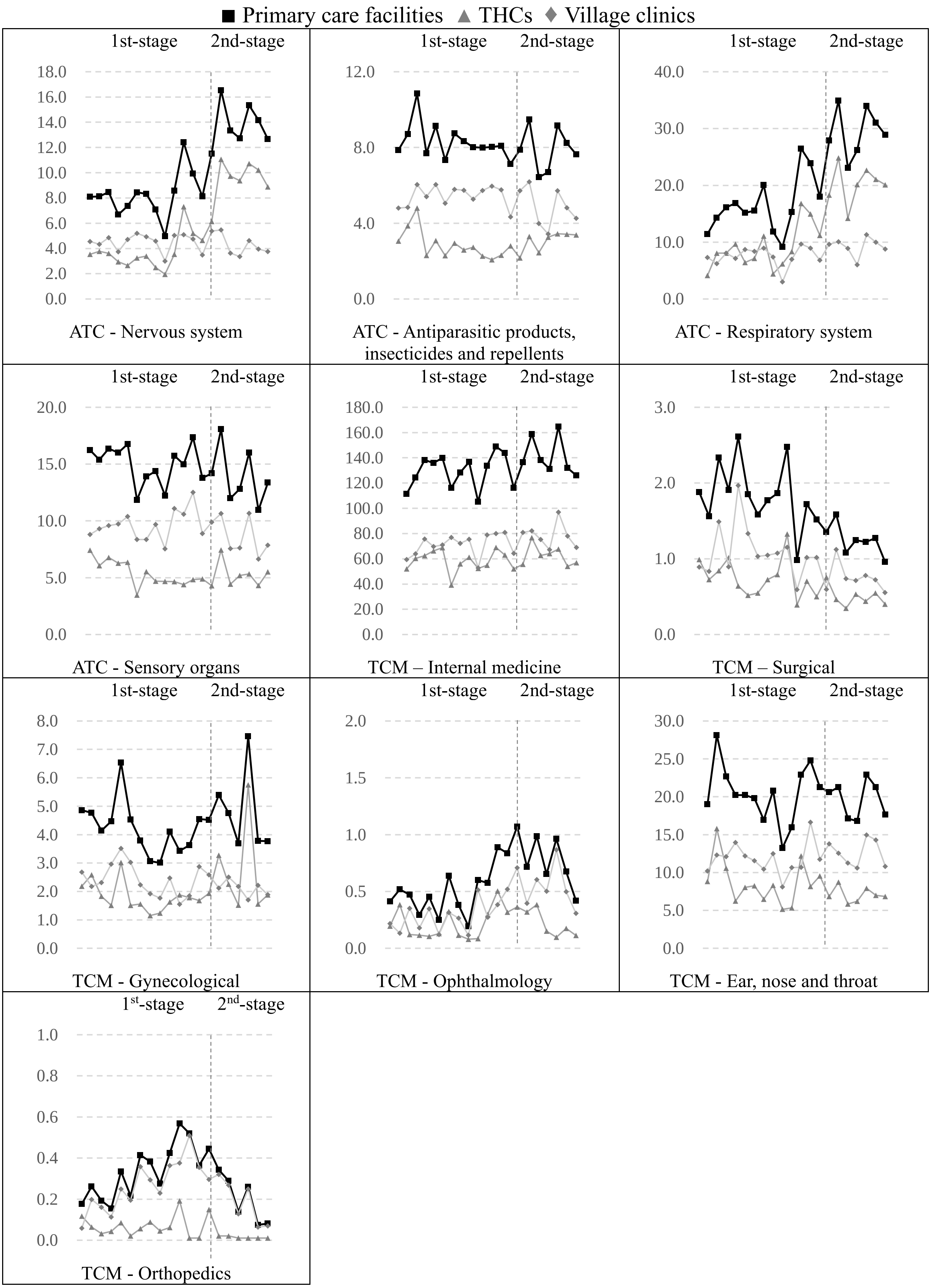
■ Primary care facilities ▲ THCs ♦ Village clinics
1st-stage 2nd-stage
ATC - Nervous system
ATC - Antiparasitic products, insecticides and repellents
ATC - Respiratory system
ATC - Sensory organs
TCM – Internal medicine
TCM – Surgical
TCM - Gynecological
TCM - Ophthalmology
TCM - Ear, nose and throat
TCM - Orthopedics

Table S7. Interrupted time-series analysis on medicine sales after log transformation

Facility	Drug category	Baseline $exp(\beta_0)$ (US$1,000)	Time after 1st-stage NEMP: $exp(\beta_1)$-1 (*p-value*)	2nd-stage NEMP $exp(\beta_2)$-1 (*p-value*)	Time after 2nd-stage NEMP $exp(\beta_3)$-1 (*p-value*)
All primary care facilities	Overall	348.2	0.0% (1.000)	15.9% (0.175)	-1.7% (0.477)
	- Essential drug	349.0	-0.1% (0.852)	14.1% (0.211)	-1.7% (0.478)
	- Non-essential drug	0.0	52.7% (<.001)	32.1% (0.702)	-31.6% (0.058)
	Western medicine	186.4	-0.2% (0.803)	16.0% (0.195)	-0.5% (0.841)
	- Essential drug	187.1	-0.3% (0.590)	12.8% (0.266)	-0.4% (0.871)
	- Non-essential drug	0.0	49.3% (<.001)	49.7% (0.573)	-29.5% (0.078)
	TCM	161.6	0.2% (0.723)	15.2% (0.194)	-3.1% (0.208)
	- Essential drug	161.7	0.2% (0.771)	14.9% (0.201)	-3.1% (0.208)
	- Non-essential drug	0.0	29.6% (<.001)	26.4% (0.607)	-20.8% (0.059)
	By ATC-classification:				
	- Alimentary tract and metabolism	39.1	-0.3% (0.681)	8.8% (0.540)	0.5% (0.873)
	- Blood and blood forming organs	52.8	-1.2% (0.098)	10.1% (0.404)	-0.6% (0.817)
	- Cardiovascular system	7.4	4.7% (0.050)	-5.0% (0.882)	-6.4% (0.417)
	- Dermatological	15.4	4.6% (<.001)	21.5% (0.219)	-8.9% (0.019)
	- Genito urinary system and sex hormones	13.1	6.2% (<.001)	9.7% (0.541)	-9.2% (0.014)
	- Systemic hormonal preparations	0.8	1.8% (0.494)	47.6% (0.348)	-9.3% (0.313)
	- Anti-infective for systemic use	89.6	-0.4% (0.542)	11.5% (0.337)	0.2% (0.952)
	- Antineoplastic and immunomodulating	0.1	-5.3% (0.068)	14.9% (0.730)	6.3% (0.538)
	- Musculo-skeletal system	3.1	3.1% (0.210)	26.7% (0.380)	-2.8% (0.707)
	- Nervous system	8.5	1.8% (0.216)	52.4% (0.049)	-3.2% (0.516)
	- Antiparasitic, insecticides and repellents	9.9	-1.0% (0.052)	-3.5% (0.671)	2.1% (0.296)
	- Respiratory system	15.7	4.4% (0.056)	14.8% (0.600)	-1.7% (0.806)
	- Sensory organs	15.7	-0.6% (0.484)	9.0% (0.522)	-3.7% (0.239)
	By TCM-classification:				
	- Internal medicine	132.3	0.4% (0.461)	16.5% (0.138)	-3.5% (0.134)
	- Surgical	2.1	-2.5% (0.033)	-5.7% (0.750)	-1.4% (0.736)
	- Gynecological	4.3	-1.9% (0.192)	59.8% (0.059)	-3.4% (0.517)
	- Orthopedics	0.2	6.9% (<.001)	-13.5% (0.471)	-25.7% (<.001)
	- Ear, nose and throat	0.4	5.8% (0.042)	33.5% (0.487)	-13.6% (0.142)
	- Ophthalmology	22.4	-0.7% (0.637)	3.0% (0.894)	-0.4% (0.943)
THCs	Overall	165.1	0.0% (0.974)	32.3% (0.064)	-2.0% (0.542)
	- Essential drug	165.8	-0.2% (0.802)	29.0% (0.076)	-2.0% (0.528)
	- Non-essential drug	0.0	52.7% (<.001)	32.1% (0.702)	-31.6% (0.058)
	Western medicine	90.2	0.4% (0.713)	35.6% (0.065)	-0.3% (0.926)
	- Essential drug	90.7	0.1% (0.953)	30.3% (0.087)	-0.2% (0.951)
	- Non-essential drug	0.0	49.3% (<.001)	49.7% (0.573)	-29.5% (0.078)
	TCM	74.9	-0.5% (0.580)	28.4% (0.105)	-4.3% (0.208)
	- Essential drug	75.1	-0.6% (0.502)	27.8% (0.108)	-4.3% (0.202)
	- Non-essential drug	0.0	29.6% (<.001)	26.4% (0.607)	-20.8% (0.059)
	By ATC-classification:				
	- Alimentary tract and metabolism	18.1	-0.9% (0.436)	32.1% (0.123)	2.5% (0.550)
	- Blood and blood forming organs	26.0	-0.6% (0.496)	35.7% (0.025)	-2.5% (0.407)
	- Cardiovascular system	5.5	4.5% (0.121)	-21.1% (0.587)	-3.7% (0.711)
	- Dermatological	6.8	2.8% (0.007)	49.4% (0.017)	-9.9% (0.009)
	- Genito urinary system and sex hormones	6.3	4.6% (<.001)	29.5% (0.040)	-10.2% (0.001)
	- Systemic hormonal preparations	0.3	0.5% (0.889)	16.9% (0.811)	0.2% (0.992)
	- Anti-infective for systemic use	43.4	-0.5% (0.604)	22.2% (0.204)	1.6% (0.660)
	- Antineoplastic and immunomodulating	0.1	-0.1% (0.858)	-6.4% (0.249)	2.3% (0.174)
	- Musculo-skeletal system	1.4	1.8% (0.569)	36.0% (0.392)	-1.0% (0.920)
	- Nervous system	3.9	3.4% (0.278)	65.6% (0.086)	-1.3% (0.883)
	- Antiparasitic, insecticides and repellents	3.8	-3.3% (0.008)	23.4% (0.252)	8.4% (0.067)

	- Respiratory system	7.1	7.5% (0.007)	27.4% (0.491)	-4.6% (0.570)
	- Sensory organs	6.8	-3.2% (0.005)	40.9% (0.056)	-0.6% (0.870)
	By TCM-classification:				
	- Internal medicine	61.2	-0.1% (0.860)	30.6% (0.078)	-5.0% (0.133)
	- Surgical	0.8	-2.2% (0.178)	-25.9% (0.274)	3.6% (0.568)
	- Gynecological	1.9	-1.9% (0.273)	103.1% (0.027)	-4.9% (0.454)
	- Orthopedics	0.1	-0.1% (0.961)	-27.0% (0.403)	-5.2% (0.536)
	- Ear, nose and throat	0.2	4.1% (0.347)	10.7% (0.860)	-17.4% (0.199)
	- Ophthalmology	10.7	-2.1% (0.297)	-0.4% (0.991)	1.6% (0.828)
Village clinics	Overall (Essential drug)	182.6	0.0% (0.977)	0.5% (0.964)	-1.1% (0.650)
	Western medicine (Essential drug)	96.2	-0.7% (0.321)	-5.3% (0.653)	-0.3% (0.926)
	TCM (Essential drug)	86.4	0.8% (0.234)	5.7% (0.617)	-2.2% (0.384)
	By ATC-classification:				
	- Alimentary tract and metabolism	20.8	0.3% (0.673)	-15.0% (0.261)	-0.6% (0.851)
	- Blood and blood forming organs	26.9	-1.7% (0.078)	-21.6% (0.130)	2.8% (0.439)
	- Cardiovascular system	2.0	5.4% (<.001)	15.2% (0.380)	-7.6% (0.045)
	- Dermatological	8.7	5.6% (<.001)	8.0% (0.680)	-8.3% (0.059)
	- Genito urinary system and sex hormones	6.8	7.3% (<.001)	3.2% (0.885)	-9.5% (0.064)
	- Systemic hormonal preparations	0.5	3.8% (0.024)	44.1% (0.166)	-12.7% (0.033)
	- Anti-infective for systemic use	45.8	-0.2% (0.806)	0.8% (0.955)	-1.2% (0.695)
	- Antineoplastic and immunomodulating	0.1	-5.3% (0.070)	23.8% (0.602)	4.1% (0.689)
	- Musculo-skeletal system	1.7	4.1% (0.063)	15.1% (0.610)	-3.3% (0.627)
	- Nervous system	5.0	0.1% (0.847)	0.9% (0.936)	-3.7% (0.161)
	- Antiparasitic, insecticides and repellents	6.1	0.3% (0.670)	-15.0% (0.194)	-1.6% (0.574)
	- Respiratory system	8.6	0.5% (0.807)	8.7% (0.781)	0.4% (0.960)
	- Sensory organs	9.0	0.9% (0.283)	-4.7% (0.734)	-5.3% (0.111)
	By TCM-classification:				
	- Internal medicine	70.8	1.0% (0.124)	6.0% (0.557)	-2.2% (0.329)
	- Surgical	1.3	-2.4% (0.151)	13.8% (0.643)	-4.9% (0.439)
	- Gynecological	2.4	-1.4% (0.447)	8.5% (0.752)	-1.5% (0.808)
	- Orthopedics	0.2	8.4% (<.001)	-10.6% (0.624)	-26.6% (<.001)
	- Ear, nose and throat	0.2	6.7% (0.003)	35.0% (0.341)	-9.6% (0.171)
	- Ophthalmology	11.6	0.5% (0.737)	1.7% (0.934)	-1.6% (0.742)

Notes: Segmented linear regression model was built with two interruption points: $Y_t = \beta_0 + \beta_1 T + \beta_2 X_{1\_t} + \beta_3 T X_{1\_t} + \varepsilon_t$, Coefficient β0 estimates the baseline level of outcome; β1 estimates the time trend of outcome during the first-stage; β2 estimates the immediate changes in level after the second-stage policy; and β3 estimates the sustained change in trend after the second-stage policy; ATC, Anatomical Therapeutic Chemical; TCM, Traditional Chinese Medicine; NEMP, National Essential Medicines Policy.
