# Supplementary material for: Longitudinal Trends in Medicine Supply, Price and Utilisation in Primary Care Facilities in Rural Southwestern China Under National Essential Medicines Policy (2012-2017): Disparities Across Facilities and Medicines
Source: Int J Health Policy Manag. 2025 Nov 18;14:8991. doi: 10.34172/ijhpm.8991 (PMC12958208; doi:10.34172/ijhpm.8991)
Supplement: Supplementary file 4 — Medicine Price by ATC System and TCM Classification. [file ijhpm-14-8991-s004.pdf]

Article title: Longitudinal Trends in Medicine Supply, Price and Utilisation in Primary Care Facilities in Rural Southwestern China Under National Essential Medicines Policy (2012-2017): Disparities Across Facilities and Medicines

Journal name: International Journal of Health Policy and Management (IJHPM)

Authors' information: Zhaohua Huo[1¶], Xuechen Xiong[2,3¶], Ge Bai[4], Jianchao Quan[2], Allen TC Lee[1], Linda CW Lam[1], Li Luo[*4]

[1]Department of Psychiatry, Faculty of Medicine, The Chinese University of Hong Kong, Hong Kong SAR, China.

[2]School of Public Health, The University of Hong Kong, Hong Kong SAR, China.

[3]Department of Applied Social Sciences, The Hong Kong Polytechnic University, Hong Kong SAR, China.

[4]School of Public Health, Fudan University, Shanghai, China.

***Correspondence to:** Li Luo; Email: liluo@fudan.edu.cn

¶ Both authors contributed equally to this paper.

Citation: Huo Z, Xiong X, Bai G, et al. Longitudinal trends in medicine supply, price and utilisation in primary care facilities in rural southwestern China under National Essential Medicines Policy (2012-2017): disparities across facilities and medicines. Int J Health Policy Manag. 2025;14:8991. doi:10.34172/ijhpm.8991

Supplementary file 4. Medicine Price by ATC System and TCM Classification

Figure S4. Prices of medicines by ATC system and TCM classification (Fisher Price Index)

▲ THCs ♦ Village clinics

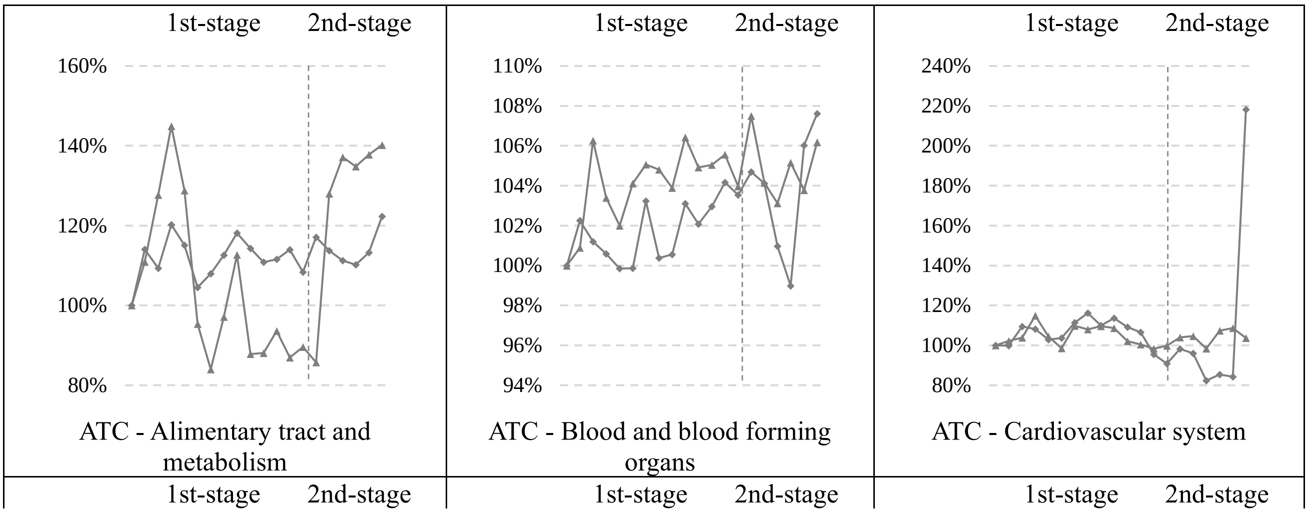

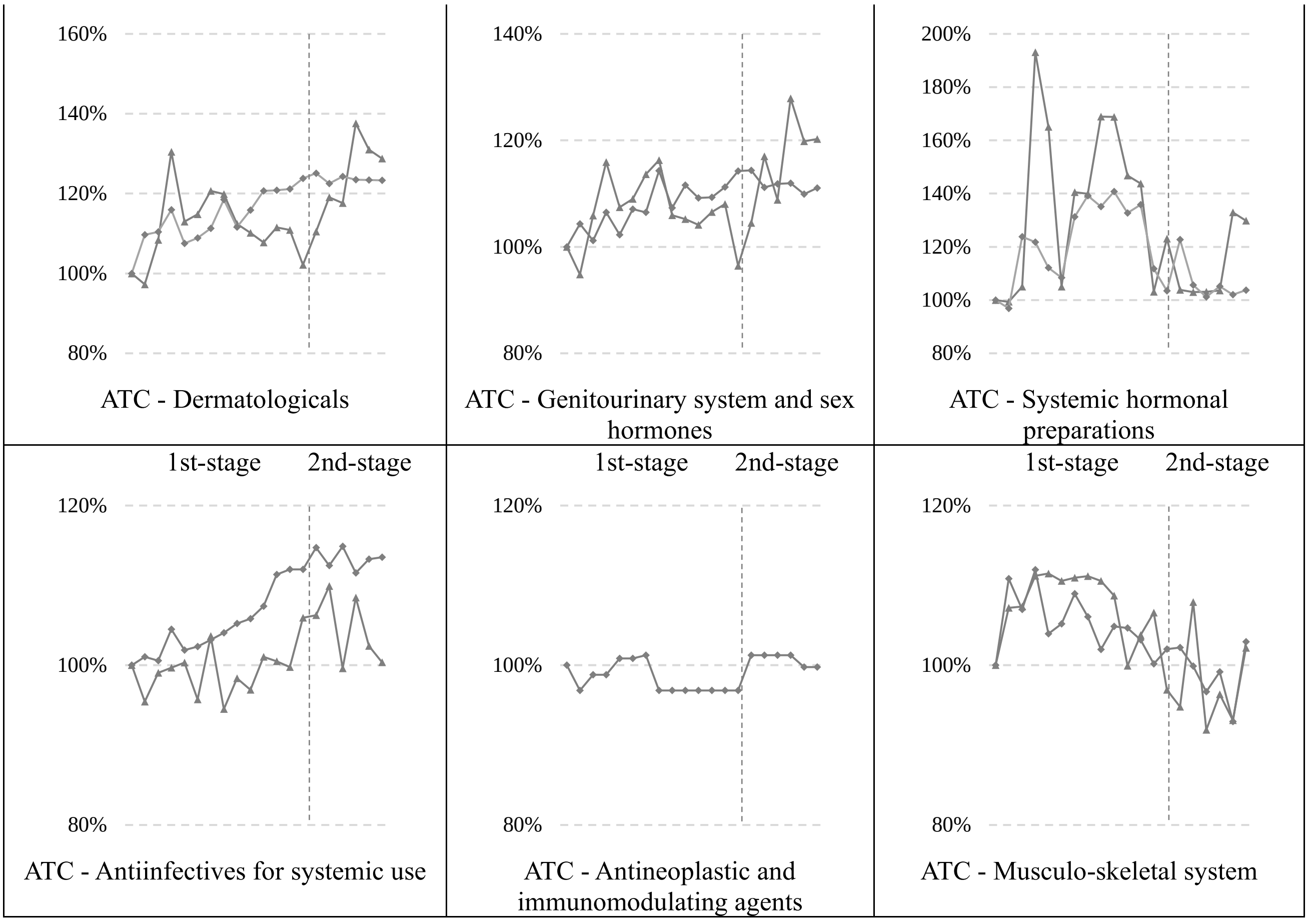
160%
140%
120%
100%
80%
ATC - Dermatologicals
ATC - Genitourinary system and sex hormones
200%
180%
160%
ATC - Systemic hormonal preparations
1st-stage
2nd-stage
ATC - Antiinfectives for systemic use
ATC - Antineoplastic and immunomodulating agents
ATC - Musculo-skeletal system

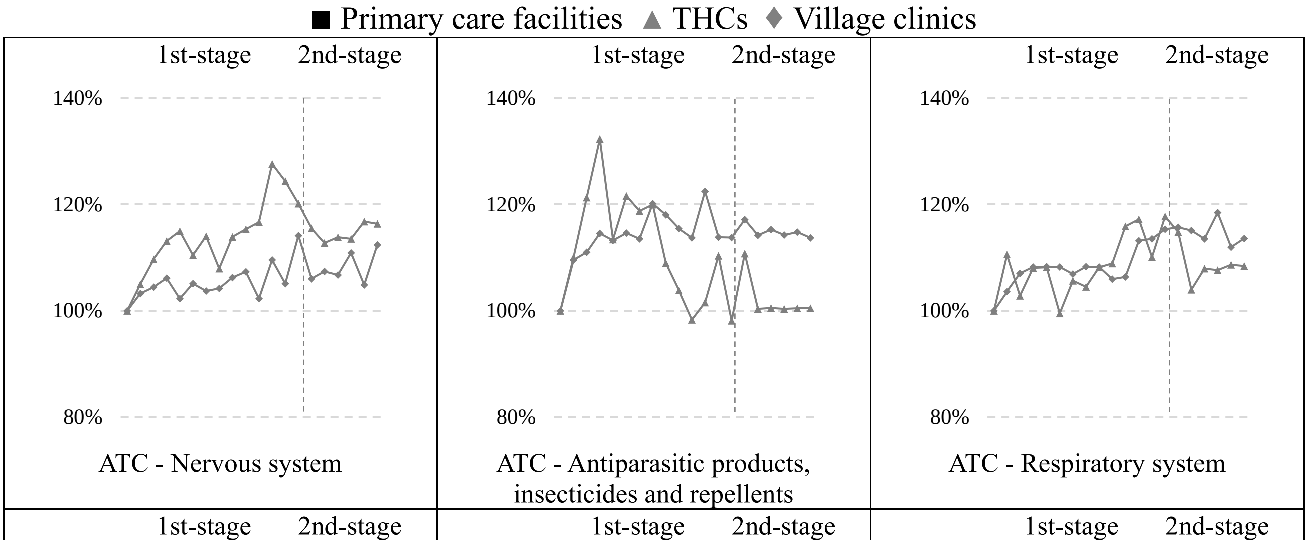
■ Primary care facilities ▲ THCs ♦ Village clinics
1st-stage
2nd-stage
140%
120%
100%
80%
ATC - Nervous system
ATC - Antiparasitic products, insecticides and repellents
ATC - Respiratory system

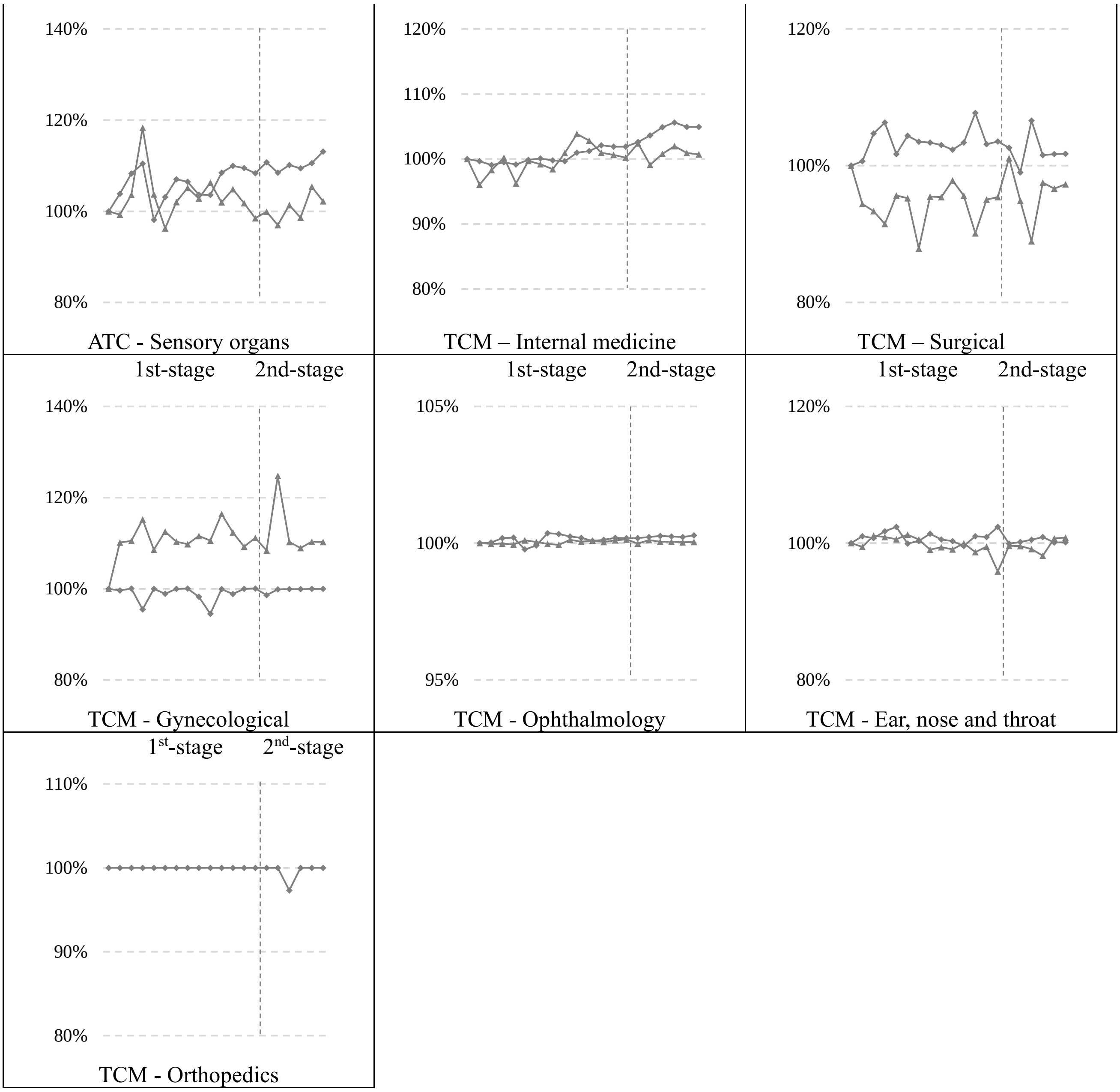
140%
120%
100%
80%
ATC - Sensory organs
120%
110%
100%
90%
80%
TCM – Internal medicine
120%
100%
80%
TCM – Surgical
1st-stage
2nd-stage
140%
120%
100%
80%
TCM - Gynecological
1st-stage
2nd-stage
105%
100%
95%
TCM - Ophthalmology
1st-stage
2nd-stage
120%
100%
80%
TCM - Ear, nose and throat
1st-stage
2nd-stage
110%
100%
90%
80%
TCM - Orthopedics

Table S8. Interrupted time-series analysis on medicine retail prices

Part I. Drug Price Index – Fisher (DPI-F)

Facility	Drug category	Number of drugs included	Time after 1st-stage NEMP: $\exp(\beta_1)-1$ (*p-value*)	2nd-stage NEMP $\exp(\beta_2)-1$ (*p-value*)	Time after 2nd-stage NEMP $\exp(\beta_3)-1$ (*p-value*)
All primary care facilities	Overall (Essential drug)	344	0.2% (0.016)	1.5% (0.260)	0.0% (0.968)
	Western medicine (Essential drug)	169	0.3% (0.096)	3.0% (0.299)	-0.1% (0.942)
	TCM (Essential drug)	175	0.2% (0.002)	0.0% (0.988)	0.0% (0.854)
	By ATC-classification (Essential drug):				
	- Alimentary tract and metabolism	55	-0.7% (0.384)	2.2% (0.833)	4.4% (0.105)
	- Blood and blood forming organs	19	0.3% (<.001)	-1.3% (0.181)	-0.1% (0.661)
	- Cardiovascular system	26	-0.2% (0.779)	-7.5% (0.311)	2.1% (0.273)
	- Dermatological	33	0.4% (0.259)	1.8% (0.708)	0.2% (0.895)
	- Genito urinary system and sex hormones	22	0.1% (0.715)	2.0% (0.530)	0.0% (0.962)
	- Systemic hormonal preparations	6	0.9% (0.576)	-17.7% (0.410)	-3.1% (0.560)
	- Anti-infective for systemic use	56	0.5% (0.009)	5.0% (0.067)	-1.1% (0.088)
	- Antineoplastic and immunomodulating	1	-0.2% (0.090)	5.6% (0.004)	-0.3% (0.446)
	- Musculo-skeletal system	5	-0.5% (0.004)	-2.5% (0.295)	0.0% (0.974)
	- Nervous system	13	1.0% (<.001)	-7.3% (0.025)	-0.1% (0.927)
	- Antiparasitic, insecticides and repellents	6	0.2% (0.467)	0.2% (0.966)	-0.8% (0.413)
	- Respiratory system	21	1.1% (<.001)	-1.1% (0.763)	-2.0% (0.033)
	- Sensory organs	36	0.1% (0.710)	-0.7% (0.809)	0.5% (0.479)
	By TCM-classification (Essential drug):				
	- Internal medicine	138	0.2% (0.004)	0.1% (0.958)	0.0% (0.850)
	- Surgical	8	0.2% (0.009)	-2.0% (0.137)	-0.3% (0.317)
	- Gynecological	11	0.2% (0.055)	-1.0% (0.551)	-0.1% (0.726)
	- Orthopedics	13	0.0% (0.028)	-0.3% (0.020)	0.0% (0.352)
	- Ear, nose and throat	3	0.0% (0.750)	-0.5% (0.422)	-0.1% (0.727)
	- Ophthalmology	2	0.0% (0.934)	-0.6% (0.119)	0.1% (0.528)
THCs	Overall (Essential drug)	258	0.1% (0.373)	2.5% (0.193)	0.1% (0.806)
	Western medicine (Essential drug)	135	-0.1% (0.603)	7.1% (0.083)	0.3% (0.701)
	TCM (Essential drug)	123	0.3% (0.010)	-0.9% (0.561)	-0.3% (0.472)
	By ATC-classification (Essential drug):				
	- Alimentary tract and metabolism	43	-1.6% (0.297)	-0.9% (0.962)	10.5% (0.054)
	- Blood and blood forming organs	17	0.3% (<.001)	-1.1% (0.295)	-0.4% (0.108)
	- Cardiovascular system	24	-0.2% (0.516)	-0.1% (0.991)	0.6% (0.689)
	- Dermatological	21	0.1% (0.924)	0.9% (0.931)	2.9% (0.288)
	- Genito urinary system and sex hormones	14	0.0% (0.967)	0.7% (0.927)	2.4% (0.220)
	- Systemic hormonal preparations	5	1.4% (0.557)	-50.0% (0.158)	3.2% (0.696)
	- Anti-infective for systemic use	40	0.3% (0.048)	9.0% (0.001)	-1.7% (0.004)
	- Antineoplastic and immunomodulating	0	n.a.	n.a.	n.a.
	- Musculo-skeletal system	4	-0.4% (0.247)	-5.3% (0.363)	0.0% (0.972)
	- Nervous system	12	1.5% (<.001)	-10.3% (0.034)	-1.1% (0.312)
	- Antiparasitic, insecticides and repellents	5	-1.1% (0.148)	5.0% (0.634)	-1.0% (0.698)
	- Respiratory system	18	1.0% (0.004)	-4.8% (0.318)	-1.4% (0.201)
	- Sensory organs	27	-0.1% (0.656)	-5.9% (0.232)	1.1% (0.330)
	By TCM-classification (Essential drug):				
	- Internal medicine	93	0.3% (0.009)	-1.2% (0.508)	-0.3% (0.519)
	- Surgical	7	-0.1% (0.818)	2.5% (0.525)	0.0% (0.968)
	- Gynecological	8	0.2% (0.273)	4.7% (0.201)	-1.7% (0.050)
	- Orthopedics	12	0.0% (<.001)	0.0% (0.344)	0.0% (0.141)
	- Ear, nose and throat	2	-0.2% (0.006)	0.0% (0.985)	0.5% (0.046)
	- Ophthalmology	0	n.a.	n.a.	n.a.
Village clinics	Overall (Essential drug)	314	0.4% (<.001)	0.6% (0.582)	0.2% (0.441)
	Western medicine (Essential drug)	152	0.7% (<.001)	0.6% (0.805)	0.0% (0.992)
	TCM (Essential drug)	162	0.2% (0.009)	0.7% (0.264)	0.2% (0.211)
	By ATC-classification (Essential drug):				
	- Alimentary tract and metabolism	53	0.2% (0.531)	-1.2% (0.825)	0.4% (0.766)
	- Blood and blood forming organs	15	0.2% (0.126)	-1.9% (0.410)	0.4% (0.506)
	- Cardiovascular system	22	-0.1% (0.897)	-44.8% (0.017)	11.4% (0.011)

	- Dermatological	32	1.3% (<.001)	1.6% (0.577)	-1.7% (0.025)
	- Genito urinary system and sex hormones	19	0.9% (<.001)	1.0% (0.637)	-1.6% (0.004)
	- Systemic hormonal preparations	6	0.7% (0.600)	6.0% (0.671)	-6.1% (0.174)
	- Anti-infective for systemic use	51	0.9% (<.001)	3.0% (0.133)	-1.2% (0.022)
	- Antineoplastic and immunomodulating	1	-0.2% (0.127)	4.6% (0.019)	0.0% (0.976)
	- Musculo-skeletal system	5	-0.5% (<.001)	-0.8% (0.576)	-0.4% (0.198)
	- Nervous system	12	0.5% (0.001)	-1.5% (0.459)	-0.1% (0.824)
	- Antiparasitic, insecticides and repellents	5	0.7% (0.008)	-2.1% (0.596)	-1.4% (0.143)
	- Respiratory system	20	0.8% (0.003)	3.4% (0.287)	-1.4% (0.100)
	- Sensory organs	34	0.4% (0.026)	-0.6% (0.822)	0.0% (0.971)
	By TCM-classification (Essential drug):				
	- Internal medicine	127	0.2% (0.009)	1.0% (0.207)	0.3% (0.227)
	- Surgical	7	0.2% (0.195)	-2.6% (0.225)	-0.1% (0.759)
	- Gynecological	11	0.0% (0.970)	-0.3% (0.884)	0.3% (0.511)
	- Orthopedics	12	0.0% (0.233)	-0.1% (0.693)	0.0% (0.982)
	- Ear, nose and throat	3	0.0% (0.688)	-0.7% (0.438)	0.0% (0.920)
	- Ophthalmology	2	0.0% (0.934)	-0.9% (0.119)	0.1% (0.528)

Part II. Drug Price Index – Laspeyres (DPI-L)

Facility	Drug category	Number of drugs included	Time after 1st-stage NEMP: $\exp(\beta_1)-1$ (*p-value*)	2nd-stage NEMP $\exp(\beta_2)-1$ (*p-value*)	Time after 2nd-stage NEMP $\exp(\beta_3)-1$ (*p-value*)
All primary care facilities	Overall (Essential drug)	344	0.3% (0.010)	1.1% (0.416)	0.2% (0.610)
	Western medicine (Essential drug)	169	0.4% (0.040)	1.8% (0.489)	0.3% (0.612)
	TCM (Essential drug)	175	0.2% (<.001)	0.1% (0.922)	0.0% (0.761)
	By ATC-classification (Essential drug):				
	- Alimentary tract and metabolism	55	-0.7% (0.421)	2.0% (0.859)	4.6% (0.117)
	- Blood and blood forming organs	19	0.1% (0.020)	-0.6% (0.477)	-0.1% (0.647)
	- Cardiovascular system	26	0.3% (0.635)	-20.2% (0.090)	5.0% (0.073)
	- Dermatological	33	1.0% (<.001)	-2.0% (0.559)	-0.7% (0.367)
	- Genito urinary system and sex hormones	22	0.2% (0.256)	0.0% (0.988)	-0.3% (0.664)
	- Systemic hormonal preparations	6	2.4% (0.353)	-31.4% (0.299)	-6.3% (0.445)
	- Anti-infective for systemic use	56	0.6% (0.016)	2.9% (0.217)	-0.7% (0.256)
	- Antineoplastic and immunomodulating	1	-0.2% (0.090)	5.6% (0.004)	-0.3% (0.446)
	- Musculo-skeletal system	5	0.0% (0.998)	0.4% (0.879)	0.5% (0.432)
	- Nervous system	13	1.5% (<.001)	-8.7% (0.078)	-0.2% (0.888)
	- Antiparasitic, insecticides and repellents	6	0.3% (0.180)	-0.9% (0.813)	-0.8% (0.348)
	- Respiratory system	21	0.9% (<.001)	-2.8% (0.103)	-0.8% (0.050)
	- Sensory organs	36	0.5% (0.023)	-3.1% (0.244)	0.2% (0.735)
	By TCM-classification (Essential drug):				
	- Internal medicine	138	0.2% (0.001)	0.3% (0.780)	0.0% (0.822)
	- Surgical	8	0.1% (0.294)	-0.8% (0.592)	-0.5% (0.180)
	- Gynecological	11	0.1% (0.276)	-1.8% (0.374)	0.4% (0.447)
	- Orthopedics	13	0.0% (0.007)	-0.5% (0.003)	0.1% (0.122)
	- Ear, nose and throat	3	0.0% (0.693)	-0.1% (0.879)	0.0% (0.845)
	- Ophthalmology	2	0.0% (0.934)	-0.2% (0.119)	0.0% (0.528)
THCs	Overall (Essential drug)	258	0.1% (0.338)	2.5% (0.238)	0.2% (0.617)
	Western medicine (Essential drug)	135	0.0% (0.931)	5.6% (0.165)	0.8% (0.389)
	TCM (Essential drug)	123	0.2% (0.099)	0.7% (0.653)	-0.3% (0.435)
	By ATC-classification (Essential drug):				
	- Alimentary tract and metabolism	43	-1.8% (0.263)	1.4% (0.947)	12.0% (0.042)
	- Blood and blood forming organs	17	0.2% (0.010)	-0.8% (0.513)	-0.3% (0.301)
	- Cardiovascular system	24	0.0% (0.982)	2.6% (0.730)	-0.7% (0.710)
	- Dermatological	21	0.4% (0.549)	-3.9% (0.603)	-0.2% (0.938)
	- Genito urinary system and sex hormones	14	0.0% (0.938)	-0.5% (0.931)	-0.2% (0.910)
	- Systemic hormonal preparations	5	3.7% (0.210)	-95.7% (0.036)	5.6% (0.585)
	- Anti-infective for systemic use	40	0.5% (0.005)	5.2% (0.065)	-1.7% (0.015)
	- Antineoplastic and immunomodulating	0	n.a.	n.a.	n.a.
	- Musculo-skeletal system	4	0.4% (0.135)	-0.6% (0.864)	-0.4% (0.656)
	- Nervous system	12	2.2% (<.001)	-11.0% (0.066)	-1.6% (0.221)

	- Antiparasitic, insecticides and repellents	5	-1.1% (0.162)	5.7% (0.595)	-1.0% (0.690)
	- Respiratory system	18	0.7% (<.001)	-4.4% (0.105)	0.2% (0.783)
	- Sensory organs	27	0.1% (0.616)	-8.0% (0.011)	1.4% (0.048)
	By TCM-classification (Essential drug):				
	- Internal medicine	93	0.2% (0.110)	0.8% (0.695)	-0.3% (0.490)
	- Surgical	7	0.0% (0.800)	5.5% (0.090)	-1.1% (0.137)
	- Gynecological	8	0.4% (0.093)	-1.7% (0.650)	-0.5% (0.584)
	- Orthopedics	12	0.0% (<.001)	-0.1% (0.006)	0.0% (0.148)
	- Ear, nose and throat	2	-0.1% (0.212)	-0.5% (0.263)	0.3% (0.038)
	- Ophthalmology	0	n.a.	n.a.	n.a.
Village clinics	Overall (Essential drug)	314	0.4% (0.002)	-1.2% (0.454)	0.9% (0.024)
	Western medicine (Essential drug)	152	0.5% (0.005)	-2.6% (0.355)	1.4% (0.043)
	TCM (Essential drug)	162	0.2% (0.014)	0.4% (0.598)	0.3% (0.235)
	By ATC-classification (Essential drug):				
	- Alimentary tract and metabolism	53	0.3% (0.472)	-2.1% (0.748)	0.2% (0.877)
	- Blood and blood forming organs	15	0.1% (0.154)	0.3% (0.842)	-0.4% (0.230)
	- Cardiovascular system	22	1.1% (0.712)	-142.1% (0.019)	45.2% (0.003)
	- Dermatological	32	1.6% (<.001)	-2.2% (0.587)	-1.7% (0.098)
	- Genito urinary system and sex hormones	19	0.4% (0.004)	-0.7% (0.668)	-0.4% (0.314)
	- Systemic hormonal preparations	6	1.6% (0.597)	3.5% (0.891)	-9.6% (0.270)
	- Anti-infective for systemic use	51	0.6% (0.002)	1.8% (0.372)	-0.2% (0.640)
	- Antineoplastic and immunomodulating	1	-0.2% (0.127)	4.6% (0.019)	0.0% (0.976)
	- Musculo-skeletal system	5	-0.4% (0.015)	-0.3% (0.896)	1.1% (0.051)
	- Nervous system	12	0.7% (0.001)	-2.0% (0.492)	-0.1% (0.905)
	- Antiparasitic, insecticides and repellents	5	1.2% (<.001)	-5.3% (0.198)	-1.1% (0.249)
	- Respiratory system	20	0.8% (<.001)	0.2% (0.925)	-1.2% (0.050)
	- Sensory organs	34	1.0% (<.001)	-3.5% (0.333)	-0.4% (0.597)
	By TCM-classification (Essential drug):				
	- Internal medicine	127	0.2% (0.016)	0.6% (0.540)	0.3% (0.240)
	- Surgical	7	0.0% (0.956)	-2.8% (0.366)	0.0% (0.974)
	- Gynecological	11	0.0% (0.847)	-0.3% (0.154)	0.1% (0.080)
	- Orthopedics	12	0.0% (0.045)	0.0% (0.992)	0.0% (0.775)
	- Ear, nose and throat	3	0.0% (0.583)	-0.8% (0.372)	-0.1% (0.800)
	- Ophthalmology	2	0.0% (0.934)	-0.6% (0.119)	0.1% (0.528)

Notes: Segmented linear regression model was built with two interruption points: $Y_t = \beta_0 + \beta_1 T + \beta_2 X_{1\_t} + \beta_3 T X_{1\_t} + \varepsilon_t$, Coefficient β0 estimates the baseline level of outcome; β1 estimates the time trend of outcome during the first-stage; β2 estimates the immediate changes in level after the second-stage policy; and β3 estimates the sustained change in trend after the second-stage policy; ATC, Anatomical Therapeutic Chemical; TCM, Traditional Chinese Medicine; NEMP, National Essential Medicines Policy.
