# Supplementary material for: Longitudinal Trends in Medicine Supply, Price and Utilisation in Primary Care Facilities in Rural Southwestern China Under National Essential Medicines Policy (2012-2017): Disparities Across Facilities and Medicines
Source: Int J Health Policy Manag. 2025 Nov 18;14:8991. doi: 10.34172/ijhpm.8991 (PMC12958208; doi:10.34172/ijhpm.8991)
Supplement: Supplementary file 5 — Evidence Integration in Secondary Care Facilities. [file ijhpm-14-8991-s005.pdf]

Article title: Longitudinal Trends in Medicine Supply, Price and Utilisation in Primary Care Facilities in Rural Southwestern China Under National Essential Medicines Policy (2012-2017): Disparities Across Facilities and Medicines

Journal name: International Journal of Health Policy and Management (IJHPM)

Authors' information: Zhaohua Huo[1¶], Xuechen Xiong[2,3¶], Ge Bai[4], Jianchao Quan[2], Allen TC Lee[1], Linda CW Lam[1], Li Luo[*4]

[1]Department of Psychiatry, Faculty of Medicine, The Chinese University of Hong Kong, Hong Kong SAR, China.

[2]School of Public Health, The University of Hong Kong, Hong Kong SAR, China.

[3]Department of Applied Social Sciences, The Hong Kong Polytechnic University, Hong Kong SAR, China.

[4]School of Public Health, Fudan University, Shanghai, China.

***Correspondence to:** Li Luo; Email: liluo@fudan.edu.cn

¶ Both authors contributed equally to this paper.

Citation: Huo Z, Xiong X, Bai G, et al. Longitudinal trends in medicine supply, price and utilisation in primary care facilities in rural southwestern China under National Essential Medicines Policy (2012-2017): disparities across facilities and medicines. Int J Health Policy Manag. 2025;14:8991. doi:10.34172/ijhpm.8991

Supplementary file 5. Evidence Integration in Secondary Care Facilities

Figure S5. Available number of medicines in primary and secondary care facilities

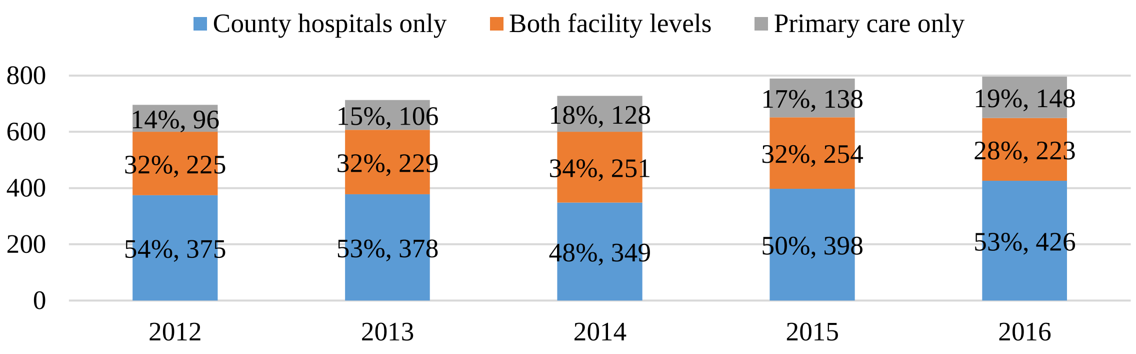

(a) Overall medicines (%, n)

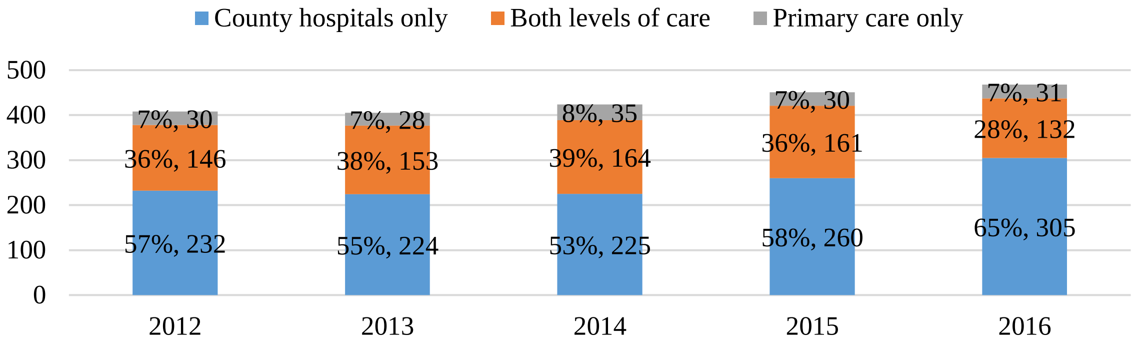

(b) Western medicines (%, n)

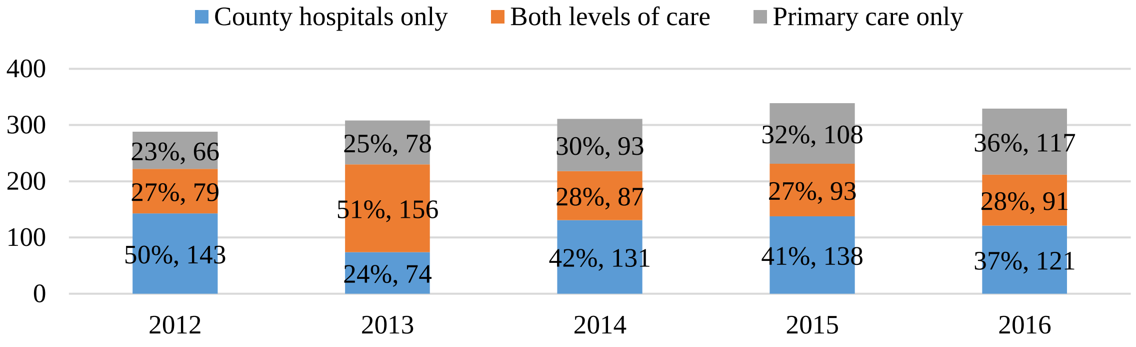

(c) Traditional Chinese medicines (%, n)

Figure S6. Available number of medicines in township health centres and village clinics

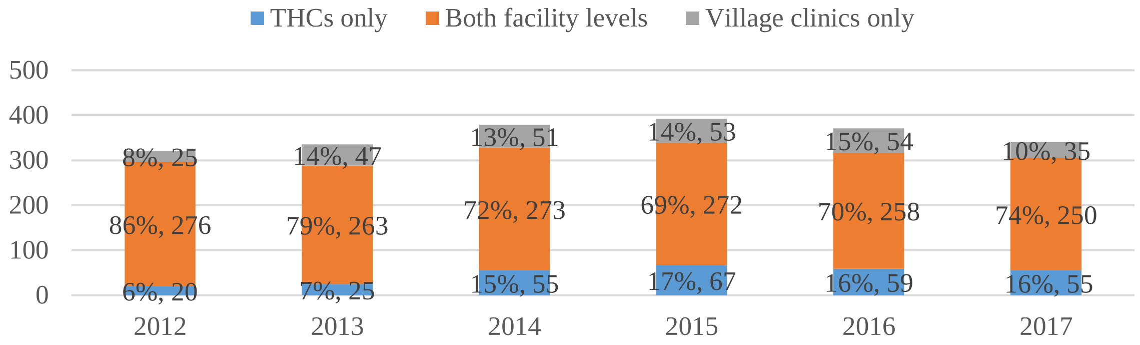

(a) Overall medicines (%, n)

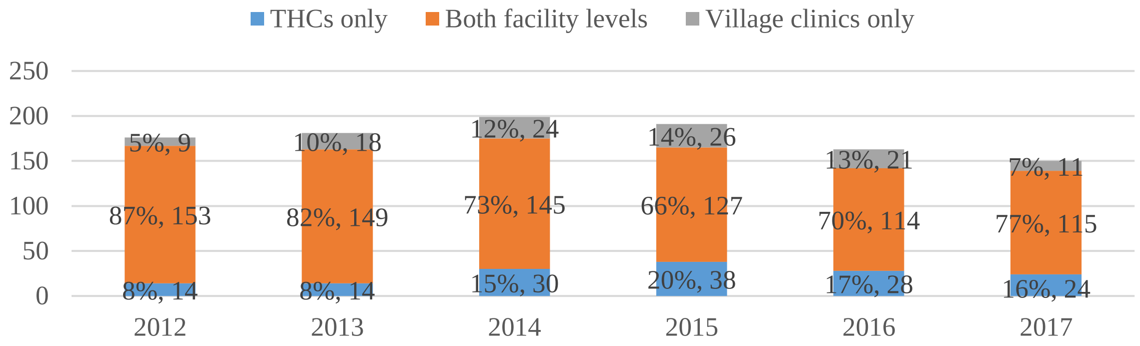

(b) Western medicines (%, n)

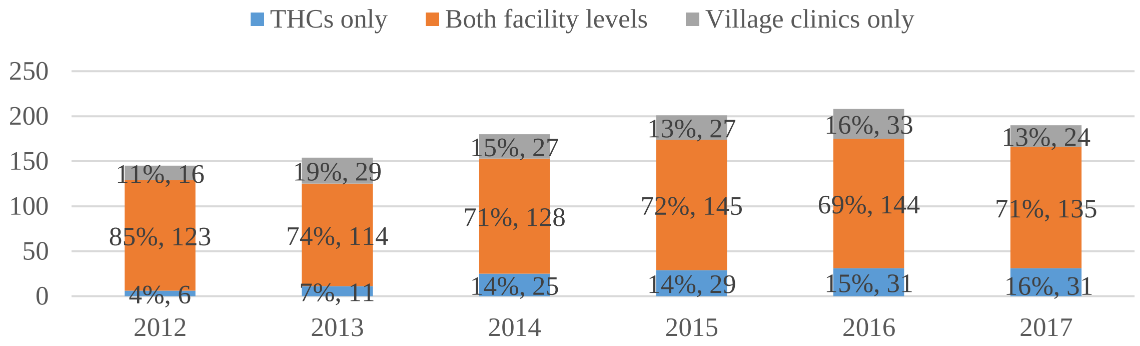

(c) Traditional Chinese medicines (%, n)

Figure S7. Originator brands of medicines in primary and secondary care facilities

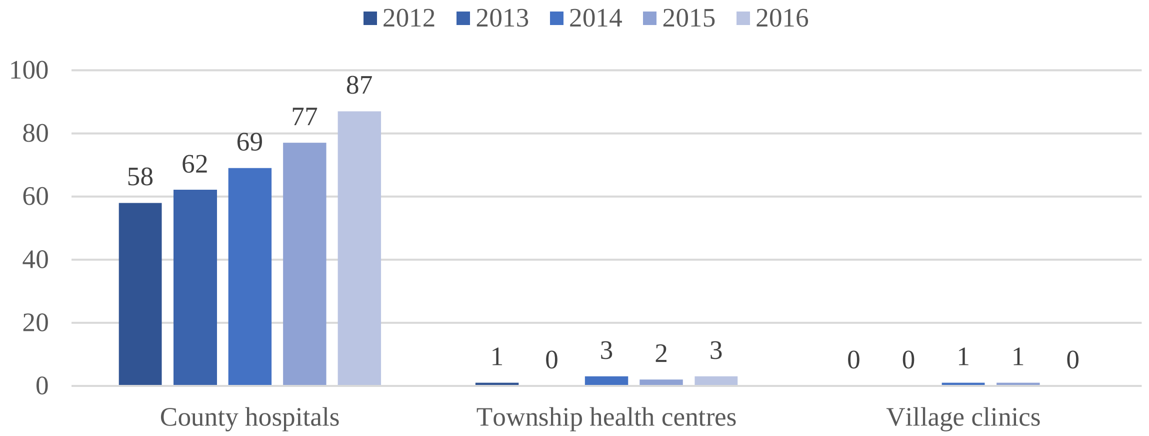

(a) Number of originator brands (n)

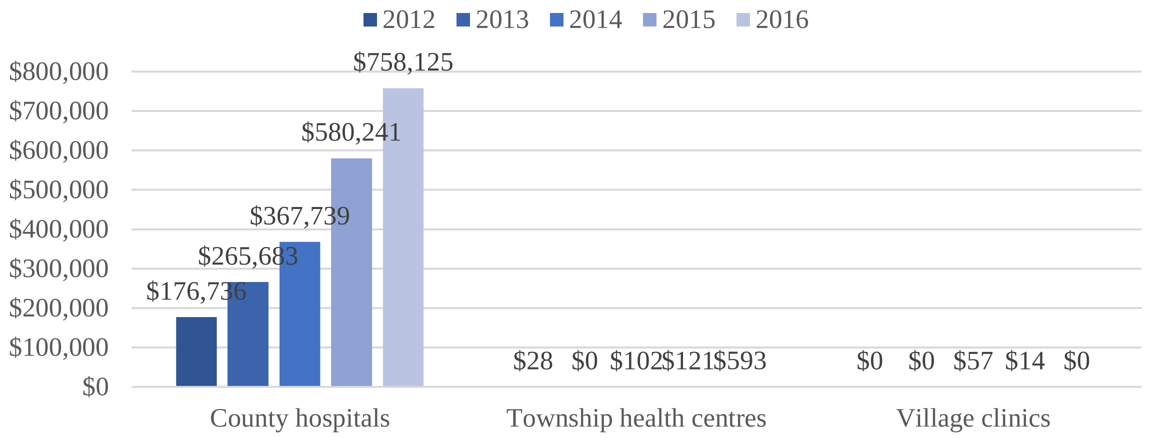

(b) Sales of originator brands (US$)
